# A quantitative evaluation of a qualitative risk assessment framework: Examining the assumptions and predictions of the Productivity Susceptibility Analysis (PSA)

**DOI:** 10.1371/journal.pone.0198298

**Published:** 2018-06-01

**Authors:** Adrian R. Hordyk, Thomas R. Carruthers

**Affiliations:** 1 Institute for the Oceans and Fisheries, AERL, University of British Columbia, Vancouver, British Columbia, Canada; 2 School of Veterinary and Life Sciences, Murdoch University, Perth, Western Australia, Australia; Aristotle University of Thessaloniki, GREECE

## Abstract

Qualitative risk assessment frameworks, such as the Productivity Susceptibility Analysis (PSA), have been developed to rapidly evaluate the risks of fishing to marine populations and prioritize management and research among species. Despite being applied to over 1,000 fish populations, and an ongoing debate about the most appropriate method to convert biological and fishery characteristics into an overall measure of risk, the assumptions and predictive capacity of these approaches have not been evaluated. Several interpretations of the PSA were mapped to a conventional age-structured fisheries dynamics model to evaluate the performance of the approach under a range of assumptions regarding exploitation rates and measures of biological risk. The results demonstrate that the underlying assumptions of these qualitative risk-based approaches are inappropriate, and the expected performance is poor for a wide range of conditions. The information required to score a fishery using a PSA-type approach is comparable to that required to populate an operating model and evaluating the population dynamics within a simulation framework. In addition to providing a more credible characterization of complex system dynamics, the operating model approach is transparent, reproducible and can evaluate alternative management strategies over a range of plausible hypotheses for the system.

## Introduction

Fishery management agencies are typically responsible for numerous stocks of diverse life history often distributed over a wide geographical area and exploited by various modes of fishing. Given limited resources, data-intensive quantitative stock assessments are not available for most fish stocks [1]. Additionally, fishing gears are often poorly selective and many non-target species are caught and discarded or incidentally killed by fishing gear. The impacts of fishing on non-target species has become increasingly recognized, and has supported the move away from traditional single-species assessments in favour of broader ecosystem-based fisheries management (EBFM) [2,3]. For example, the Magnuson-Stevens Fisheries Act [4] requires that US fisheries management plans aim to minimize bycatch (including non-target fish species, sea turtles, sea birds, and marine mammals), or where unavoidable, minimize bycatch mortality. Similarly, the Australian Environment Protection and Biodiversity Conservation Act 1999 (EPBCA) [5] requires managers to demonstrate that fisheries are ecologically sustainable. In response to these demands, numerous risk assessment methods, both qualitative, for example the risk assessment frameworks developed in Australia for determining ecological sustainable development (ESD) [6–9], and quantitative, such as Sustainability Assessment for Fishing Effects (SAFE) [10,11], have been developed with the aim of evaluating the risk of over-exploitation, declines in stock biomass to low levels, or other undesirable consequences of fishing on target and non-target species.

Stobutzki et al. [12] developed a risk assessment framework to determine the sustainability of trawling with respect to bycatch species in the Australian Northern Prawn Fishery (NPF). This was one of the first fisheries attempting to meet the EPBCA requirements and demonstrate its ecological sustainability. The method recognized two primary properties of a bycatch population that determine the sustainability of fishing: the ability to recover after being depleted (recovery), and the vulnerability to mortality caused by the fishery (susceptibility). Various attributes relating to susceptibility (e.g., position in the water column, post-capture survival, and range), and recovery (e.g., probability of breeding, maximum size, and reproductive strategy) were identified. Each species was scored into one of three risk categories for each attribute. The criteria were weighted by relative importance, determined by consensus through expert judgment, and a weighted average score calculated for both the susceptibility and recovery attributes [12]. The susceptibility and recovery scores for each species were plotted on a scatterplot, risk was calculated as a combination of equally weighted susceptibility and recovery scores, and the species were ranked on their overall risk to fishing [12,13].

A key advantage and driver behind the approach developed by Stobutzki et al. [12] was the ability to rapidly assign relative risk for a large number of by-catch species, and to provide management and research advice for a complex fishery. For example, Stobutzki et al. [12] used the method to rank 411 bycatch species in the NPF. Likewise, Milton [13] used the method to evaluate the relative risk of 13 species of sea snake, Stobutzki et al. [14] evaluated 56 species of elasmobranch in the same fishery, and Feitosa et al. [15] used a method based on Stobutzki et al. [12] to rank the relative risk of 19 species of ornamental reef fish caught in a trap fishery in Brazil.

Hobday et al. [8,16] developed the risk assessment methodology of Stobutzki et al. [12,14] into a method known as Productivity Susceptibility Analysis (PSA), and positioned this method as the second tier in a hierarchical ecological risk assessment framework (Ecological Risk Assessment for the Effects of Fishing; ERAEF). The three-tier approach begins with a qualitative consequence analysis that uses expert judgment to determine the likely impact a fishery has on bycatch species. Species that are determined to be at some risk to fishing are then evaluated in the second tier of the framework, the PSA. Species ranked as medium or high risk in the PSA are then assessed at the third level of the ERAEF hierarchy, a conventional stock assessment that aims to quantitatively evaluate risk. A principal aim of the ERAEF is to use the PSA to identify species at highest risk and prioritize assessment and management at Level 3 on these stocks [8].

Following Stobutzki et al. [12], Hobday et al. [8] assume that the vulnerability of a species to over-exploitation by a fishery is determined by two properties: productivity—the life history characteristics which determine the intrinsic rate of population increase, and susceptibility—interactions between population and fishing dynamics that affect the impact of the fishery on the stock. They define 7 productivity attributes and 4 susceptibility attributes. Three risk categories are defined for each attribute, with a corresponding numerical score of 1 (high productivity or low susceptibility; low risk), 2 (medium productivity or susceptibility; medium risk), and 3 (low productivity or high susceptibility; high risk). For example, species with an age at maturity greater than 15 years are considered to have low productivity and high risk and are scored 3, species with an age of maturity less than 5 years are scored as highly productive and low risk (1), and those that fall between these values are rated as medium productivity and risk (2) [8]. Likewise, species that have low fecundity (< 100 eggs per year) are defined as high risk (3), those with 100–20,000 eggs per year are medium risk (2), and species that produce more than 20,000 eggs per year are scored as low risk (1). Hobday et al. [8] note that their cut-off values for the risk categories were developed for an Australian fishery and suggest that the cut-off scores should be modified or tuned for species in other regions.

A numerical score of 1, 2 or 3 is assigned to each of the 7 productivity attributes, and the arithmetic mean of the productivity attributes is used to determine an overall productivity score (*P*). Scores are assigned to each susceptibility attribute in the same way as the productivity criteria, with a value of 3 representing high risk and 1 representing the lowest risk. The overall susceptibility score (*S*) is assumed to be a multiplicative function of the individual susceptibility attributes, and is therefore calculated as the geometric mean of the susceptibility attributes. The total vulnerability (*V*) or risk score is then determined by calculating the Euclidean distance from the origin:

$$V=\sqrt{P^{2}+S^{2}}$$ (1)

**Fig 1:**
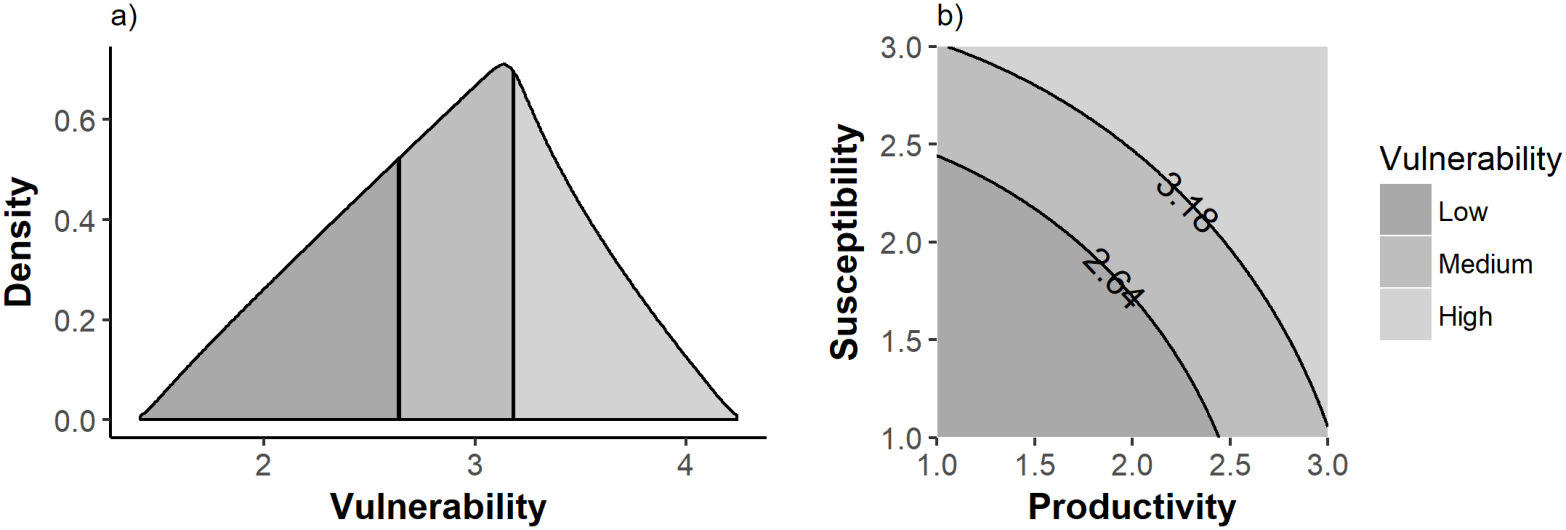
Distribution of PSA vulnerability scores and relationship between productivity, susceptibility, and vulnerability. The overall vulnerabilty score and risk categories used in the standard PSA (16), a) the three risk categories based on the assumption of equal density of the vulnerability scores and b) how the vulnerability score is determined from the total productivity and susceptibility scores.

This results in a vulnerability score ranging from 1.41 (all attributes scored 1) to 4.24 (all attributes scored 3) [17]. Assuming that all productivity and susceptibility scores are equally likely, a third of the *V* values will be lower than 2.64 and a third above 3.18 (Fig 1a), and these thresholds are used to define risk categories: ‘Low’, ‘Medium’ and ‘High’ (Fig 1b) [16].

Although the PSA of Hobday et al. [8,16] was designed as a tool to evaluate bycatch species, in recent years it has been increasingly applied to determine risk for targeted fish stocks. For example, Rosenberg et al. [18] made some alterations to the PSA and used it to discriminate risk for setting buffers in the annual catch limits for 169 federally managed stocks in the U.S. In 2008 a working group from the U.S. National Marine Fisheries Service (NMFS) developed an extended version of the PSA and applied it to 162 federally managed fisheries in the U.S [19,20]. For simplicity, we refer to the Hobday et al. [8,16] PSA as ‘standard PSA’ (sPSA) and that of Patrick et al. [19,20] as ‘extended PSA’ (ePSA).

The ePSA differs from the sPSA in several ways. Firstly, Patrick et al. [19,20] revised the productivity and susceptibility criteria, which resulted in 10 productivity attributes, including many of those used by Hobday et al. [8,16] and some additional attributes: population growth rate (*r*), von Bertalanffy growth parameter (*K*), and the natural mortality rate (*M*). They also modified the reproductive strategy criterion used by Hobday et al. [16] to include two attributes: breeding strategy, based on Winemiller’s [21] index of parental investment, and recruitment pattern, based on the expectation of successful recruitment events. The ePSA includes 12 susceptibility attributes, separated into two sub-categories: catchability and management. The 7 catchability attributes were based on Hobday et al. [16] but with the addition of criteria on the value of the fishery and fish behaviour including seasonal migration. The 5 management criteria included estimates of current fishing mortality relative to natural mortality (*F*/*M*), management strategy, estimate of spawning biomass relative to reference points (e.g., depletion), survival of released fish, and the impact of the fishery on the habitat.

Patrick et al. [19,20] noted that not all productivity and susceptibility attributes are equally valuable in determining the vulnerability of a stock and chose to use a weighting scheme where all attributes were assigned a default weight of 2 and weights for particular attributes could be adjusted with each application to reflect their perceived importance within a fishery. For example, in an application of the ePSA to highly migratory sharks in the Atlantic they chose to assign the highest weight of 4 to the intrinsic rate of increase (*r*) attribute, 3 to fecundity and natural mortality criteria, and 0 to the recruitment attribute [19]. Where there is insufficient information to assign a risk value to an attribute, the sPSA uses a precautionary approach and the attribute is scored as high risk [16]. Patrick et al. [19] followed a different approach and chose instead to leave missing attributes out of the analysis and developed a data quality scoring system to aid in the interpretation of the ePSA result.

While the sPSA calculated the total susceptibility score as the geometric mean of the susceptibility attributes [16], the ePSA used a weighted arithmetic average [19]. The scoring for the productivity attributes in ePSA was the reverse of sPSA, such that high values of productivity (i.e., 3) correspond with low risk and low productive attributes were scored 1, and the total vulnerability score (*V*) calculated as:

$$V=\sqrt{{\left(P-3\right)}^{2}+{\left(S-1\right)}^{2}}$$ (2)

Hobday et el. [8] broadly define risk as ‘the probability that a (specified) fishery management objective is not achieved’ and note that the intention of their PSA is to evaluate the relative risk of different species within a fishery. Others have used a more explicit definition of risk. For example, Patrick et al. [19] report stocks that receive a high vulnerability score from the ePSA are the most vulnerable to overfishing. Similarly, the NOAA toolbox claims that the ePSA has been designed to ‘specifically assess the vulnerability of U.S. fish stocks from becoming overfished (*B*_CURRENT_ < ½*B*_MSY_) or undergoing overfishing (*F*_CURRENT_ > *F*_MSY_)’ [22]. Other applications of the ePSA also make similar claims. Osio et al. [23] state that ‘the vulnerability of a stock is directly related to overfishing and is defined as a function of its productivity and susceptibility’. In contrast, other researchers believe that the PSA should only be used to provide relative vulnerability scores and should not be used to argue for or against the sustainability of a fishery [24].

Despite the application of the PSA to inform the management and research priorities of over 1000 targeted and by-catch fish stocks and other stocks including elasmobranches, sea turtles, and marine mammals (Table 1), it appears that there have been few attempts to quantitatively evaluate and validate the methodology. A recent study compared the performance of sPSA with the SAFE method, and the two risk assessment methods against the results of quantitative stock assessments [25]. The SAFE method is similar to the PSA but uses attributes at continuous measurement scales and calculates risk in terms of fishing mortality (*F*) relative to *F*_MSY_. This study found that the sPSA was precautionary compared to both SAFE and the assessment results, with a high proportion of false positives [25]. This study made the comparison of PSA by assuming that the results of the quantitative stock assessments were ‘true’. Therefore, while the results may suggest that the PSA is precautionary compared to typical stock assessments (at least for data-rich stocks) it was not able to quantitatively evaluate the accuracy of the PSA or the correlation between the PSA vulnerability score and the risk of over-exploitation or stock collapse.

**Table 1:** A summary of the species included in 23 applications of the productivity-susceptiblity analysis. The number of stocks/populations evaluated using a version of the productivity-susceptiblity analysis in 23 applications of the methodology. This summary is not intended to be exhaustive but demonstrates that the PSA has been used for a large number of species and taxa, including marine mammals, sea birds, sea turtles, sharks, skates and rays, and teleosts.

Primary PSA Reference	Number of Stocks/Populations						Application Reference
	Marine Mammals	Sea Birds	Sea Turtles	Sharks	Skates and Rays	Teleosts	
[61]	20	21	7	50+	14	150+	[61]
[61]			4	26	2	34	[62]
[61]			3	8	1	17	[63]
[64]				1			[65]
[66]		70					[66]
[16,64]				11			[67]
[16]				11	18	49	[68]
[16]				11	1		[69]
[16]	14						[70]
[16]		14					[71]
[16]		41					[72]
[16]						102	[73]
[16]	3	1		5	11	46	[74]
[16,19]			7				[75]
[16,19]						7	[76]
[19]				6	15		[77]
[19]						60	[78]
[19]						21	[79]
[19]						151	[23]
[19]				5	17	63	[80]
[19]						90	[26]
[19]				2		21	[81]
[19]				54	13	98	[20]
**Total**	**37**	**147**	**21**	**190+**	**92**	**909+**	

It is unusual that in a quantitative scientific field, a qualitative method has been so widely used to inform management without being extensively tested to evaluate the theoretical consistency of its assumptions and its predictive capacity. The lack of objective testing is particularly important given that it could resolve a large number of diverging views on the appropriate way to calculate the productivity, susceptibility, and overall vulnerability scores. For example, some applications of the PSA have used weighting schemes [19,20,26] to adjust the contribution of individual attributes to the overall vulnerability score. Others considered using weights but decided against it as they believed all attributes were equally important [24,27], make little difference to the overall vulnerability score [28], or because ‘there would need to be a consistent means of assigning weights (i.e. as determined through simulation modeling)’ [18]. Likewise, some believe that the practice of scoring missing attributes in the highest risk category will bias the results towards false positives [19,24], while advocates of this approach point out that this method means that additional information can only ever decrease the vulnerability score and never increase it [18]. Another example of inconsistency in applications of the PSA is the calculation of the overall susceptibility score, using either the geometric mean [8] or the arithmetic mean [19]. Proponents of the former approach note that a low score in one susceptibility attribute (e.g., spatial overlap of fishery and stock) can effectively make a stock invulnerable to fishing, while advocates of the latter believe this approach is likely to underestimate susceptibility [24]. Maintaining consistency in scoring of the attributes across a wide range of species has also been recognized as an important challenge [24,26].

Acknowledging the varying subjective interpretations of PSA, there are reasons to question its assumptions and structure. For example, while it is generally accepted that species with low fecundity are not highly productive, there is strong evidence against the idea that highly fecund species are less vulnerable to over-exploitation [29–31]. Furthermore, although the PSA calculates the overall productivity score additively as the mean of individual attributes, is it widely recognized that life history parameters of fish stocks are often highly correlated and non-linearly related to productivity [31,32]. Likewise, there appears to be little evidence in fisheries dynamics literature to suggest that it is generally appropriate to calculate overall vulnerability of a population to fishing as a linear function of productivity and susceptibility attributes. Consequently, it appears that several assumptions of the PSA methodology may be at odds with important fundamentals of non-linear dynamical systems such as fisheries.

Many of these limitations of the PSA have been recognized previously, and there have been various attempts to address these shortcomings by adding or removing attributes, using alternative methods to calculate overall the vulnerability score, exploring different weighting schemes, or comparing the PSA to other risk assessment methods [19,24,25,26,28]. However, these are fundamentally quantitative issues and as such it is not clear how they can be resolved by further discussion and expert opinion. An appropriate and relatively simple solution is to use quantitative simulation modelling with reproducible fisheries population dynamics models based on accepted fisheries science principles, to evaluate the assumptions and efficacy of methods that are intended to provide advice for fisheries managers [18,33].

The aim of this paper is to quantitatively evaluate the PSA as a tool for determining the risk of over-exploitation of fish stocks. Several interpretations of the PSA are mapped to a conventional age-structured fisheries dynamics model and alternative scenarios of stock status and future exploitation rates are explored to identify the conditions where the PSA is an effective tool for predicting risk of failing to meet fishery management objectives. The four main assumptions of the PSA are examined: 1) risk is an additive function of equally weighted total productivity and susceptibility scores, 2) the individual productivity and susceptibility attributes are equally weighted, 3) the individual attributes are linearly related, and 4) the PSA vulnerability score is a reliable predictor of the risk of failing to meet management objectives. Finally, we comment on the appropriateness of qualitative risk assessment tools to inform fisheries management and provide recommendations for alternative approaches.

## Methods

Each attribute and risk category of the sPSA [8] and the ePSA [20] was mapped to ranges of values for parameters in a conventional age-structured fisheries dynamics model. For many of the attributes this was straightforward. For example, the first productivity attribute, the average age at maturity, corresponds directly to the age at maturity in the population dynamics model. However, other attributes were more difficult to interpret and assign quantitative values. Consequently, it was not possible to include all attributes, and others were modified to directly correspond to population dynamics parameters. Operating models were specified that described the fishery and population dynamics based on the productivity and susceptibility attributes from the PSA. These operating models were used to calculate the probability of the fishery failing to meet various management objectives. The quantitative estimates of risk were then compared against the vulnerability scores from the PSA, and the influence of the individual PSA attributes on the risk to the fishery was examined.

### Modified PSA

Two quantitative interpretations of PSA were developed corresponding to the sPSA [8] and the ePSA [20]. Following Hobday et al. [8], consistent scoring for both productivity and susceptibility attributes was assumed such that a score of 1 represents lowest risk and 3 the highest risk. In some PSA applications a weighting scheme has been adopted to control the importance of different attributes on the overall vulnerability score [20]. These weightings have been decided by expert opinion and group consensus and there was no way to reproduce this subjective process objectively. Consequently, all productivity and susceptibility attributes were weighted equally in this analysis.

#### Standard PSA

In general, the same attributes and risk categories as Hobday et al. [8] were used with some important differences that are described below.

#### Productivity attributes

The first productivity attribute, the average age of maturity, could be directly mapped to the population dynamics model (Table 2). The same risk categories as Hobday et al. [8] were used and the range for age of maturity was set to 1 and 30 years (Table 2). For example, when modelling a low productivity species (high risk) with respect to this attribute, ages of maturity were uniformly sampled between 15 and 30 years. Conversely, when generating an operating model for highly productive species from the PSA, age of maturity was sampled from a uniform distribution between 1 and 5 years.

**Table 2:** The productivity and susceptibility attributes and the risk categories used for the standard PSA (sPSA) and the extended PSA (ePSA).

	Productivity Attribute	Risk Category		
		Low (1)	Medium (2)	High (3)
**sPSA**	Age at maturity	1–5	5–15	15–30
	Maximum age	5–10	10–25	25–60
	Maximum size	20–100	100–300	300–500
	Size at maturity	< 40	40–200	> 200
	Steepness (h)	0.6–0.95	0.4–0.6	0.21–0.4
**ePSA**	Maximum rate of increase (r)	> 0.5	0.16–0.5	< 0.16
	von Bertalanffy K	> 0.25	0.15–0.25	< 0.15
	**Susceptibility Attribute**			
**sPSA**	Availability^1^	< 0.25	0.25–0.50	> 0.50
	Encounterability^2^	< 0.2	0.2–0.8	> 0.8
	Selectivity	L_c_^3^ > L_m_^4^	0.5L_m_ < L_c_ < L_m_	L_c_ < 0.5 L_m_
	Discarding and discard mortality^5^	High discarding Low discard mortality	Moderate discarding Moderate discard mortality	Low discard High discard mortality
**ePSA**	Depletion (*SB*^6^/*SB*_0_)	> 0.4 < 0.8	0.25–0.4	< 0.25

^1^ Fraction of stock available to fishing

^2^ Vulnerability of the largest sized individuals in the population (degree of dome-shaped selectivity)

^3^ Length at 50% selectivity

^4^ Length at 50% maturity

^5^ See text for details

^6^ Spawning biomass

The second productivity attribute was the expected maximum age (*A*_max_). The natural mortality rate (*M*) was assumed to be related to *A*_max_ by [34]:

$$M=-\frac{\text{log}\;\left(0.01\right)}{A_{\text{max}}}$$ (3)

Sixty years was chosen as the upper bound for the maximum age (i.e., 25 < *A*_max_ < 60 years = low productivity) and a lower bound of 5 years (i.e., 5 < *A*_max_ < 60 years = high productivity; low risk), as this includes the lifespan of most marine fishes [35] (Table 2).

The average maximum size was modelled as the asymptotic length parameter in the von Bertalanffy growth model (*L*_∞_).The risk categories of Hobday et al. [8] were used with an upper bound of maximum length of 500 cm (i.e., 300–500 cm = low productivity) and a lower bound of 20 cm (i.e., 20–100 cm = high productivity) (Table 2). Similarly, the average size of maturity followed the risk categories of Hobday et al. [8] and could be directly mapped as a biological parameter in the fisheries dynamics model.

The sPSA literature defines the three risk categories of the reproductive strategy attribute as: live bearers (and birds) (low productivity; high risk), demersal egg layer (medium productivity and risk), and broadcast spawners (high productivity; low risk), but provide no additional rationale for this categorization [8,16,17]. Stobutzki et al. [12] use reproductive strategy as a proxy for relative fecundity between species and note that broadcast spawners generally produce more young than live bearers and animals that brood their young. They conclude that broadcast spawners have the capacity to recover faster from low population sizes, and are therefore considered lower risk, but provide no evidence to support these claims. Empirical evidence supports a relationship between reproductive strategy and total fecundity, with broadcast spawners typically producing many small sized eggs, while demersal eggs layers and live bearers produce fewer and larger eggs and young respectively [36,37]. Hobday et al. [8] include fecundity as a separate productivity attribute, with more fecund species considered more productive and at lower risk. Some researchers have suggested that a relationship may exist between fecundity and the inter-annual variation in recruitment that might influence risk of stock declines. However, Mertz and Myers [38] examined this relationship and found little evidence to support it. Rickman et al. [39] on the other hand, examined additional data with a greater contrast in fecundity and found evidence that stocks with higher fecundity have more variable recruitment. However, while the general relationship was shown to exist, the high variability in the relationship prevents accurate prediction of the magnitude of recruitment variation from information on fecundity. Consequently, while it may be tempting to relate the productivity attribute of ‘fecundity’ to recruitment variability in a population dynamics model, there is little guidance how to do this appropriately.

Without a coherent rationale for the inclusion of these attributes and risk categories in the PSA, it is not clear how to relate the three risk categories of reproductive strategy and fecundity to population dynamics parameters. Instead these attributes were related to the steepness (h) of the stock-recruitment relationship as a replacement attribute (Table 2). While this does not exactly replicate the attributes of fecundity and reproductive strategy in the PSA, steepness describes the rate of population increase at low stock sizes [40], and therefore may represent the intent of these two attributes [41]. The three steepness categories were low productivity (h < 0.4), medium productivity (0.4 > 0.6) and high productivity (h > 0.6) (Table 2).

The last productivity attribute in the sPSA is mean trophic level, divided into three risk categories: > 3.25 (low productivity; high risk), 2.75–3.25 (medium productivity/risk), and < 2.75 (high productivity; low risk). Like the previous two attributes, there was no information in Hobday et al. [8] or the previous literature on the development of PSA [12] describing the rationale for this attribute or how trophic level is hypothesized to be related to productivity. Trophic level represents the position of a species within a hierarchical food web, and, as predators usually consume prey that are smaller than themselves, is often found to be correlated with size [42,43]. However, maximum size is already included as a productivity attribute, and in the absence of a defensible means of relating trophic level to the population dynamics model, it was not included in the analysis.

#### Susceptibility attributes

Hobday et al. [8] divide their first susceptibility criterion, availability, into two choices: the overlap of species range with the fishery, and the range of global distribution of the species and note that the first option is preferable. The first approach was followed here and simulated as a two-area model, where the fraction of the stock in the first area experienced no fishing mortality. The same risk categories as Patrick et al. [19] were adopted. For example, if less than 25% of a stock is in the fished area, the fishery is considered low risk and given a score of 1, while fisheries that operate on more than 50% of the stock are scored as 3, high risk (Table 2). The efficacy of spatial closures on fishery dynamics depends strongly on the rate of movement of the species. In this analysis, the stock was assumed to be fully mixed between the two areas.

The second susceptibly attribute, termed ‘encounterability’, aims to define the interaction between the fishing gear and the stock. Hobday et al. [8] define the three risk categories as: low-, medium- and high-overlap with the fishing gear and suggest two options for this criterion: habitat or depth check. This analysis followed Patrick et al. [19] who interpret this criterion as vertical overlap of the fishery with the stock. In this analysis it was assumed that larger fish tend to be found in deeper water, and this attribute related to the vulnerability of the largest size individuals in a stock. For example, for stocks considered low susceptibility (1) larger individuals are not vulnerable to fishing while highly susceptible (3) are fully selected by the fishery at larger sizes (Table 2).

The size selectivity pattern of the fishery, in particular relative to the size of maturity, is important in determining the impact a fishery has on the risk of over-exploitation. Hobday et al. [8] score this attribute based on the mesh or hook size relative to the average size of maturity of the stock. This analysis follows the interpretation of the Marine Stewardship Council [44] and defines this according to 3 risk categories: low risk (1): length at capture (*L*_*c*_) is greater than length of maturity (*L*_*m*_), medium risk (2) 0.5*L*_*m*_ < *L*_*c*_ < *L*_*m*_, and high risk (3) *L*_*c*_ < 0.5*L*_*m*_ (Table 2).

The final susceptibility attribute relates to discarding and post-capture mortality (8). This criterion relates to two distinct properties of the fishery: the overall fraction of the catch that is discarded (discard rate), a property of the fishing fleet, and the proportion that are discarded dead when (or soon after) released (discard mortality), a biological property of the stock. Consistent with the sPSA these are combined into a single criterion in this analysis, with three risk categories: high discarding and low discard mortality (low risk), moderate discarding and moderate discard mortality (medium), and low discarding and high discard mortality (high risk) (Table 2).

#### Extended PSA

A quantitative interpretation of ePSA was developed by including two additional productivity attributes, the intrinsic rate of population growth (*r*) and the growth parameter of the von Bertalanffy model (*K*), and one additional susceptibility attribute (depletion). The intrinsic rate of population growth represents the maximum growth rate of the population at a low stock size. This parameter is directly related to stock productivity and is a combination of many of the other productivity attributes. The value of *r* was calculated using the method of McAllister et al. [45]. The von Bertalanffy growth equation is the most commonly applied model to describe the growth of fish and is used in the population dynamics model of this analysis. The risk categories of Patrick et al. [19] were adopted for both attributes (Table 2).

Patrick et al. [19] included several more susceptibility attributes in the ePSA, including migration patterns and schooling behavior, morphology affecting capture, value of the fishery, and management. These attributes were not included in this analysis, primarily because of the difficulty in objectively relating these attributes to the parameters of the population dynamics model. The current relative spawning stock biomass (depletion; SSB/SSB_0_) was included as an additional susceptibility attribute and was based on the same risk thresholds as Patrick et al. [19] (Table 2).

Although the ePSA includes fishery management as a susceptibility attribute, it was not included in this evaluation of the PSA. The basis for this decision was because the intention of this analysis is to examine the relationship between the vulnerability score from the PSA and the inherent risk of the stock declining to low levels under numerous scenarios [8] and active fishery management confounds this relationship. For example, consider an omniscient manager that can enforce exploitation precisely at target level (e.g. fishing at *F*_MSY_). In this ‘perfect’ management scenario there would be no relationship between productivity and susceptibility attributes and the risk of stock collapsing to undesirable levels, as the stock biomass would be maintained exactly at the target level. While clearly hypothetical, this highlights a highly questionable attribute of the fishery management attribute in the ePSA that could nullify the impact on risk of all other attributes combined.

### Fisheries dynamics model

The DLMtool R package [46] was used to simulate and evaluate the risk of failing to meet management objectives under the various biological and fishery management scenarios corresponding to the PSA. DLMtool uses a typical age-structured fisheries model to generate the population dynamics. We briefly describe the population dynamics model here and refer readers to [46] for full details of the model. The fisheries dynamics model includes parameters that describe the population biology and the behavior of the fishing fleet. We refer to these components as the stock object and fleet object respectively.

The stock object contains the information describing the biology of the modeled fish stock, such as growth, maturity schedule, natural mortality rate, and stock-recruitment relationship, as well as the current stock status (i.e., the level of depletion). The fleet object describes the interaction between the fishing fleet and the stock, and contains parameters that define the historical fishing mortality, the selectivity schedule, and discarding rate.

#### Simulating life histories with equivalent vulnerability

**Fig 2:**
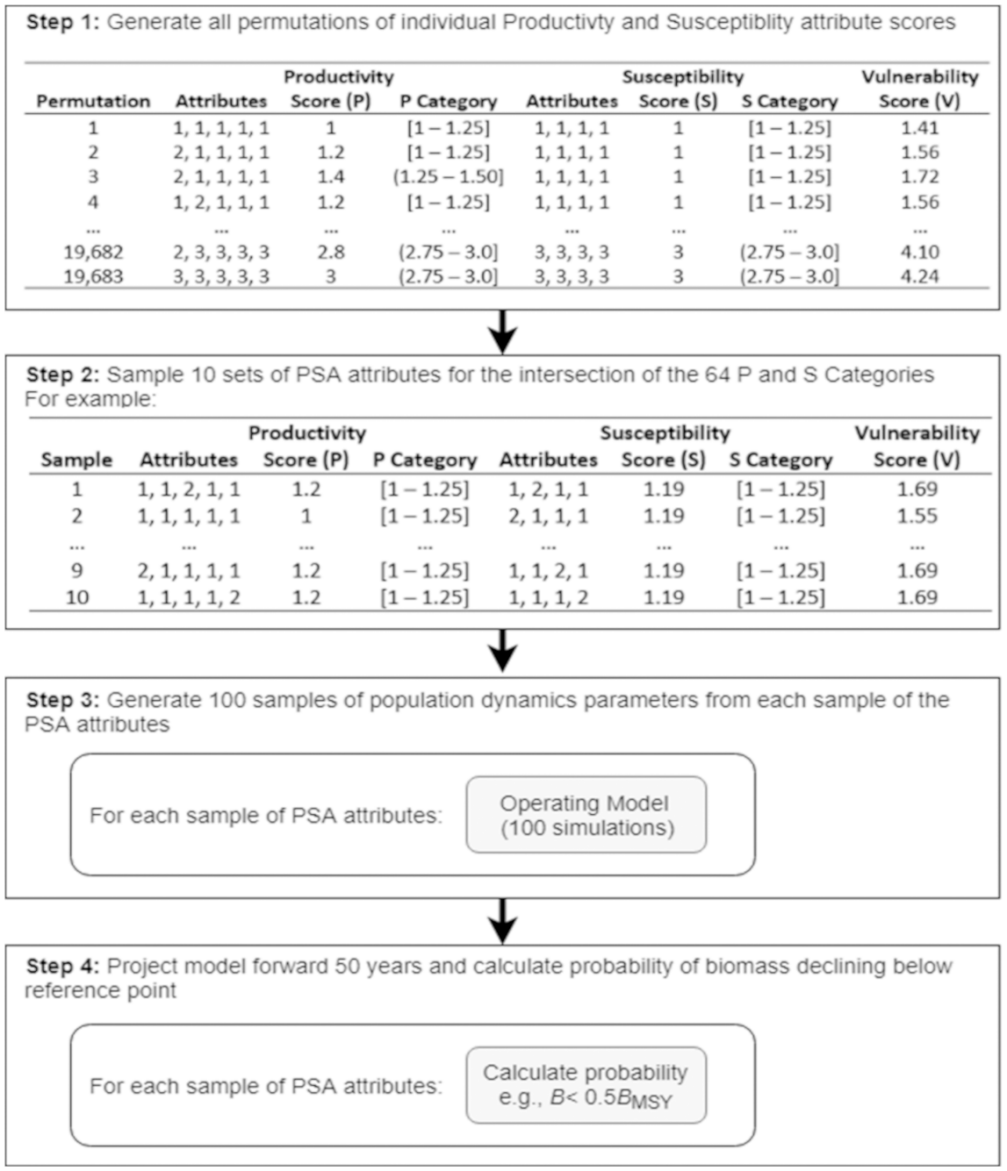
The four step process used to calculate risk of failing to meet a management objective for the different PSA scores. This process was used to evaluate both the standard PSA and the extended PSA.

Assuming each risk category is equally probable for the 9 productivity and susceptibility attributes of the sPSA, this constitutes over 19,000 scoring permutations. The additional 3 attributes in the ePSA increases the number of scoring combinations to over 500,000. It was not feasible to evaluate all permutations of the sPSA and ePSA attributes. Instead a four-step approach was used to sample the permutations of the PSA attributes, generate operating models, simulate the populations, and calculated quantitative estimates of risk:

1. All PSA scoring permutations were generated and the total productivity and susceptibility scores were calculated for each. This resulted in 19,683 and 531,441 scoring combinations for the sPSA and ePSA respectively. For each scoring combination, total productivity and susceptibility was categorized into 8 classes of equal width (1–1.25, 1.25–1.50, …, 2.75–3.0) leading to 64 possible categories of combined productivity and susceptibility. The number of permutations in each scoring category varied, with a median of 150 for the sPSA, and a median of 4,270 for the ePSA.
2. From each of the 64 categories, 10 sets of scores were sampled leading to 640 scoring combinations distributed evenly over the possible range of productivity and susceptibility.
3. Each of the 640 scores were mapped to corresponding fishery dynamics operating models.
4. For each operating model 100 simulations were carried out, projecting populations forward in time calculating the probability of the biomass declining below biological reference points at the end of the projection period (Fig 2). From these 100 projections it was possible to calculate risk metrics for the 640 PSA scoring combinations.

Some combinations of risk categories are not possible. For example, species in the low risk category for maximum age (maximum age < 10 years) cannot be scored as high risk for the age of maturity criterion (age of maturity > 15 years). Other less obvious correlations also exist among the risk categories. For example, the maximum rate of increase (*r*), a productivity attribute in the ePSA (Table 2), is a function of the other life history parameters (e.g., age of maturity, von Bertalanffy *K* parameter, and steepness), and therefore is expected to be correlated with the other attributes. Similarly, the von Bertalanffy growth parameter (*K*) can be determined analytically from values of asymptotic length and both age and size at maturity, and therefore only certain combinations of these parameters are plausible. The ratio of natural mortality (*M*) to the von Bertalanffy growth parameter (*K*) is often assumed to be close to 1.5, and rarely larger than 4 or smaller than around 0.3 [47]. Similarly, the relative size of maturity $\left(\frac{L_{m}}{L_{\infty }}\right)$ is usually between 0.3 and 0.9 for most fish species [47,48]. Life history theory and empirical evidence also suggests that the age of maturity is usually below 60% of the maximum age [48,49]. These empirical findings were used to exclude implausible scoring combinations.

For each simulation, the population was initialized in the unfished condition and simulated for 50 historical years. The depletion (*B*/*B*_*0*_) in the 50^th^ year was set as a parameter in the operating model. The sPSA was examined under three scenarios of historical exploitation where depletion in the final historical year was uniformly distributed between 0.025–0.25, 0.25–0.4 and 0.4–0.8. These scenarios are referred to as ‘Low’, ‘Medium’ and ‘High’ initial stock status, respectively. Current stock status is included as an attribute in the ePSA, therefore depletion was determined by the risk category for each sample, i.e., below 0.25 for high risk, 0.25 to 0.4 for medium risk and between 0.4 and 0.8 for low risk (Table 2). Density dependence was modelled with a Beverton-Holt stock-recruitment relationship, with steepness determined in each simulation by the relevant PSA attribute and risk category (Table 2). Process error in recruitment was assumed to be log-normally distributed with a coefficient of variation (CV) uniformly distributed between 0.3 and 0.9, and an auto-correlation coefficient uniformly distributed between 0 and 0.9.

#### Calculating risk

After the initial 50-year simulation, the model was projected forward for 50 years with three alternative scenarios of future exploitation intensity, an exploitation rate of 0.2, 0.4 and 0.6 respectively. The purpose for the alternative exploitation patterns was to evaluate the conditions where the PSA is a reliable predictor of the inherent risk of a population to fishing [8].

Risk for each analysis and three exploitation rate scenarios was calculated as the probability of failing to meet three different management objectives relating to biological sustainability: the probability of spawning stock biomass declining below 50% of *B*_*MSY*_ and below 10% and 20% of the unfished level. These risk metrics were calculated over the last 10 years of the simulation timeframe, i.e., the fraction of the 100 simulations where biomass was below the reference points in years 41–50 of the projection period. These metrics were chosen as the represent common reference points and measures of risk in international fisheries management agencies [50–52].

### Analysis of results

#### Risk is an additive function of total productivity and susceptibility scores

To examine the assumption that risk is linearly related and additive with respect to the overall productivity and susceptibility scores, the total productivity and susceptibility scores were binned into the same discrete classes described above and the probability of failing to meet the management objectives was calculated for each of the 64 combinations of productivity and susceptibility scores. This resulted in an 8 by 8 matrix for each variation of the PSA analyzed in the study. The expected relationship was generated using the same method, and all matrices were standardized to a minimum and maximum value of 0 and 1 respectively.

**Fig 3:**
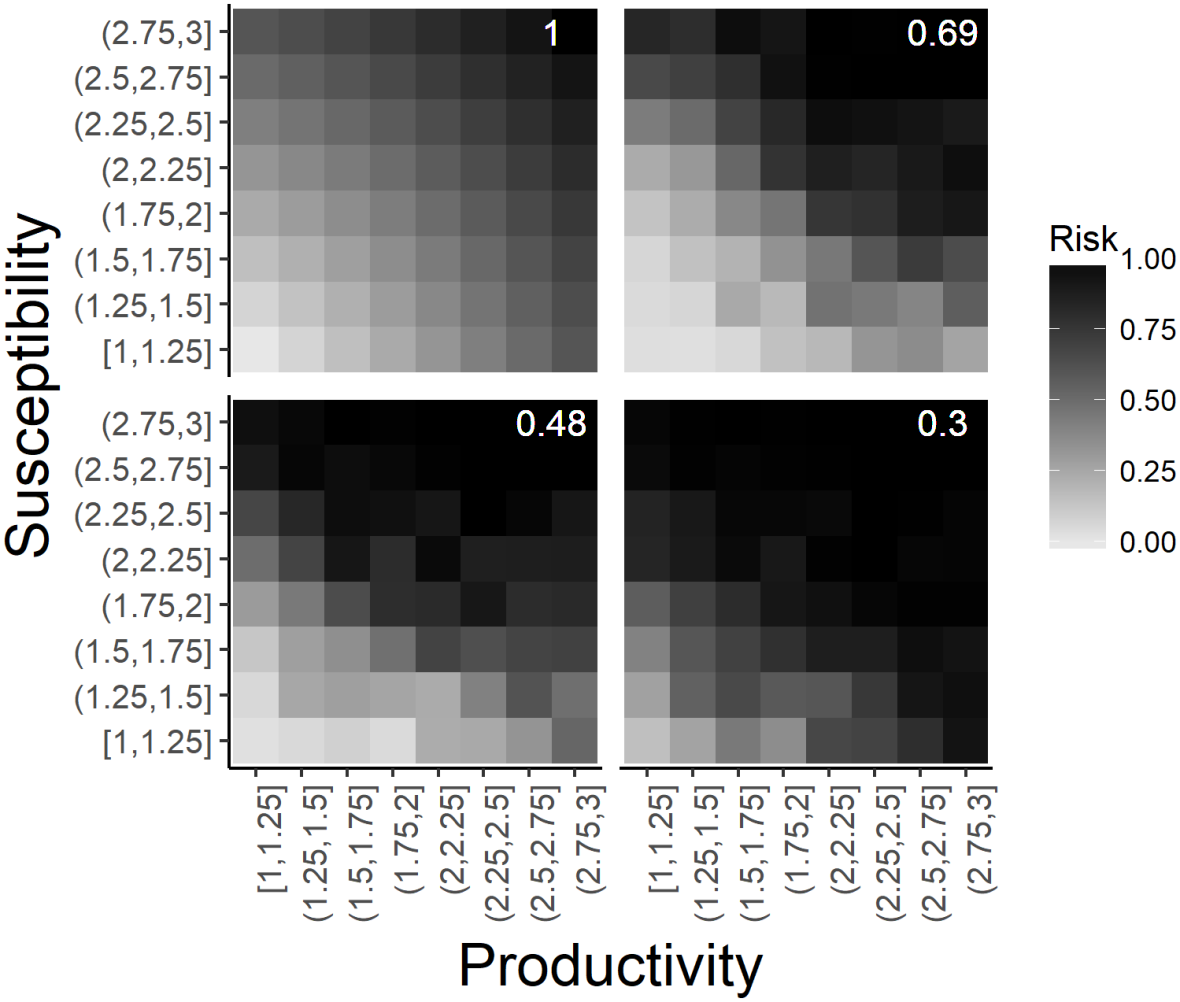
The assumed and observed relationship between productivity and susceptibility scores and risk. The relationship between the productivity and susceptibility scores and risk assumed by the PSA (top left) and the observed patterns for the analysis with the highest similarity score (top right; additive ePSA with low exploitation rate and B < 0.2B_0_ reference point), mean similarity score (bottom left; multiplicative sPSA with low initial stock size, high exploitation rate and B < 0.1B_0_ reference point) and the lowest similarity score (bottom right; multiplicative sPSA with high initial stock size, high exploitation rate and B < 0.2B_0_ reference point). Risk in each plot has been standardized to a minimum and maximum value of 0 and 1 and the similarity score is shown in white text in the top right corner of each plot.

The distance (*F*) between the expected (*E*) and observed (*O*) matrices was calculated using the Frobenius norm:

$$F_{A}=\sqrt{trace(AA^{*})}$$ (4)

where *A* = *E* − *O* and A* is the conjugate transpose of *A*, and *trace* is the sum of the main diagonal elements of AA*. The distance *F* was standardized to a similarity score such that a value of 1 represented an observed matrix identical to the expected pattern and a value of 0 represented the average score of 1,000 matrices where the elements were randomly assigned values between 0 and 1. The similarity score was used to quantitatively compare the different variations of the PSA evaluated in this study to the expected linear and additive relationship of the productivity and susceptibility scores and overall risk (see Fig 3).

#### Individual productivity and susceptibility attributes are equally weighted

The assumption of equal weighting of the individual productivity and susceptibility attributes was examined by fitting a multiple regression model with the risk measure as the predicted variable and the individual productivity and susceptibility attributes as predictors, and calculating the relative importance of each predictor variable using the relaimpo R package [53].

#### Individual attributes are linearly related

The PSA assumes that the individual productivity and susceptibility attributes are linearly related and additive. For example, if this assumption is valid, risk is expected to increase linearly as the scores of two attributes increase from 1 to 3, such that risk is lowest when both attributes are scored 1, highest when both are scored 3, and equal when the two attributes are scored 1 and 3 or 3 and 1 respectively. This assumption was evaluated by calculating the two-way interaction between the 12 individual productivity and susceptibility attributes of the ePSA and the quantitative measure of risk.

#### PSA vulnerability score is a reliable predictor of risk

The overarching principal of the PSA is that the vulnerability score is correlated with the risk of failing to meet management objectives. This was examined by plotting the PSA vulnerability score against the quantitative measure of risk for each simulation and examining the relationship between the two variables. These results were quantified in terms of accuracy and error rate by calculating the probability of correctly rating a fishery using the PSA. For example, non-governmental organisations (NGOs) such as Marine Stewardship Council [44] and Seafood Watch [54] use the multiplicative sPSA and follow Hobday et al. [16] by categorizing risk as: V < 2.64 = Low Risk, 2.64 < V < 3.18 = Medium Risk, and V > 3.18 = High Risk. They do not, however, define how these risk categories relate to the probability of failing to meet the management objectives. In this analysis, the probability of each risk measure was similarly divided into three equal categories (P < 0.33 = Low, 0.33 < P < 0.66 = Medium, 0.66 < P = High), and an error matrix was calculated for each variant of the PSA. The sensitivity (true positive rate) and overall accuracy was calculated using the methods in the caret R package [55]. All simulations and analyses were conducted using the R statistical environment [56].

## Results

Fig 3 shows the assumed relationship between the productivity and susceptibility scores and the overall risk (top left) and the observed relationship for three analyses with the highest, mean, and lowest similarity scores (top right, bottom left, bottom right, respectively). The observed patterns were similar to the assumed relationship where the risk scores were lowest when both productivity and susceptibility were in the lowest risk categories, and highest when they were in the highest risk categories. However, the observed pattern did not follow the PSA assumption of a linear increase in risk as the productivity and susceptibility scores increased. While the PSA assumes that the productivity and susceptibility risk scores are additive, that is, risk is symmetrical around the diagonal, this was not found to be the case for either the sPSA or ePSA. For example, in the case with the highest similarity score (top right Fig 3) risk was 3 times higher in the lowest productivity and highest susceptibility category (top left corner) compared to the reverse situation (lower right corner). In the analysis with the lowest similarity score (lower right Fig 3) risk was equivalently high for the highest productivity and lowest susceptibility risk categories (lower right corner), the lowest productivity and highest susceptibility risk categories (upper left corner), and the highest productivity and highest susceptibility risk categories (upper right corner).

These results suggest that the assumption of a linear and additive relationship between the productivity and susceptibility scores is not valid, and that the susceptibility score is of greater importance compared to the productivity score in determining the overall risk to the stock. Figures of the observed relationship between the productivity and susceptibility scores and the overall risk for all the analyses are shown in S1–S6 Figs and S7–S12 Figs for the sPSA and ePSA analyses respectively.

**Fig 4:**
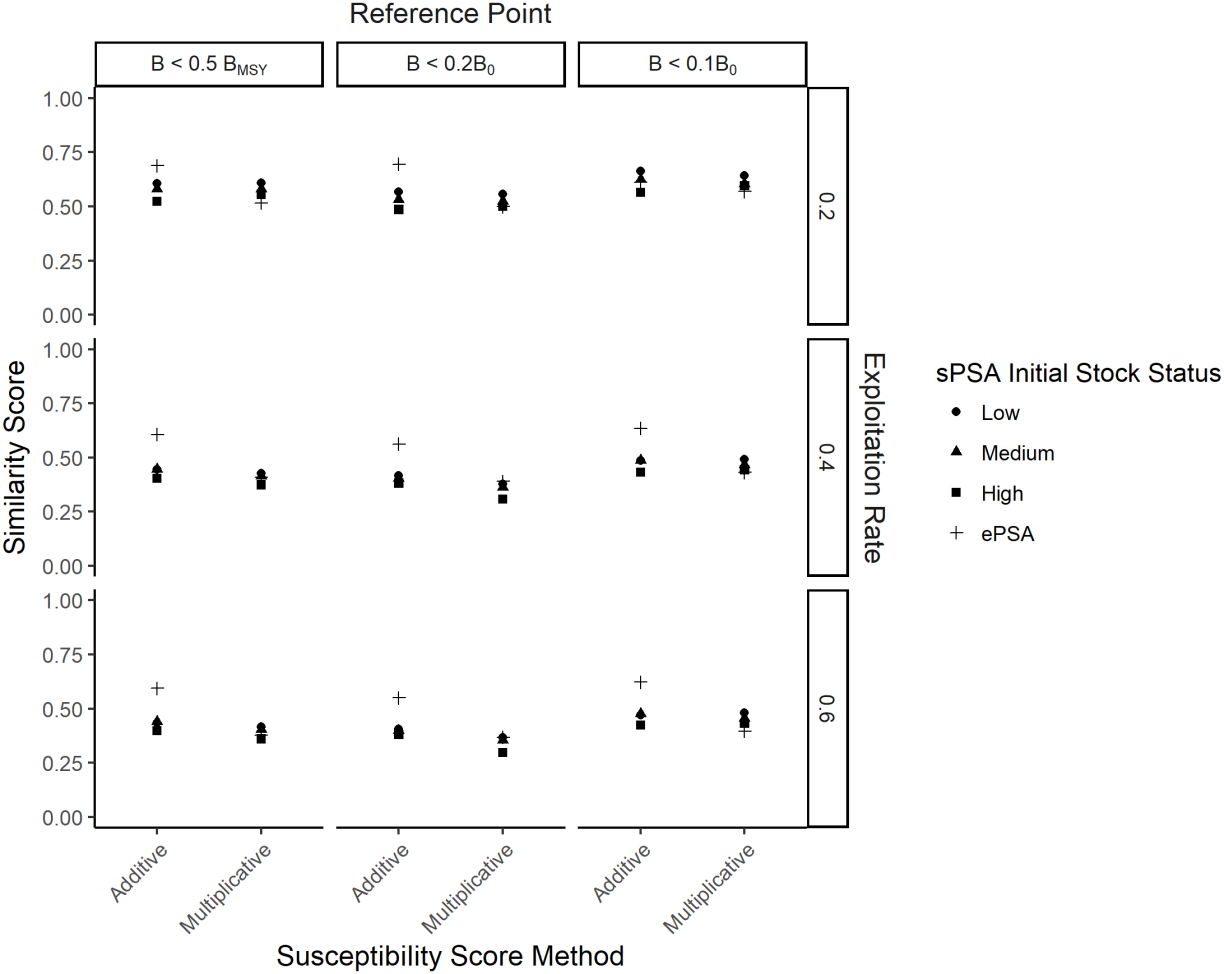
Similarity scores of the observed relationship between the productivity and susceptibility scores and risk compare to that assumed by the PSA. The similarity scores of the observed relationship between the productivity and susceptibility scores and risk compare to that assumed by the PSA for the standard PSA (sPSA) with low, medium and high initial stock status (black circle, triangle and square respectively) and the extended PSA (ePSA) for three quantitative measures of risk (columns) and three future exploitation rates (rows). The results are shown for both the additive and multiplicative method for calculating the overall susceptibility score (x-axis).

In general, ePSA was closest to the underlying assumptions of PSA and provided the closest re-creation of risk with respect to its productivity and susceptibility scores with a mean similarity score of 0.61 and a range of 0.54–0.69 (Fig 4). Similarly, the multiplicative method to calculate the overall susceptibility scores generally resulted in a lower similarity score compared to the additive method, for both the sPSA and the ePSA, although in many cases this difference was only marginal (Fig 4). The sPSA was most similar to the predicted pattern (similarity score = 0.66) when the initial stock size was low, exploitation rate was low, and the risk measure of B < 0.1B_0_ was used (Fig 4). The similarity score for the sPSA tended to decrease as the initial stock status and the exploitation rate increased. The closest matching ePSA, with a similarity score of 0.69, occurred when exploitation rate was low and the risk measure of B < 0.2B_0_ was used (Fig 4). Similar to the sPSA, the similarity score declined as the exploitation rate increased.

**Fig 5:**
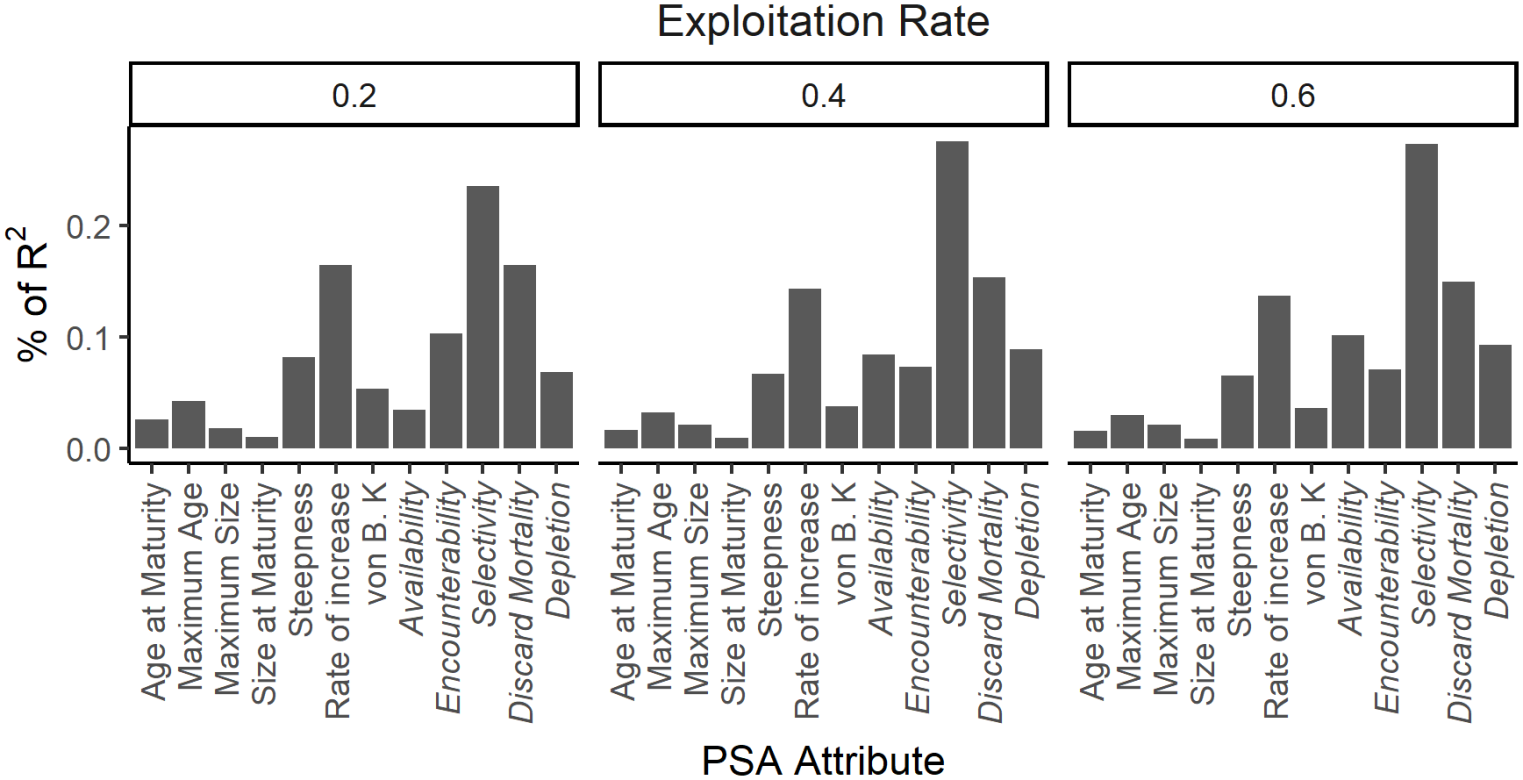
The relative contribution of the productivity and susceptibility attributes to overall risk for the exended PSA. The relative contribution of the 7 productivity and 5 susceptibility (italics) attributes of the additive extended PSA in explaining the variation of spawning biomass (B) at the end of the projection period being below 0.5B_MSY_ under conditions of low, medium, and high exploitation rates.

**Fig 6:**
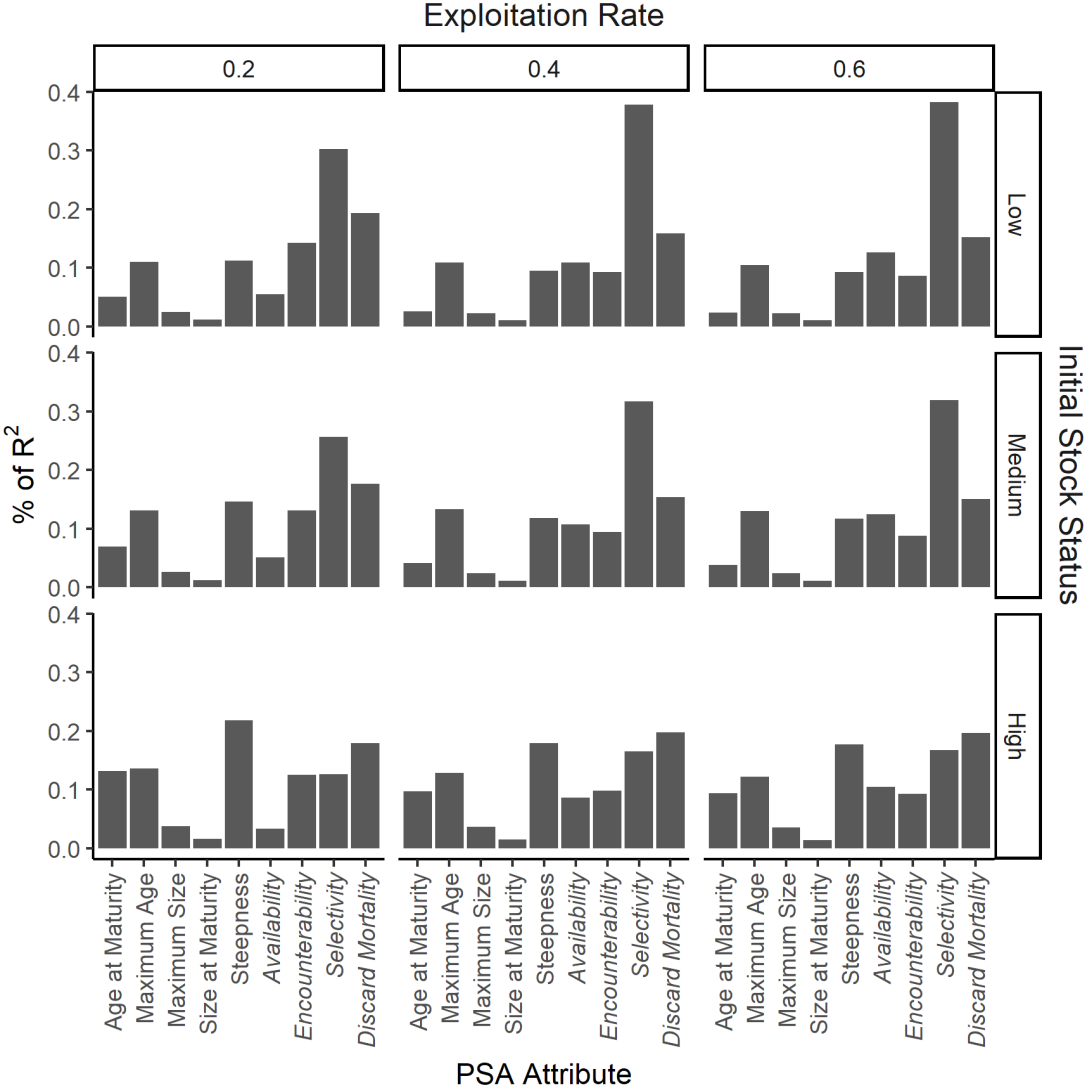
The relative contribution of the productivity and susceptibility attributes to overall risk for the standard PSA. The relative contribution of the 5 productivity and 4 susceptibility (italics) attributes of the additive standard PSA in explaining the variation of spawning biomass (B) at the end of the projection period being below 0.5B_MSY_ under conditions of low, medium and high initial stock size (rows) and low, medium, and high exploitation rates (columns).

For the additive ePSA and the B < 0.5B_MSY_ reference point, in all three exploitation rate scenarios the Selectivity attribute contributed the greatest to explaining the variability in risk, followed by Discard Mortality and the Rate of Increase (*r*; Fig 5). In contrast, attributes such as the Size of Maturity, Maximum Size, and Age of Maturity explained very little of the variability in risk. The result for the additive sPSA and the B < 0.5B_MSY_ reference point show that at low initial stock size the Selectivity attribute explained ~30% of the variance, with Discard Mortality the second most important attribute (Fig 6). A similar pattern was observed in when initial stock size was at medium levels. The results were more varied at high initial stock size, with Steepness the most important attribute when exploitation rate was 0.2, and the second most important when exploitation rate was 0.4 and 0.6. In all scenarios the Size of Maturity was ranked the least important attribute (Fig 6). Although the results varied marginally depending on the risk measure and the method to calculate the susceptibility score, the importance ranking of the attributes was consistent (S13–S22 Figs).

These results provide a strong theoretical case against the assumption that the individual productivity and susceptibility attributes contribute equally to risk. In contrast, the results suggest that the susceptibility attribute Selectivity (size of capture relative to the size of maturity) is the strongest predictor of risk. The most important productivity attributes are, in the case of the ePSA, the intrinsic rate of increase and the steepness of the stock-recruitment relationship, and for the sPSA, steepness and maximum age.

**Fig 7:**
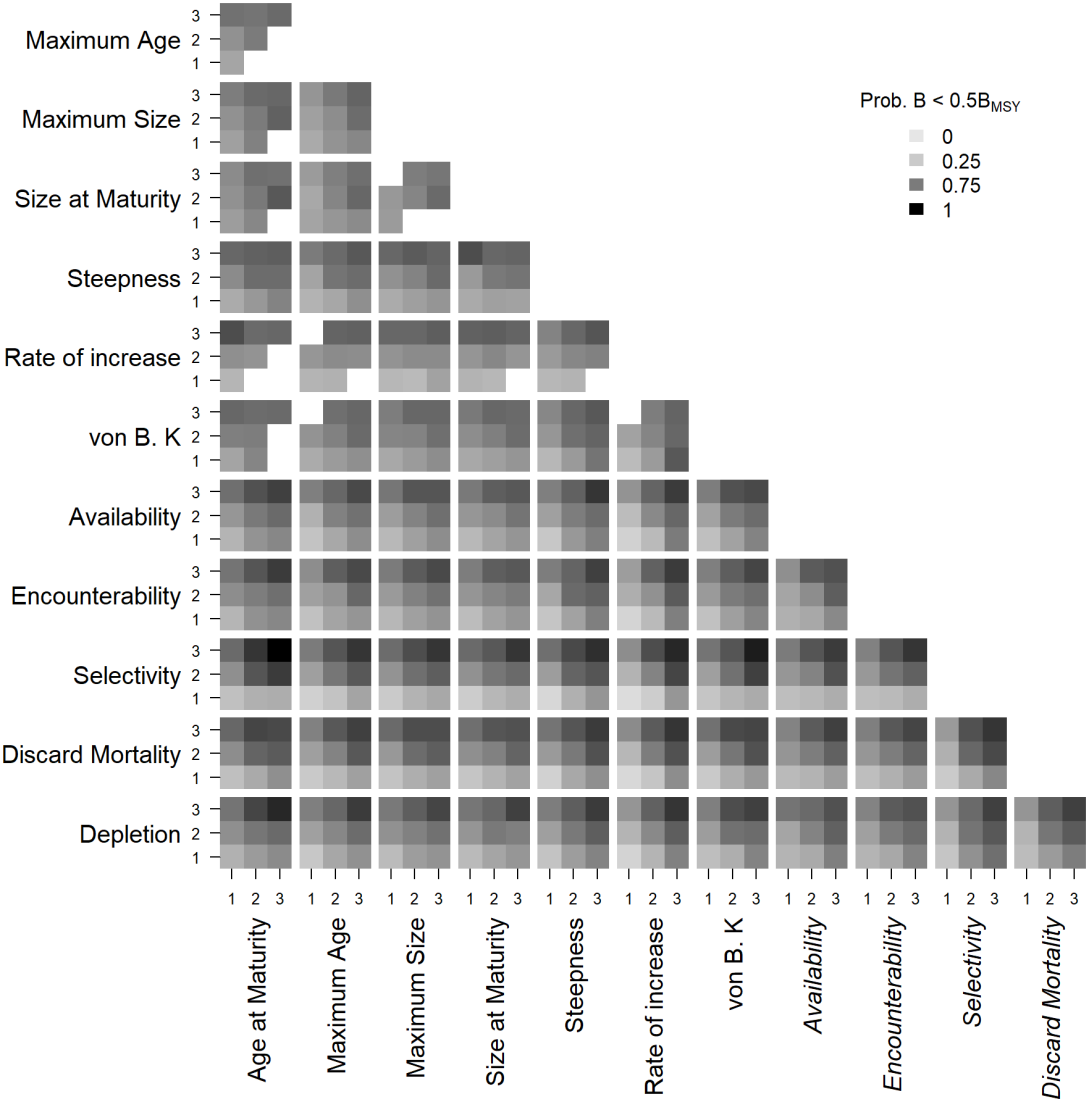
Interaction plot of the productivity and susceptibility of the additive extended PSA and risk. Interaction plot of the 7 productivity and 5 susceptibility (italics) attributes of the additive extended PSA and the probablity of spawning biomass (B) ending below 0.5 B_MSY_ at the end of the projection period with an exploitation rate of 0.4.

The relationship between the 3 risk categories of each of the 12 ePSA attributes and the quantitative estimate of risk is shown for the risk measure of B < 0.5B_MSY_ and an exploitation rate of 0.4 in Fig 7 (the other risk measures and exploitation rates are shown in S23–S30 Figs). The PSA assumes that risk increases with increasing risk scores of the individual attributes: i.e., from a minimum in the lowest risk categories (lower left corner of each plot in Fig 7) to a maximum in the highest risk categories (upper right corner of each plot in Fig 7). Furthermore, the PSA assumes that risk is an additive function of the individual productivity and susceptibility attributes: i.e., the risk in the lower right and upper left corner of each plot is assumed to be equivalent. In general, overall risk was lowest in the joint lowest risk categories of the individual attributes and increased to a maximum in the joint highest risk categories (Fig 7). However, there were several cases where this pattern was not observed. For example, the probability of biomass declining to low levels was highest when the Rate of Increase attribute was in the highest risk category and the Age of Maturity in the lowest and declined as the risk category for the Age of Maturity increased (Fig 7). A similar pattern was observed for the Steepness and Size of Maturity attributes, where risk declined as the risk category for Size of Maturity increased. The strong influence of the Selectivity attribute is clear in these results, which demonstrates that the probability of stock declining to low levels is relatively low when the Selectivity attribute is in the lowest risk category irrespective of the rating of the other 11 attributes (Fig 7).

These results demonstrate that there is a complex non-linear relationship between the individual productivity and susceptibility attributes and their relationship with overall risk. Furthermore, they demonstrate that certain combinations of risk scores for the productivity and susceptibility attributes are implausible. A clear example is the relationship between Size of Maturity and Maximum Size, where it was not possible to generate simulations where Size of Maturity is in the highest risk category and the Maximum Size in the lowest. Similarly, when Size of Maturity was rated in lowest risk category, it was not possible to generate life histories where Maximum Size was rated as medium or high risk.

**Fig 8:**
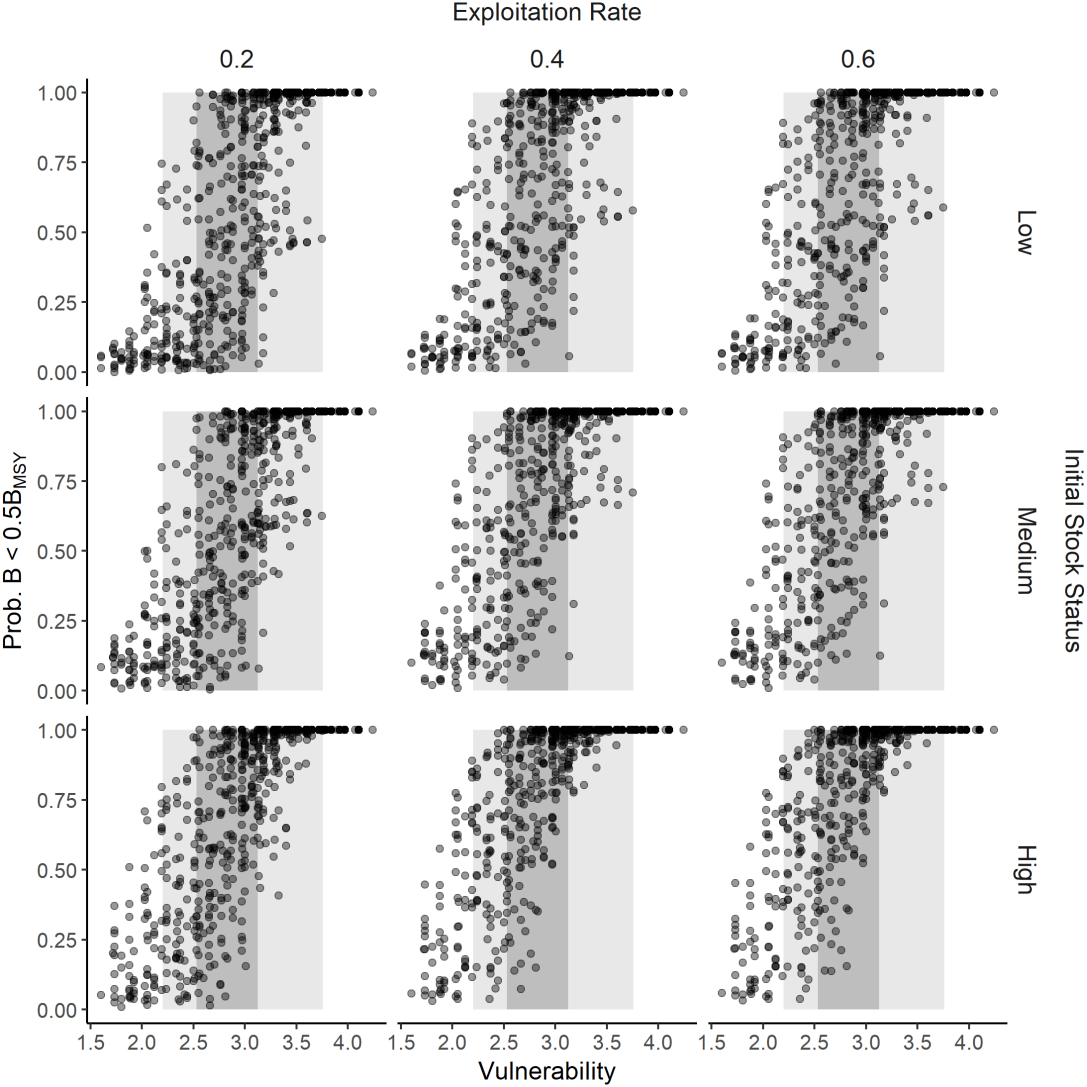
Scatterplots of PSA vulnerability scores and quantitative measure of risk for the standard PSA. Scatterplots showing PSA vulnerability scores (x-axis) and the probability of biomass being below 0.5*B*_MSY_ (y-axis) for the standard PSA (sPSA) using the additive method for calculating overall susceptibility score, for low, medium, and high initial stock size (rows) and low, medium, and high exploitation rate (columns). The gray shaded regions represent the 5^th^ and 95^th^ (light gray) and 25^th^ and 75^th^ (dark gray) percentiles of applications of the sPSA [17] and show that the scores for most applications fall within the mid-range values of the vulnerability score.

**Fig 9:**
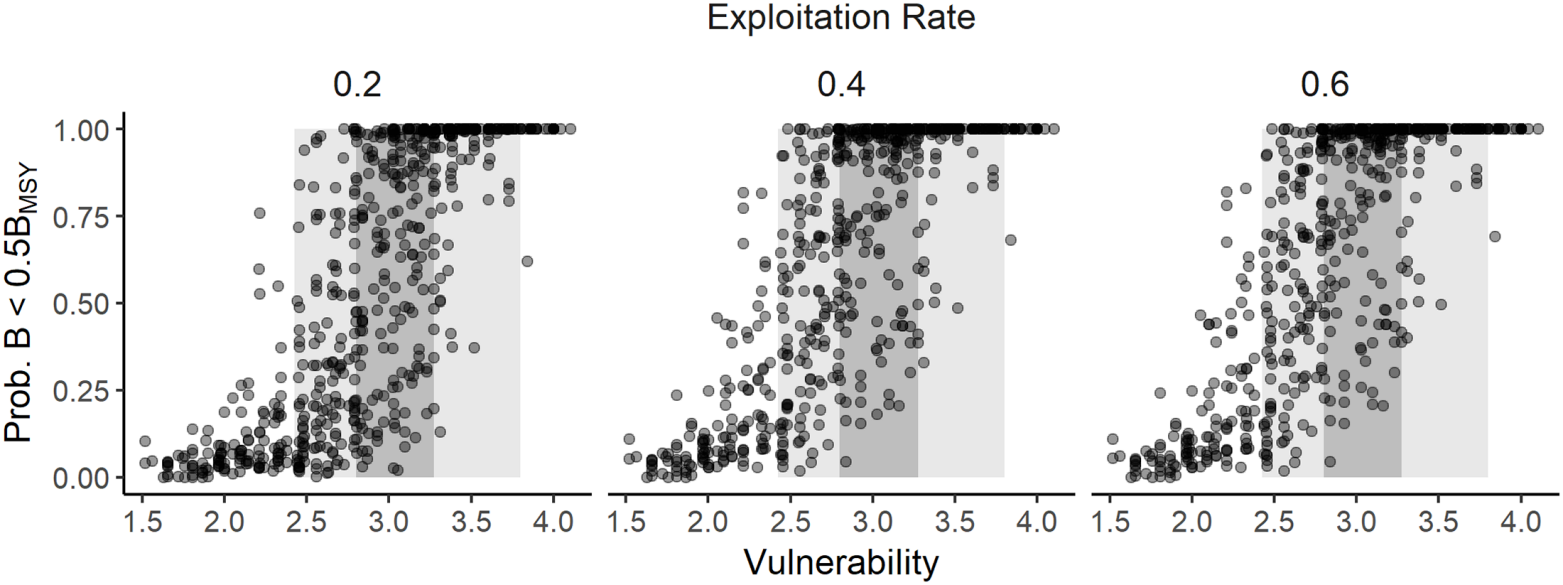
Scatterplots of PSA vulnerability scores and quantitative measure of risk for the extended PSA. Scatterplots showing PSA vulnerability scores (x-axis) and the probability of biomass being below 0.5*B*_MSY_ (y-axis) for the extended PSA (ePSA) using the additive method for calculating overall susceptibility score, for low, medium, and high exploitation rate (columns). The gray shaded regions represent the 5^th^ and 95^th^ (light gray) and 25^th^ and 75^th^ (dark gray) percentiles of applications of the ePSA [20] and show that the scores for most applications fall within the mid-range values of the vulnerability score.

Applications that use the PSA to evaluate a range of species and rank them according to risk assume that the PSA vulnerability score is a reliable indicator of biological risk. The results from this study demonstrate that, in general, the lowest and highest vulnerability scores correlate with a low and high biological risk respectively. However, while the risk generally increased with increasing vulnerability score, there was high variability among the individual simulations, particularly for vulnerability scores between 2.5 and 3.5 where the probability of B < 0.5B_MSY_ was found to range from 0 to 1 (Figs 8 and 9 for additive sPSA and ePSA respectively). This finding is particularly important as both the theory (Fig 1) and applications of the PSA (shaded regions in Figs 8 and 9) reveal that most stocks evaluated with the PSA result in mid-range vulnerability scores, where the vulnerability score is a very poor predictor of risk. A similar pattern was observed for the two alternative risk metrics (*B* < 0.2*B*_*0*_ and *B* < 0.1*B*_*0*_) and multiplicative method for calculating susceptibility score (S31–S40 Figs).

**Fig 10:**
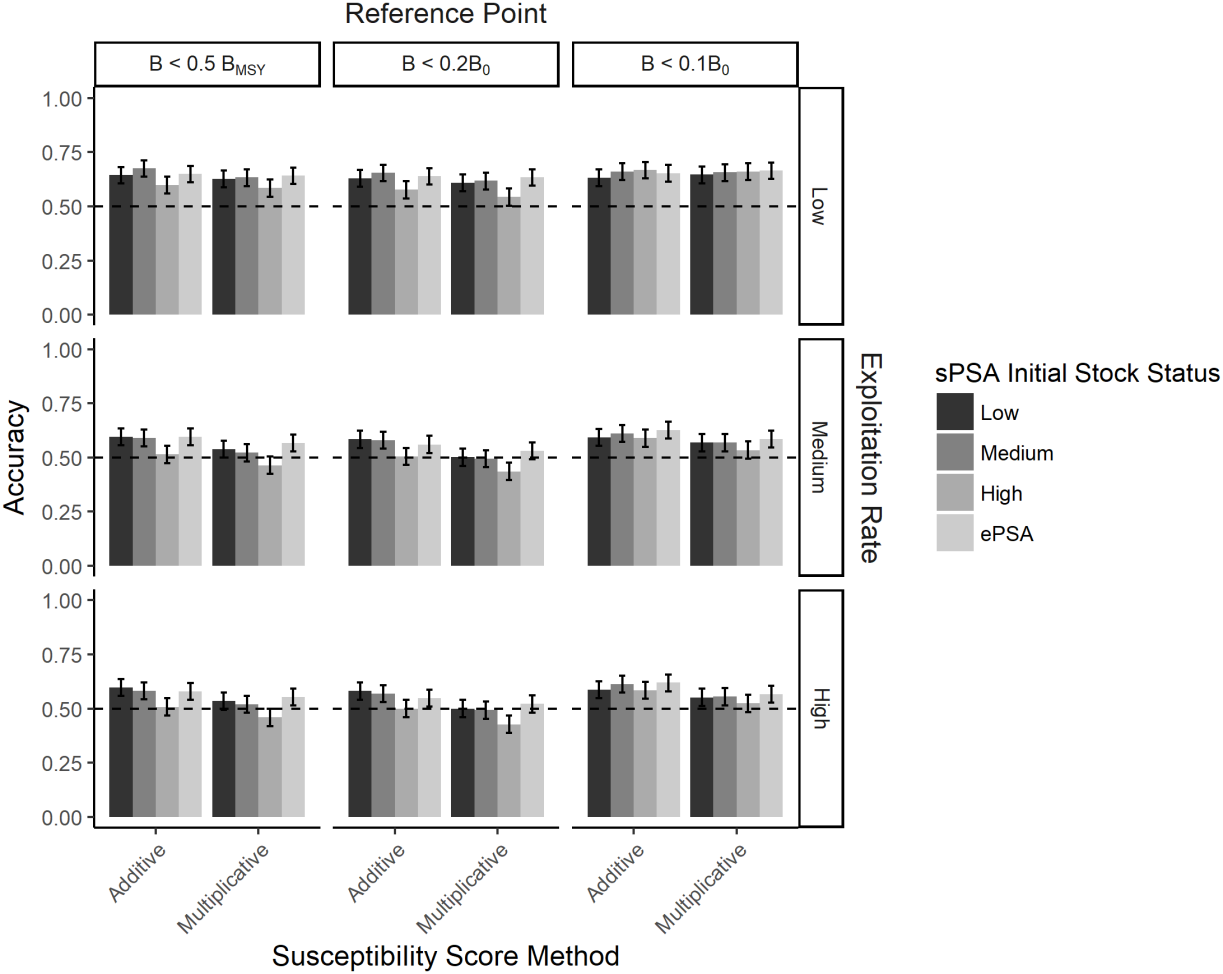
The expected accuracy of the standard PSA for different measures of risk and exploitation rates. The expected accuracy (with 95% confidence intervals) of the standard PSA (sPSA) with low, medium and high initial stock status and the extended PSA (ePSA; lightest gray) for three quantitative measures of risk (columns) and three future exploitation rates (rows). The results are shown for both the additive and multiplicative method for calculating the overall susceptibility score (x-axis).

The sPSA, under conditions of low future exploitation rate, medium initial stock status, additive method to calculate susceptibility score, and a biological reference point of B < 0.5B_MSY_, had the highest accuracy (67%) in terms of correctly assigning the risk fishery rating of a fishery (Fig 10). Prediction error rate of the sPSA tended to increase when the exploitation rate increased, or the multiplicative method was used to calculate the susceptibility score (Fig 10). In the worst case, under conditions of medium or high future exploitation rate, medium or high initial stock size, and using the multiplicative method, the classification success rate of the PSA was <50%, indicating that in more than half of these scenarios the PSA incorrectly assigned the risk rating (Fig 10). This was particularly the case for the Medium risk category, where the average true positive rate was 22% (range 16–33%).

The highest classification success rate of the ePSA was similar to that of the sPSA (66%), in the scenarios where future exploitation rate was low, the biological risk reference point of B < 0.1B_0_ was considered, and either the multiplicative or additive methods were used (Fig 10). Like the sPSA, accuracy declined as exploitation rate increased, with the worst performance occurring under conditions of medium or high exploitation rate and the multiplicative method of calculating susceptibility score (classification success rate of 53 and 52% respectively; Fig 10).

## Discussion

At the extreme ranges (V < 2 and V > 3.5) the PSA vulnerability score, for all the variants examined in this study, correlates reasonably well with risk of over-exploitation, although arguably this may be anticipated in the absence of a formal scoring system. More significant is the high uncertainty in risk for the intermediate vulnerability values where most fisheries are scored. For these mid-range values, the vulnerability score appears unrelated to risk, and fisheries ranked by these scores are unlikely to be ordered correctly with respect to risk of over-exploitation. Calculating a single vulnerability score is an attractive way to summarize the complex processes involved in determining the inherent risk of a fishery to over-exploitation. Our results suggest, however, that due to the high variability in relationship between risk and the vulnerability score this approach is overly-simplistic.

Given that the PSA approach has been used to evaluate risk of over 1,000 stocks, including fish, sharks and rays, marine mammals, sea turtles, and seabirds (Table 1), these results have sobering implications for the use of qualitative risk-based frameworks for evaluating and ranking risk of over-exploitation of target and by-catch species. The results of this study suggest that, under the most favorable conditions (one of the 72 simulated conditions), the expected success rate of the PSA in categorizing fisheries as low, medium, or high risk is about 66%. Under other conditions, for example, high exploitation rate and low initial stock size, the performance declines considerably, to success rates of less than 50% (worse than a coin toss) for the sPSA and ~55% for the ePSA.

Qualitative scoring systems are often considered useful tools for evaluating vulnerability of marine stocks to fishing pressure, particularly for prioritizing research and management of fisheries according to risk of over-exploitation [47,57,58]. However, these frameworks are often subjective and not reproducible. Reproducibility is a key tenet of the scientific method and crucial for theoretical testing of proposed methods in a simulation framework [59]. The difficulty in observing biological processes means that expert opinion is required in many areas of research in biology and ecology, and fisheries are no exception. It is important that the heuristic process of consolidating expert knowledge to inform modeling or analysis is well documented, reproducible and testable [60]. Qualitative scoring systems that are proposed for evaluating fisheries and providing advice to management and research should follow these principles so that their predictions and recommendations can be evaluated against empirical evidence and also theoretically by simulation.

**Fig 11:**
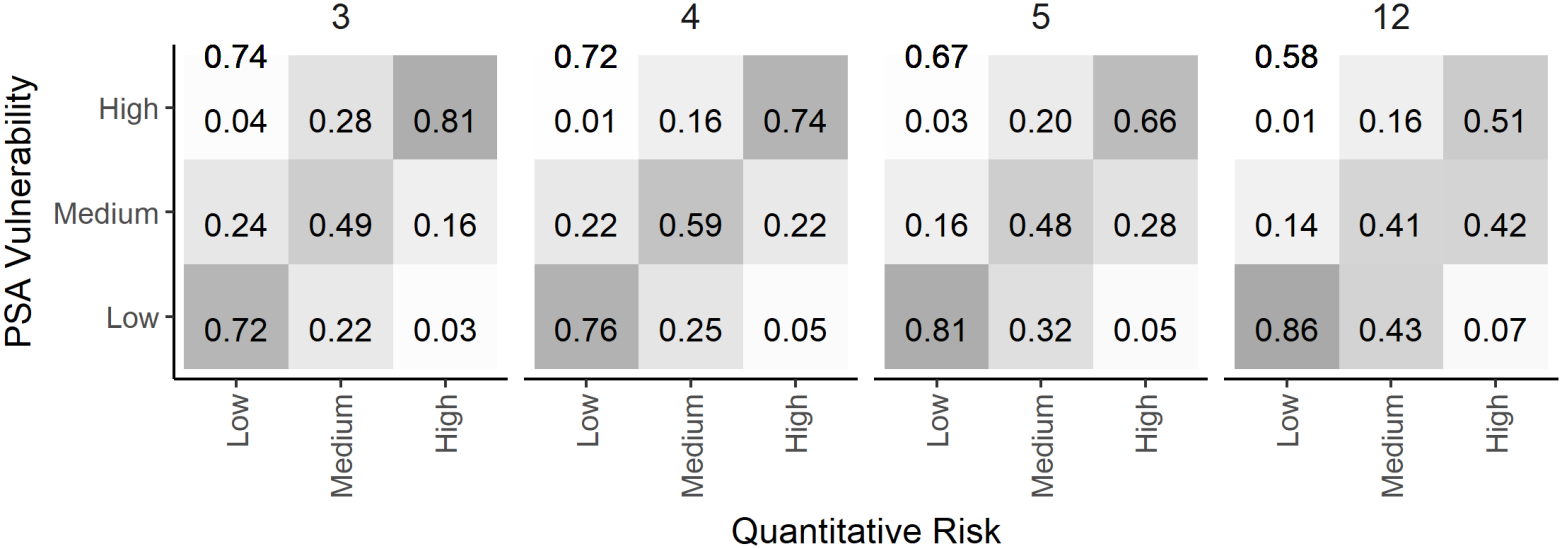
A comparisoin of the PSA risk ratings and the quantitative measure of risk calculated from different numbers of risk attributes. Comparison of the PSA risk ratings and the quantitative measure of risk for 3 (rate of increase, selectivity, and discard mortality), 4 (previous plus steepness), 5 (previous plus encounterablity), and all 12 productivity and susceptibility attributes of the ePSA with high exploitation rate and B < 0.5 B_MSY_ reference point. The values in each cell represent the fraction that the PSA assigned each risk category (y-axis) compared to the quantitative evaluation of risk (x-axis). Each column sums to one, and the values on the antidiagonal represent the true prediction rates for each risk category. The overall true prediction rate is shown in the top left corner of each plot.

The results of this simulation study reveal that the productivity and susceptibility attributes make an unequal contribution to risk. For example, the size of capture relative to the size of maturity (Selectivity) was the most significant predictor of risk in all analyses examined here, while the Size of Maturity attribute contributed very little. This suggests that the scoring system may be over-parameterized where the addition of irrelevant or correlated attributes may degrade the predictive capacity of the approach. We examined this possibility by recalculating the PSA vulnerability score with the three, four, and five most important predictors and comparing the prediction error rate with that of all 12 attributes in the ePSA (Fig 11). This analysis revealed that the overall prediction error rate increases as more attributes were added to the scoring system, with the highest prediction accuracy occurring when only one productivity and two susceptibility attributes were used (Rate of Increase, Selectivity, and Discard Mortality respectively) and the lowest accuracy when all 12 attributes were used (Fig 11). This preliminary analysis, which only included the assumption of high exploitation rates, suggests that for the poorly performing Medium risk category the optimum model complexity is 4 attributes (Rate of Increase, Selectivity, Discard Mortality, and Encounterability), which leads to an increase in true prediction rate from 41% with all 12 attributes to 59%. However, more extensive testing over a wider range of conditions should be carried out before establishing a modified version of the scoring system with fewer or alternative productivity and susceptibility attributes.

The inability to map certain PSA attributes to operating model parameters (e.g., trophic level), our quantitative interpretation of some attributes, or our definitions of risk, may be perceived as important limitations of this research—that by omitting potentially critical features of PSA this was an unfair test of the approach. Conversely, the fact that we assumed biological parameters and fishing characteristics were known perfectly when calculating the PSA risk scores (i.e., there was no observation error in assigning the risk scores for each productivity and susceptibility attribute) may be considered to bias the results towards an overly optimistic evaluation of the performance of PSA. Such criticism is welcomed since it goes to the philosophical subject of this paper. If there are grounds to criticize the approach applied here, what are the objective substance of these criticisms? Quantitatively, how important are these omissions? Answers to these questions must lie in either more thorough theoretical testing similar to this research or demonstrated empirically, for example meta-analyses of historical stock performance in relation to the proposed attributes (e.g., trophic level).

Despite the difficulties in interpreting some of the PSA attributes, we were able to construct operating models based on the individual scores of the productivity and susceptibility attributes. We argue that the information required to complete a qualitative scoring system such as the PSA is comparable to that needed to populate an operating model. An operating model, however, offers several advantages over qualitative approaches. It provides a transparent representation of the current state of knowledge of the fishery, for both the population and the fishing dynamics, and can incorporate uncertainty in critical aspects of the system. Importantly, this allows analysts to evaluate alternative hypotheses about the current or future population dynamics and evaluate the impact of the uncertainty on the risk to the population. We recommend developing operating models for exploited stocks and using conventional simulation frameworks to evaluate risk, determine critical uncertainties, and inform research and management of the fishery. A key advantage of the operating model approach it can be easily incorporated into a management strategy evaluation (MSE) framework which can be used to evaluate alternative management strategies and identity those methods that are robust to uncertainty and likely to meet the management objectives of the fishery.

Fisheries managers, environmental NGOs, and other stakeholders face an enormous challenge of quickly and efficiently evaluating risk and prioritizing species for management and further research and rely on the science community to develop tools to tackle these problems. The PSA and other similar qualitative risk assessment frameworks were initially developed to address this challenge and undoubtedly have been valuable in providing a framework for identifying species most at risk of over-exploitation. Recent developments including the affordability of high power computing, open-source software, and online data-bases have substantially lowered the barriers to developing operating models and evaluating risk with simulation modelling. Rather than relying on subjective scoring systems, these resources and technologies should be harnessed to develop quantitative and objective risk assessment tools that provide robust and reliable advice to support sustainable management of marine resources.

## Supporting information

S1 Fig The relationship between the productivity and susceptibility scores and the probability of B < 0.5B_MSY_ as predicted by the PSA and the observed pattern for the additive standard PSA (sPSA). Risk in each plot has been standardized to a minimum and maximum value of 0 and 1.
(PNG)

S2 Fig The relationship between the productivity and susceptibility scores and the probability of B < 0.5B_MSY_ as predicted by the PSA and the observed pattern for the multiplicative standard PSA (sPSA). Risk in each plot has been standardized to a minimum and maximum value of 0 and 1.
(PNG)

S3 Fig The relationship between the productivity and susceptibility scores and the probability of B < 0.2B_0_ as predicted by the PSA and the observed pattern for the additive standard PSA (sPSA). Risk in each plot has been standardized to a minimum and maximum value of 0 and 1.
(PNG)

S4 Fig The relationship between the productivity and susceptibility scores and the probability of B < 0.2B_0_ as predicted by the PSA and the observed pattern for the multiplicative standard PSA (sPSA). Risk in each plot has been standardized to a minimum and maximum value of 0 and 1.
(PNG)

S5 Fig The relationship between the productivity and susceptibility scores and the probability of B < 0.1B_0_ as predicted by the PSA and the observed pattern for the additive standard PSA (sPSA). Risk in each plot has been standardized to a minimum and maximum value of 0 and 1.
(PNG)

S6 Fig The relationship between the productivity and susceptibility scores and the probability of B < 0.1B_0_ as predicted by the PSA and the observed pattern for the multiplicative standard PSA (sPSA). Risk in each plot has been standardized to a minimum and maximum value of 0 and 1.
(PNG)

S7 Fig The relationship between the productivity and susceptibility scores and the probability of B < 0.5B_MSY_ as predicted by the PSA and the observed pattern for the additive extended PSA (ePSA). Risk in each plot has been standardized to a minimum and maximum value of 0 and 1.
(PNG)

S8 Fig The relationship between the productivity and susceptibility scores and the probability of B < 0.5B_MSY_ as predicted by the PSA and the observed pattern for the multiplicative extended PSA (ePSA). Risk in each plot has been standardized to a minimum and maximum value of 0 and 1.
(PNG)

S9 Fig The relationship between the productivity and susceptibility scores and the probability of B < 0.2B_0_ as predicted by the PSA and the observed pattern for the additive extended PSA (ePSA). Risk in each plot has been standardized to a minimum and maximum value of 0 and 1.
(PNG)

S10 Fig The relationship between the productivity and susceptibility scores and the probability of B < 0.2B_0_ as predicted by the PSA and the observed pattern for the multiplicative extended PSA (ePSA). Risk in each plot has been standardized to a minimum and maximum value of 0 and 1.
(PNG)

S11 Fig The relationship between the productivity and susceptibility scores and the probability of B < 0.1B_0_ as predicted by the PSA and the observed pattern for the additive extended PSA (ePSA). Risk in each plot has been standardized to a minimum and maximum value of 0 and 1.
(PNG)

S12 Fig The relationship between the productivity and susceptibility scores and the probability of B < 0.1B_0_ as predicted by the PSA and the observed pattern for the multiplicative extended PSA (ePSA). Risk in each plot has been standardized to a minimum and maximum value of 0 and 1.
(PNG)

S13 Fig The relative contribution of the 7 productivity and 5 susceptibility (italics) attributes of the additive extended PSA in explaining the variation of spawning biomass (B) at the end of the projection period being below 0.2B_0_ under conditions of low, medium, and high exploitation rates. (PNG)

S14 Fig The relative contribution of the 7 productivity and 5 susceptibility (italics) attributes of the additive extended PSA in explaining the variation of spawning biomass (B) at the end of the projection period being below 0.1B_0_ under conditions of low, medium, and high exploitation rates. (PNG)

S15 Fig The relative contribution of the 7 productivity and 5 susceptibility (italics) attributes of the multiplicative extended PSA in explaining the variation of spawning biomass (B) at the end of the projection period being below 0.5B_MSY_ under conditions of low, medium, and high exploitation rates. (PNG)

S16 Fig The relative contribution of the 7 productivity and 5 susceptibility (italics) attributes of the multiplicative extended PSA in explaining the variation of spawning biomass (B) at the end of the projection period being below 0.2B_0_ under conditions of low, medium, and high exploitation rates. (PNG)

S17 Fig The relative contribution of the 7 productivity and 5 susceptibility (italics) attributes of the multiplicative extended PSA in explaining the variation of spawning biomass (B) at the end of the projection period being below 0.1B_0_ under conditions of low, medium, and high exploitation rates. (PNG)

S18 Fig The relative contribution of the 5 productivity and 4 susceptibility (italics) attributes of the additive standard PSA in explaining the variation of spawning biomass (B) at the end of the projection period being below 0.2B_0_ under conditions of low, medium and high initial stock size (rows) and low, medium, and high exploitation rates (columns). (PNG)

S19 Fig The relative contribution of the 5 productivity and 4 susceptibility (italics) attributes of the additive standard PSA in explaining the variation of spawning biomass (B) at the end of the projection period being below 0.1B_0_ under conditions of low, medium and high initial stock size (rows) and low, medium, and high exploitation rates (columns). (PNG)

S20 Fig The relative contribution of the 5 productivity and 4 susceptibility (italics) attributes of the multiplicative standard PSA in explaining the variation of spawning biomass (B) at the end of the projection period being below 0.5B_MSY_ under conditions of low, medium and high initial stock size (rows) and low, medium, and high exploitation rates (columns). (PNG)

S21 Fig The relative contribution of the 5 productivity and 4 susceptibility (italics) attributes of the multiplicative standard PSA in explaining the variation of spawning biomass (B) at the end of the projection period being below 0.2B_0_ under conditions of low, medium and high initial stock size (rows) and low, medium, and high exploitation rates (columns). (PNG)

S22 Fig The relative contribution of the 5 productivity and 4 susceptibility (italics) attributes of the multiplicative standard PSA in explaining the variation of spawning biomass (B) at the end of the projection period being below 0.1B_0_ under conditions of low, medium and high initial stock size (rows) and low, medium, and high exploitation rates (columns). (PNG)

S23 Fig Interaction plot of the 7 productivity and 5 susceptibility (italics) attributes of the additive extended PSA and the probablity of spawning biomass (B) ending below 0.5 B_MSY_ at the end of the projection period with an exploitation rate of 0.2. (PNG)

S24 Fig Interaction plot of the 7 productivity and 5 susceptibility (italics) attributes of the additive extended PSA and the probablity of spawning biomass (B) ending below 0.5 B_MSY_ at the end of the projection period with an exploitation rate of 0.6. (PNG)

S25 Fig Interaction plot of the 7 productivity and 5 susceptibility (italics) attributes of the additive extended PSA and the probablity of spawning biomass (B) ending below 0.2 B_0_ at the end of the projection period with an exploitation rate of 0.2. (PNG)

S26 Fig Interaction plot of the 7 productivity and 5 susceptibility (italics) attributes of the additive extended PSA and the probablity of spawning biomass (B) ending below 0.2 B_0_ at the end of the projection period with an exploitation rate of 0.4. (PNG)

S27 Fig Interaction plot of the 7 productivity and 5 susceptibility (italics) attributes of the additive extended PSA and the probablity of spawning biomass (B) ending below 0.2 B_0_ at the end of the projection period with an exploitation rate of 0.6. (PNG)

S28 Fig Interaction plot of the 7 productivity and 5 susceptibility (italics) attributes of the additive extended PSA and the probablity of spawning biomass (B) ending below 0.1 B_0_ at the end of the projection period with an exploitation rate of 0.2. (PNG)

S29 Fig Interaction plot of the 7 productivity and 5 susceptibility (italics) attributes of the additive extended PSA and the probablity of spawning biomass (B) ending below 0.1 B_0_ at the end of the projection period with an exploitation rate of 0.4. (PNG)

S30 Fig Interaction plot of the 7 productivity and 5 susceptibility (italics) attributes of the additive extended PSA and the probablity of spawning biomass (B) ending below 0.1 B_0_ at the end of the projection period with an exploitation rate of 0.6. (PNG)

S31 Fig Scatterplots showing PSA Vulnerability scores (x-axis) and the probability of biomass being below 0.2*B*_0_ (y-axis) for the standard PSA (sPSA) using the additive method for calculating overall susceptibility score, for low, medium, and high initial stock size (rows) and low, medium, and high exploitation rate (columns). The gray shaded regions represent the 5^th^ and 95^th^ (light gray) and 25^th^ and 75^th^ (dark gray) percentiles of applications of the sPSA [17] and show that the scores for most applications fall within the mid-range values of the vulnerability score.
(PNG)

S32 Fig Scatterplots showing PSA Vulnerability scores (x-axis) and the probability of biomass being below 0.1*B*_0_ (y-axis) for the standard PSA (sPSA) using the additive method for calculating overall susceptibility score, for low, medium, and high initial stock size (rows) and low, medium, and high exploitation rate (columns). The gray shaded regions represent the 5^th^ and 95^th^ (light gray) and 25^th^ and 75^th^ (dark gray) percentiles of applications of the sPSA [17]and show that the scores for most applications fall within the mid-range values of the vulnerability score.
(PNG)

S33 Fig Scatterplots showing PSA Vulnerability scores (x-axis) and the probability of biomass being below 0.5*B*_MSY_ (y-axis) for the standard PSA (sPSA) using the multiplicative method for calculating overall susceptibility score, for low, medium, and high initial stock size (rows) and low, medium, and high exploitation rate (columns). The gray shaded regions represent the 5^th^ and 95^th^ (light gray) and 25^th^ and 75^th^ (dark gray) percentiles of applications of the sPSA [17] and show that the scores for most applications fall within the mid-range values of the vulnerability score.
(PNG)

S34 Fig Scatterplots showing PSA Vulnerability scores (x-axis) and the probability of biomass being below 0.2*B*_0_ (y-axis) for the standard PSA (sPSA) using the multiplicative method for calculating overall susceptibility score, for low, medium, and high initial stock size (rows) and low, medium, and high exploitation rate (columns). The gray shaded regions represent the 5^th^ and 95^th^ (light gray) and 25^th^ and 75^th^ (dark gray) percentiles of applications of the sPSA [17] and show that the scores for most applications fall within the mid-range values of the vulnerability score.
(PNG)

S35 Fig Scatterplots showing PSA Vulnerability scores (x-axis) and the probability of biomass being below 0.1*B*_0_ (y-axis) for the standard PSA (sPSA) using the multiplicative method for calculating overall susceptibility score, for low, medium, and high initial stock size (rows) and low, medium, and high exploitation rate (columns). The gray shaded regions represent the 5^th^ and 95^th^ (light gray) and 25^th^ and 75^th^ (dark gray) percentiles of applications of the sPSA [17] and show that the scores for most applications fall within the mid-range values of the vulnerability score.
(PNG)

S36 Fig Scatterplots showing PSA Vulnerability scores (x-axis) and the probability of biomass being below 0.2*B*_0_ (y-axis) for the extended PSA (ePSA) using the additive method for calculating overall susceptibility score, for low, medium, and high exploitation rate (columns). The gray shaded regions represent the 5^th^ and 95^th^ (light gray) and 25^th^ and 75^th^ (dark gray) percentiles of applications of the ePSA [20] and show that the scores for most applications fall within the mid-range values of the vulnerability score.
(PNG)

S37 Fig Scatterplots showing PSA Vulnerability scores (x-axis) and the probability of biomass being below 0.1*B*_0_ (y-axis) for the extended PSA (ePSA) using the additive method for calculating overall susceptibility score, for low, medium, and high exploitation rate (columns). The gray shaded regions represent the 5^th^ and 95^th^ (light gray) and 25^th^ and 75^th^ (dark gray) percentiles of applications of the ePSA [20] and show that the scores for most applications fall within the mid-range values of the vulnerability score.
(PNG)

S38 Fig Scatterplots showing PSA Vulnerability scores (x-axis) and the probability of biomass being below 0.5*B*_MSY_ (y-axis) for the extended PSA (ePSA) using the multiplicative method for calculating overall susceptibility score, for low, medium, and high exploitation rate (columns). The gray shaded regions represent the 5^th^ and 95^th^ (light gray) and 25^th^ and 75^th^ (dark gray) percentiles of applications of the ePSA [20] and show that the scores for most applications fall within the mid-range values of the vulnerability score.
(PNG)

S39 Fig Scatterplots showing PSA Vulnerability scores (x-axis) and the probability of biomass being below 0.2*B*_0_ (y-axis) for the extended PSA (ePSA) using the multiplicative method for calculating overall susceptibility score, for low, medium, and high exploitation rate (columns). The gray shaded regions represent the 5^th^ and 95^th^ (light gray) and 25^th^ and 75^th^ (dark gray) percentiles of applications of the ePSA [20] and show that the scores for most applications fall within the mid-range values of the vulnerability score.
(PNG)

S40 Fig Scatterplots showing PSA Vulnerability scores (x-axis) and the probability of biomass being below 0.1*B*_0_ (y-axis) for the extended PSA (ePSA) using the multiplicative method for calculating overall susceptibility score, for low, medium, and high exploitation rate (columns). The gray shaded regions represent the 5^th^ and 95^th^ (light gray) and 25^th^ and 75^th^ (dark gray) percentiles of applications of the ePSA [20] and show that the scores for most applications fall within the mid-range values of the vulnerability score.
(PNG)
