# Correction: A quantitative evaluation of a qualitative risk assessment framework: Examining the assumptions and predictions of the Productivity Susceptibility Analysis (PSA)

**DOI:** 10.1371/journal.pone.0206575

**Published:** 2018-10-25

**Authors:** The PLOS ONE Staff

There is an error in the penultimate sentence of the fourth paragraph of the methods. The correct sentence is: Sixty years was chosen as the upper bound for the maximum age (i.e., 25 < *A*_max_ < 60 years = low productivity) and a lower bound of 5 years (i.e., 5 < *A*_max_ < 10 years = high productivity; low risk), as this includes the lifespan of most marine fishes [35] (Table 2). The publisher apologizes for this error.

There is an error in the caption for Fig 11. Please see the complete, correct Fig 11 caption here.

**Fig 11:**
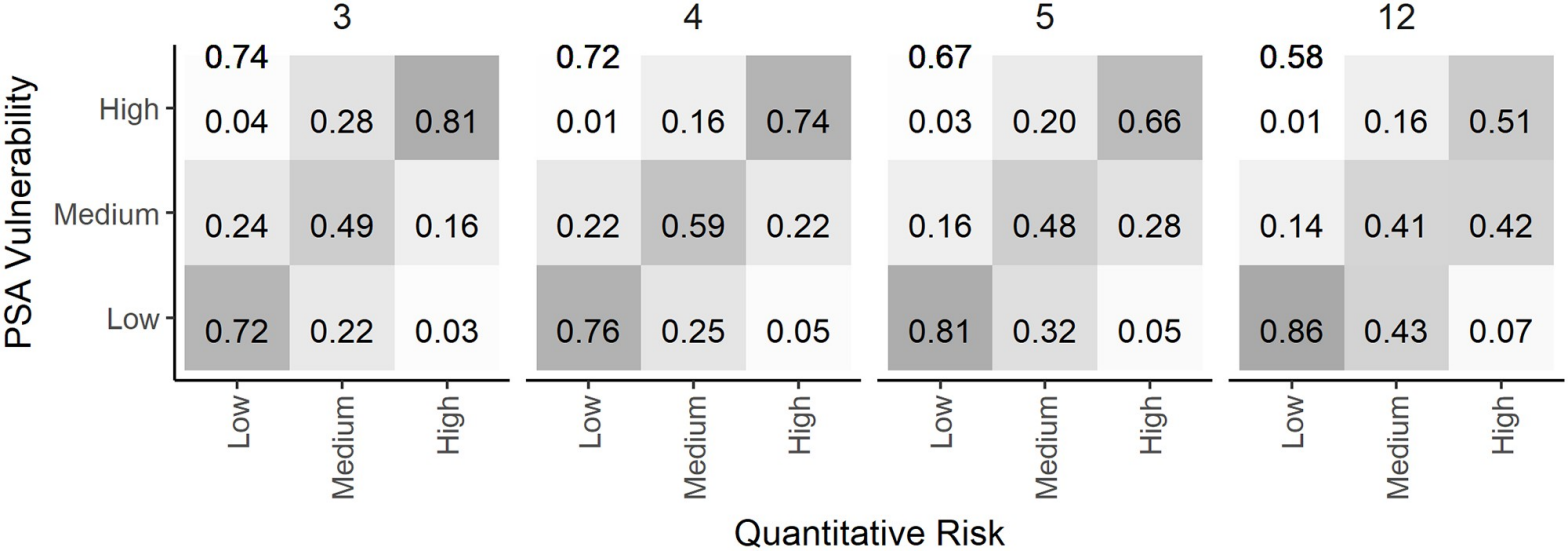
A comparison of the PSA risk ratings and the quantitative measure of risk calculated from different numbers of risk attributes. Comparison of the PSA risk ratings and the quantitative measure of risk for 3 (rate of increase, selectivity, and discard mortality), 4 (previous plus steepness), 5 (previous plus encounterablity), and all 12 productivity and susceptibility attributes of the ePSA with high exploitation rate and B < 0.5 B_MSY_ reference point. The values in each cell represent the fraction that the PSA assigned each risk category (y-axis) compared to the quantitative evaluation of risk (x-axis). Each column sums to one, and the values on the antidiagonal represent the true prediction rates for each risk category. The overall true prediction rate is shown in the top left corner of each plot. https://doi.org/10.1371/journal.pone.0198298.g011.
