# Supplementary figures and images for: A quantitative evaluation of a qualitative risk assessment framework: Examining the assumptions and predictions of the Productivity Susceptibility Analysis (PSA)
Source: PLoS One. 2018 Jun 1;13(6):e0198298. doi: 10.1371/journal.pone.0198298 (PMC5983465; doi:10.1371/journal.pone.0198298)

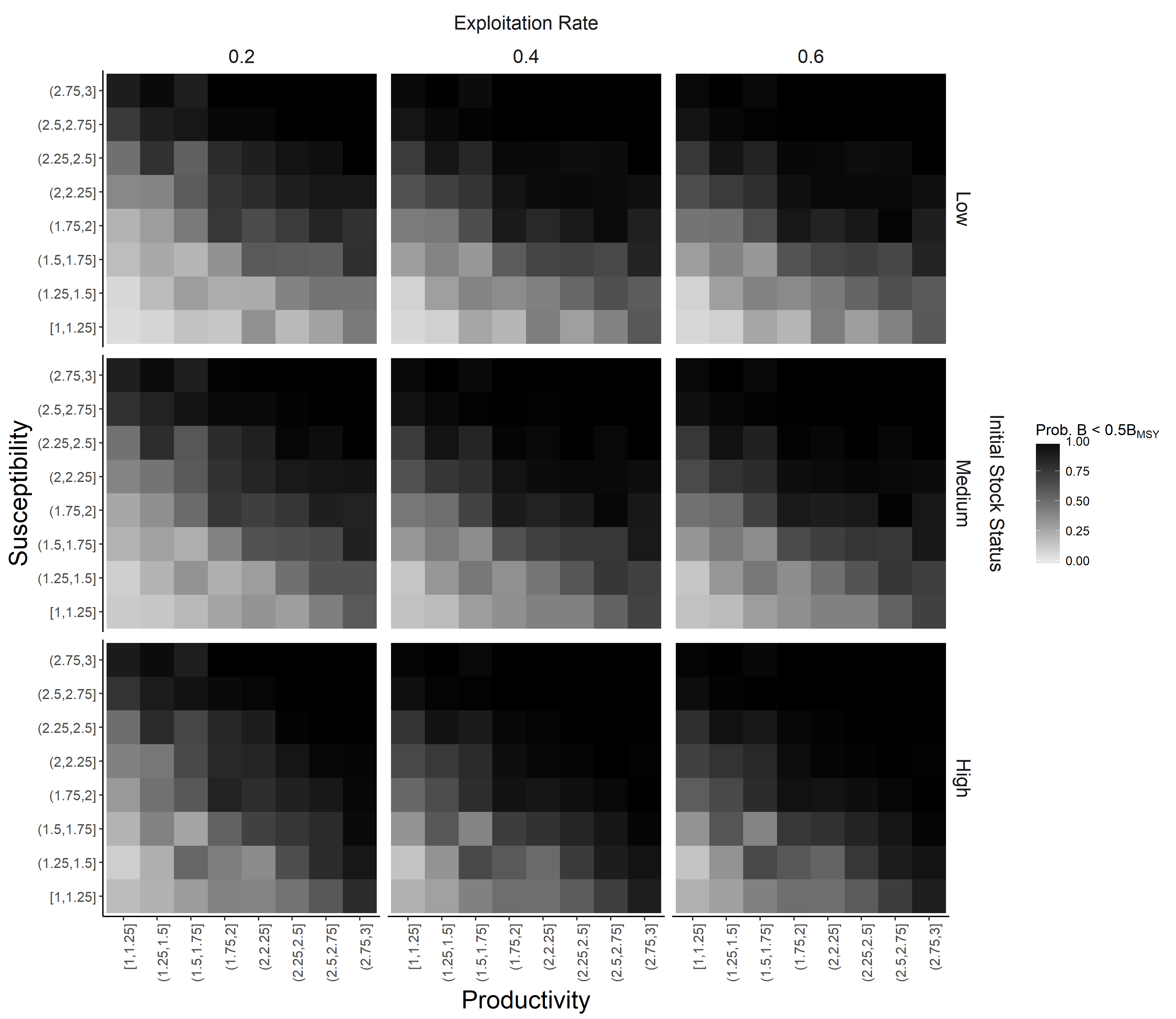

Supplement: S1 Fig — Risk in each plot has been standardized to a minimum and maximum value of 0 and 1. (PNG) [file pone.0198298.s001.png]

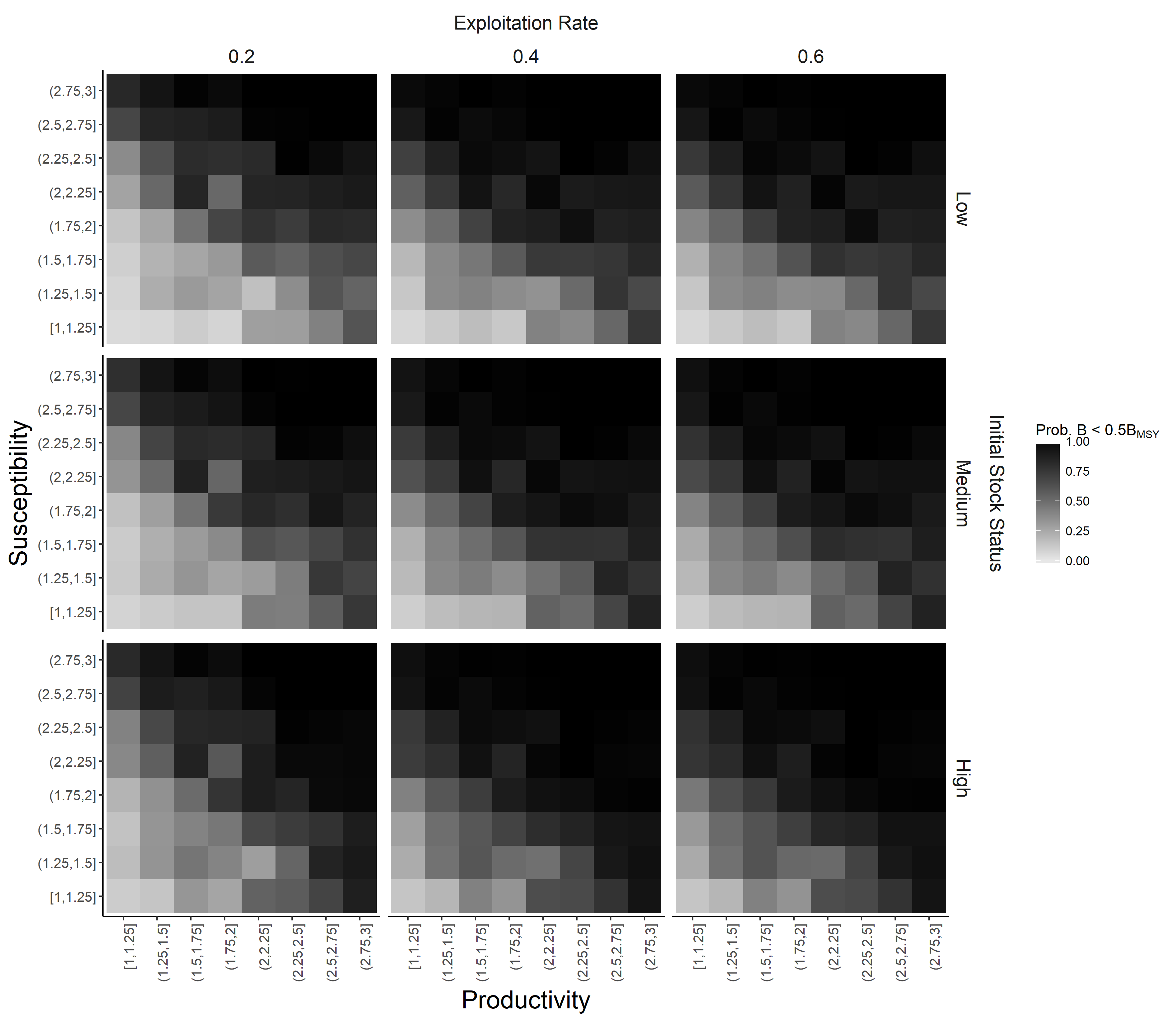

Supplement: S2 Fig — Risk in each plot has been standardized to a minimum and maximum value of 0 and 1. (PNG) [file pone.0198298.s002.png]

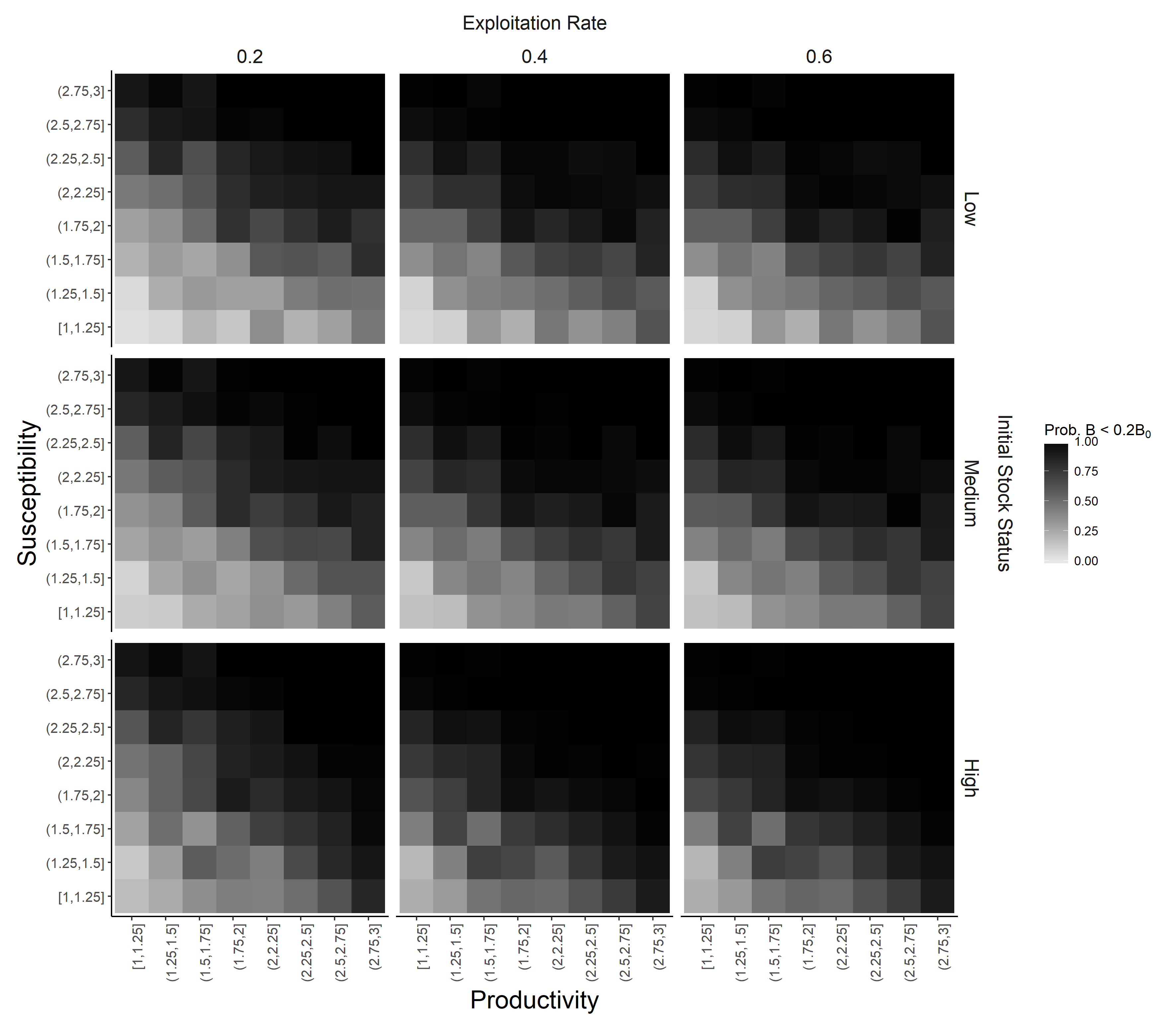

Supplement: S3 Fig — Risk in each plot has been standardized to a minimum and maximum value of 0 and 1. (PNG) [file pone.0198298.s003.png]

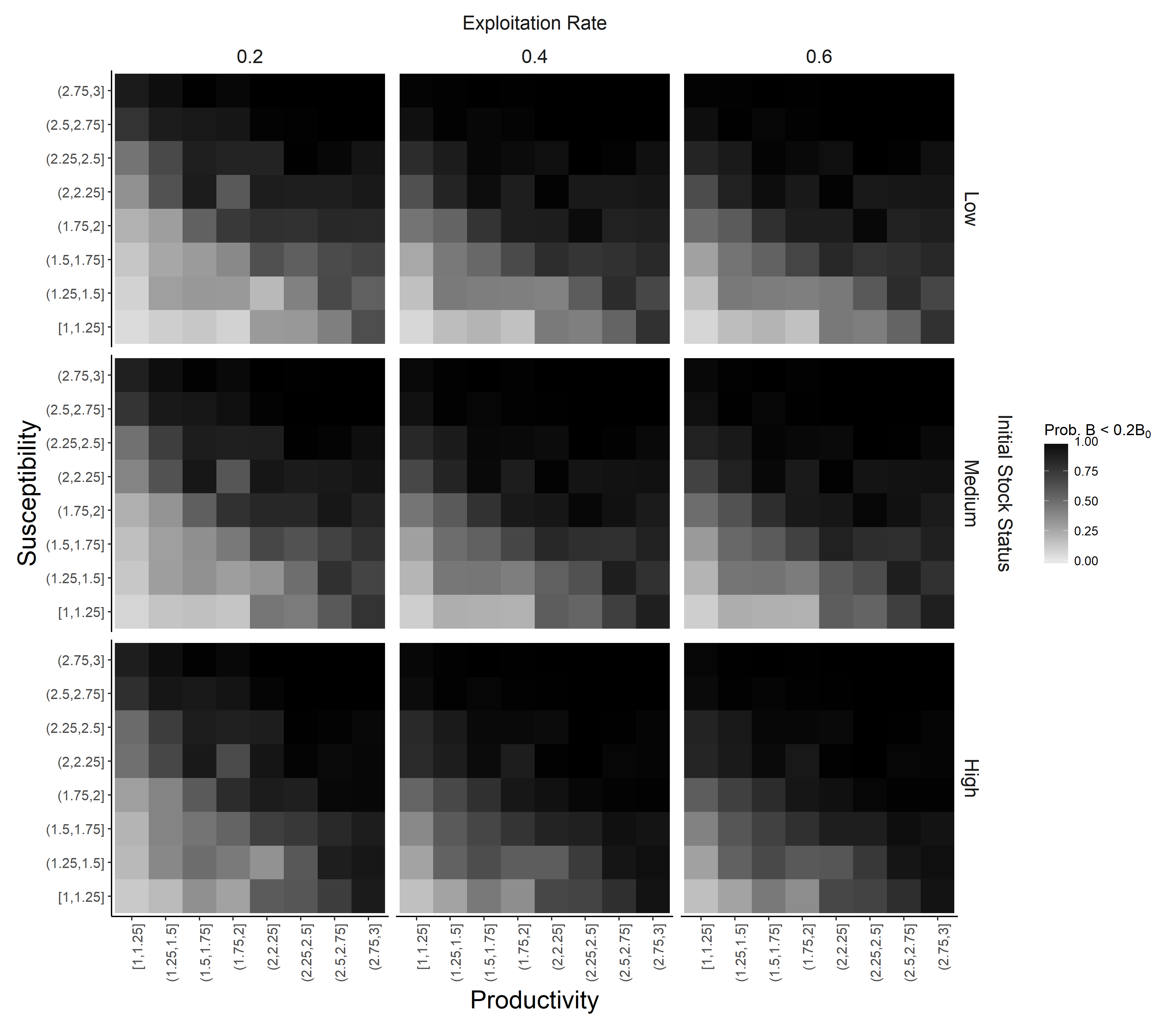

Supplement: S4 Fig — Risk in each plot has been standardized to a minimum and maximum value of 0 and 1. (PNG) [file pone.0198298.s004.png]

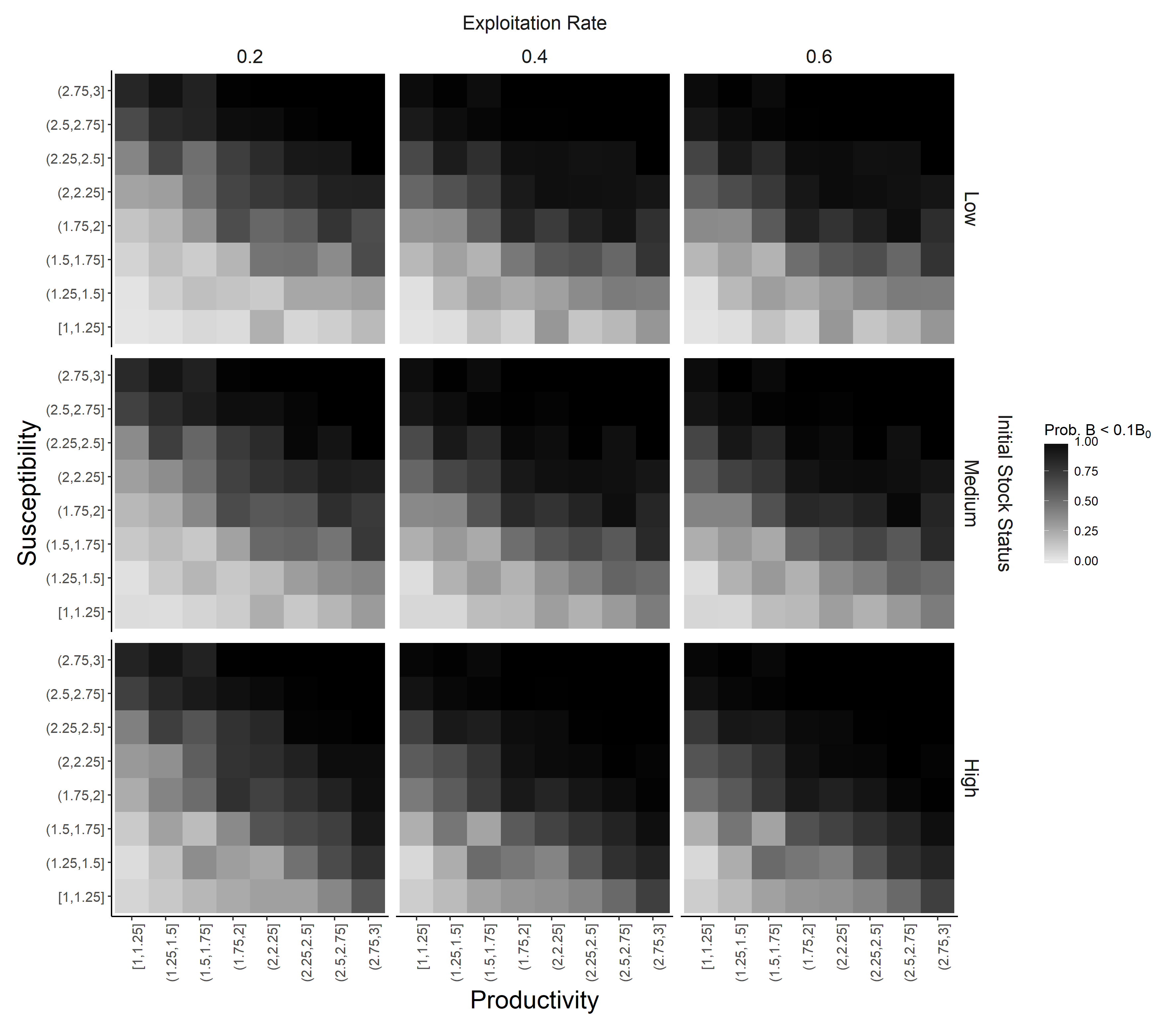

Supplement: S5 Fig — Risk in each plot has been standardized to a minimum and maximum value of 0 and 1. (PNG) [file pone.0198298.s005.png]

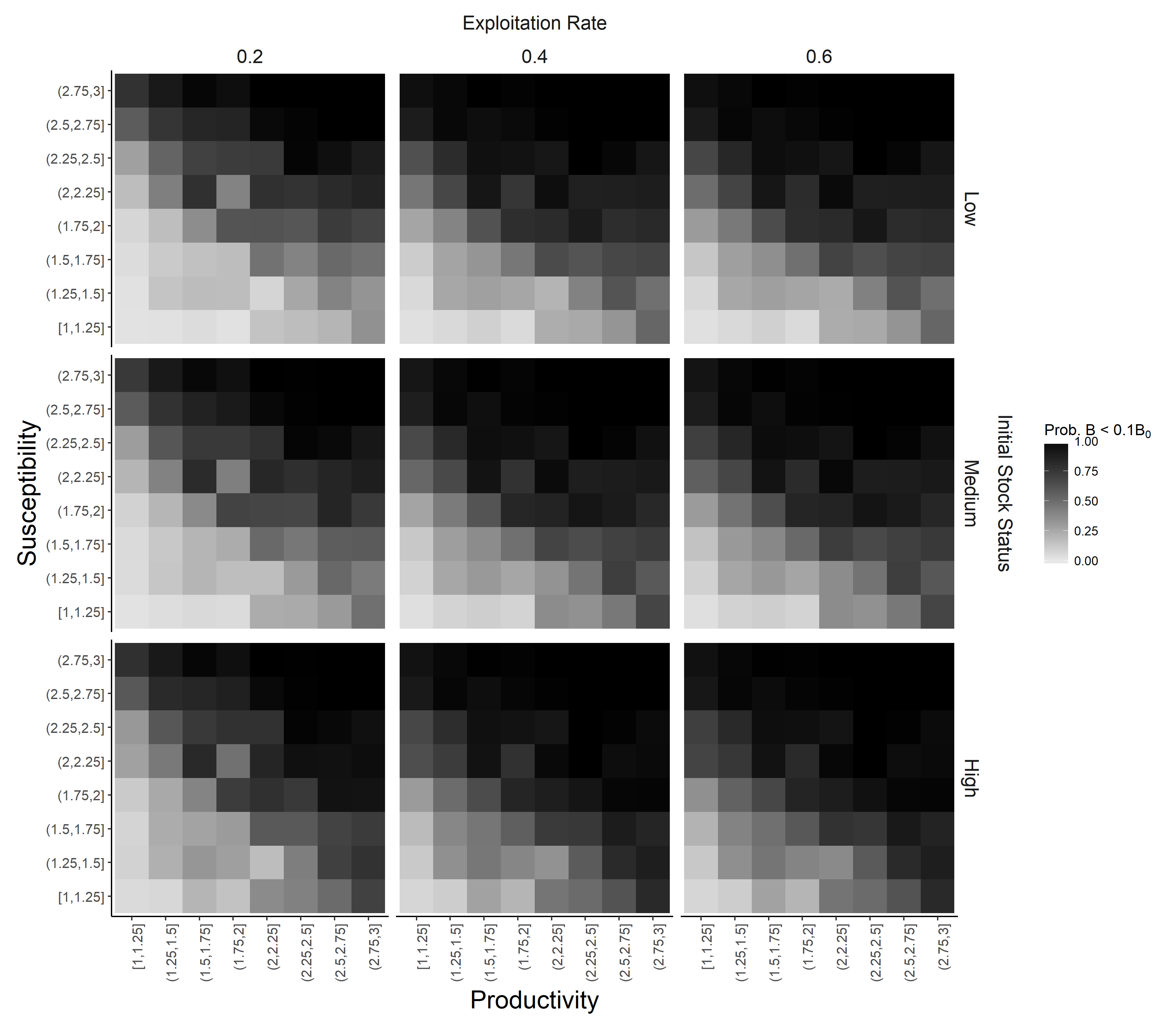

Supplement: S6 Fig — Risk in each plot has been standardized to a minimum and maximum value of 0 and 1. (PNG) [file pone.0198298.s006.png]

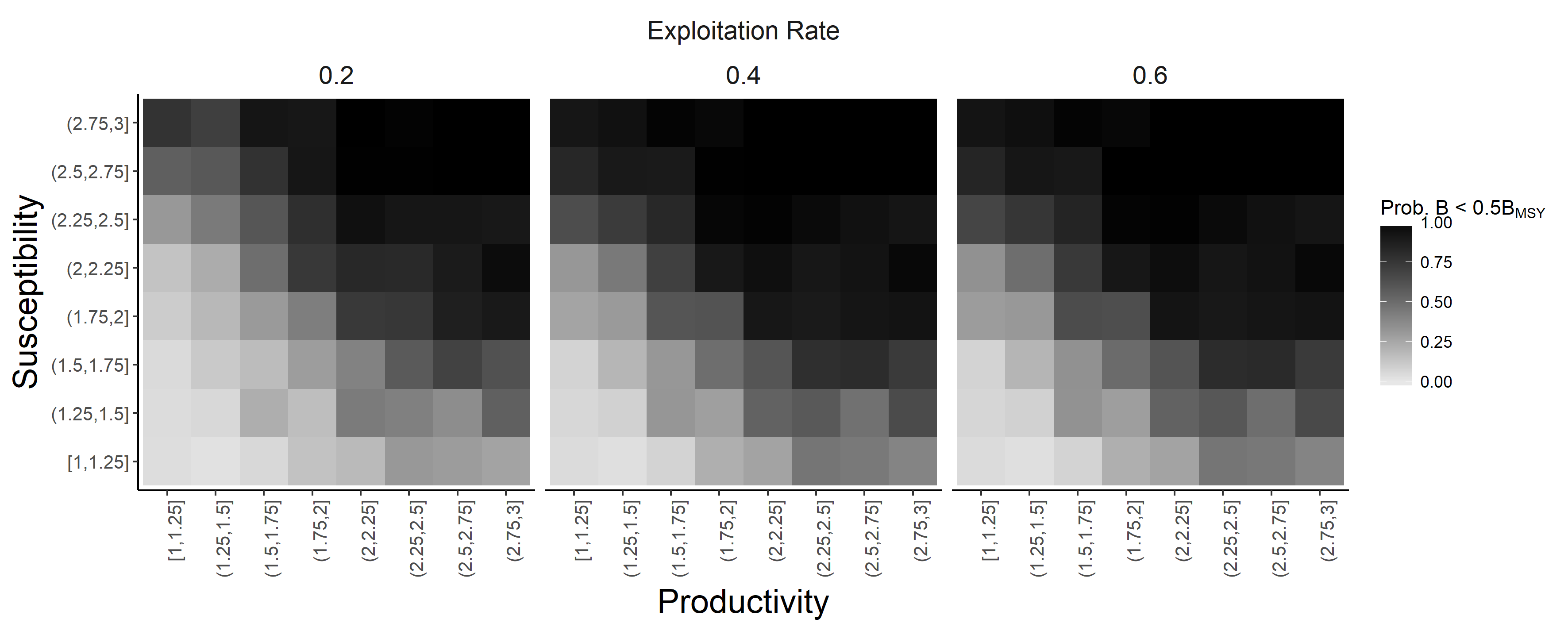

Supplement: S7 Fig — Risk in each plot has been standardized to a minimum and maximum value of 0 and 1. (PNG) [file pone.0198298.s007.png]

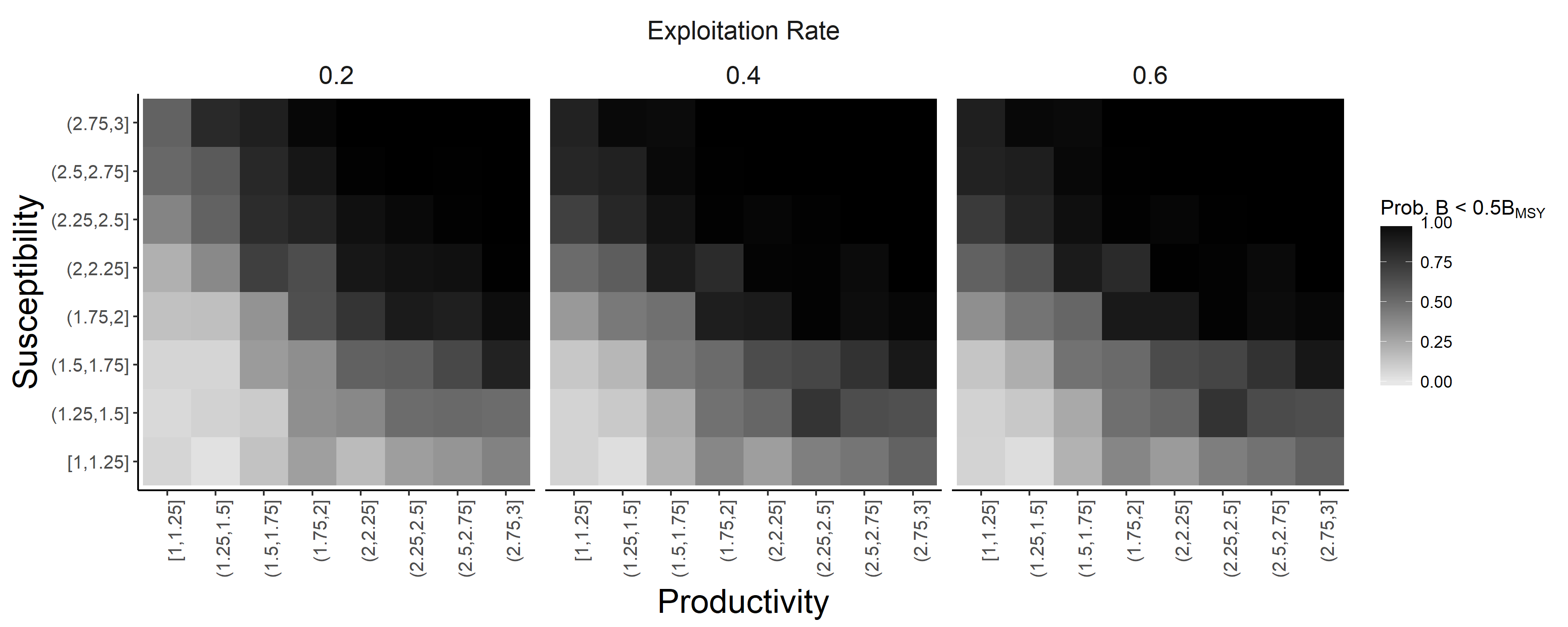

Supplement: S8 Fig — Risk in each plot has been standardized to a minimum and maximum value of 0 and 1. (PNG) [file pone.0198298.s008.png]

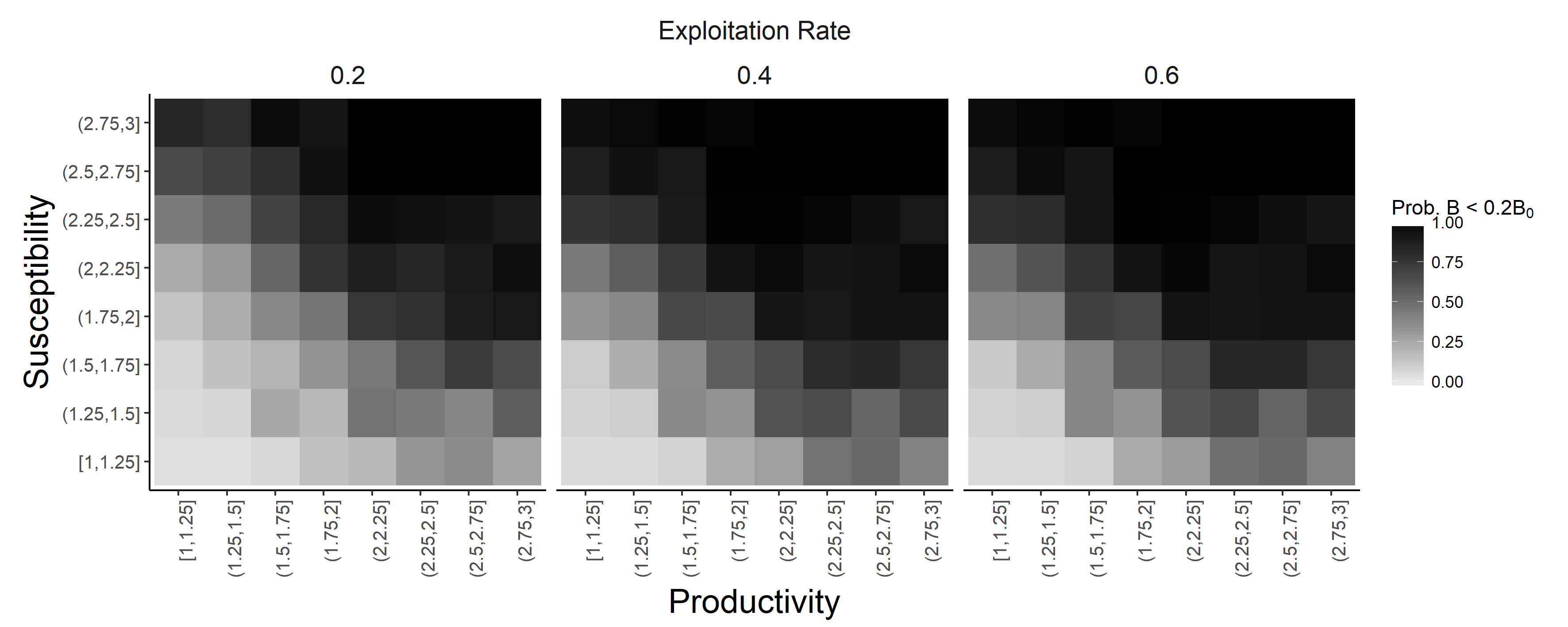

Supplement: S9 Fig — Risk in each plot has been standardized to a minimum and maximum value of 0 and 1. (PNG) [file pone.0198298.s009.png]

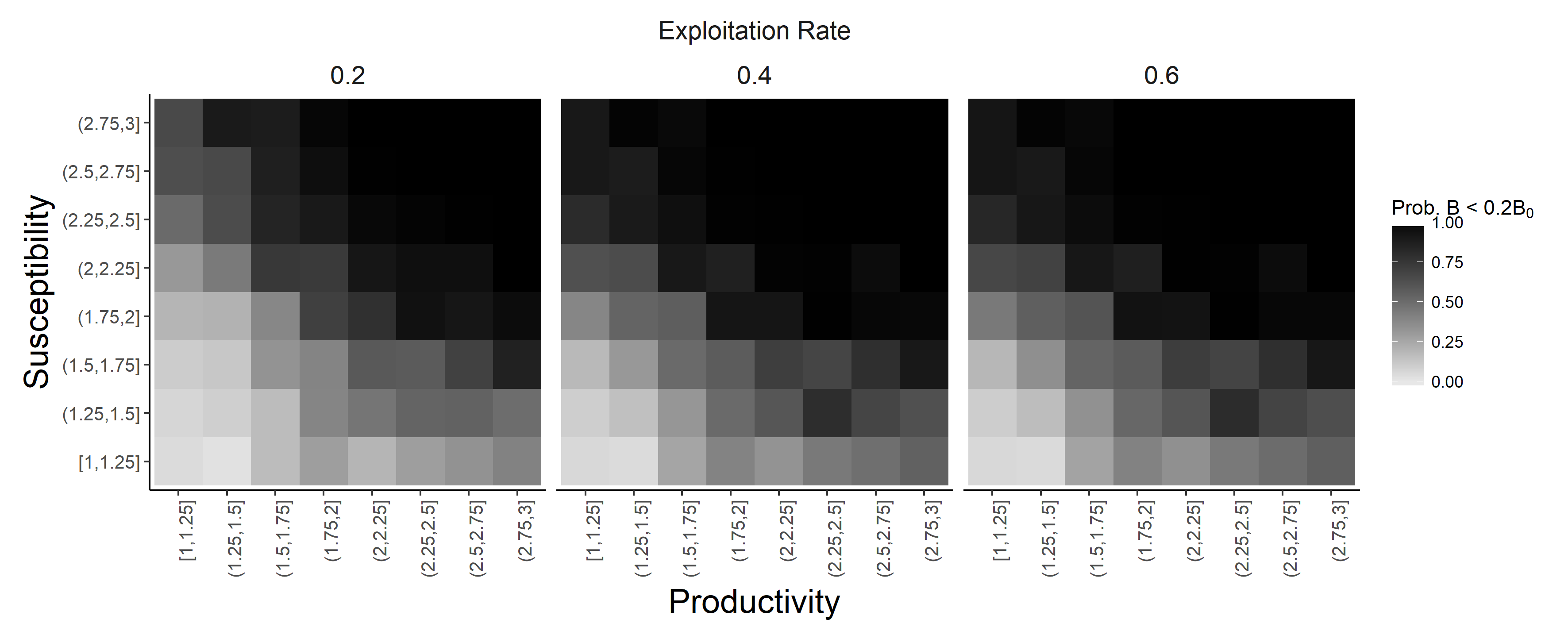

Supplement: S10 Fig — Risk in each plot has been standardized to a minimum and maximum value of 0 and 1. (PNG) [file pone.0198298.s010.png]

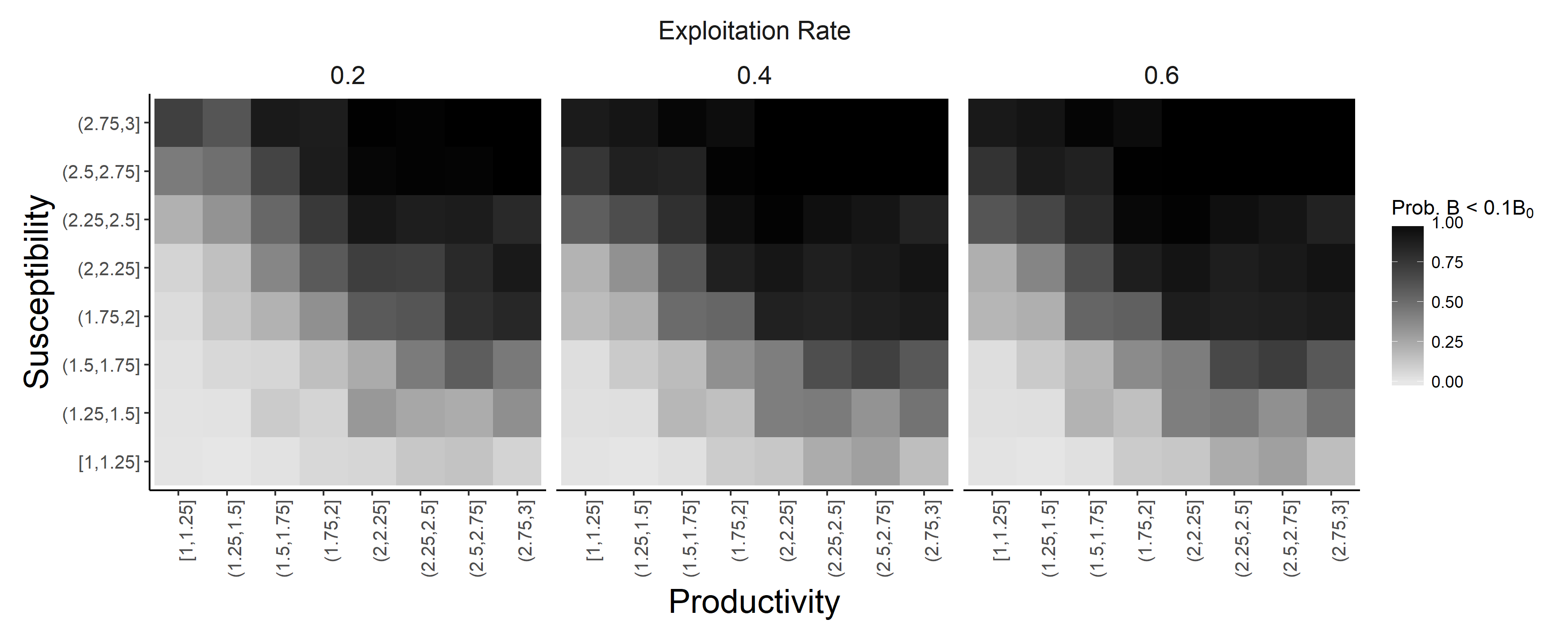

Supplement: S11 Fig — Risk in each plot has been standardized to a minimum and maximum value of 0 and 1. (PNG) [file pone.0198298.s011.png]

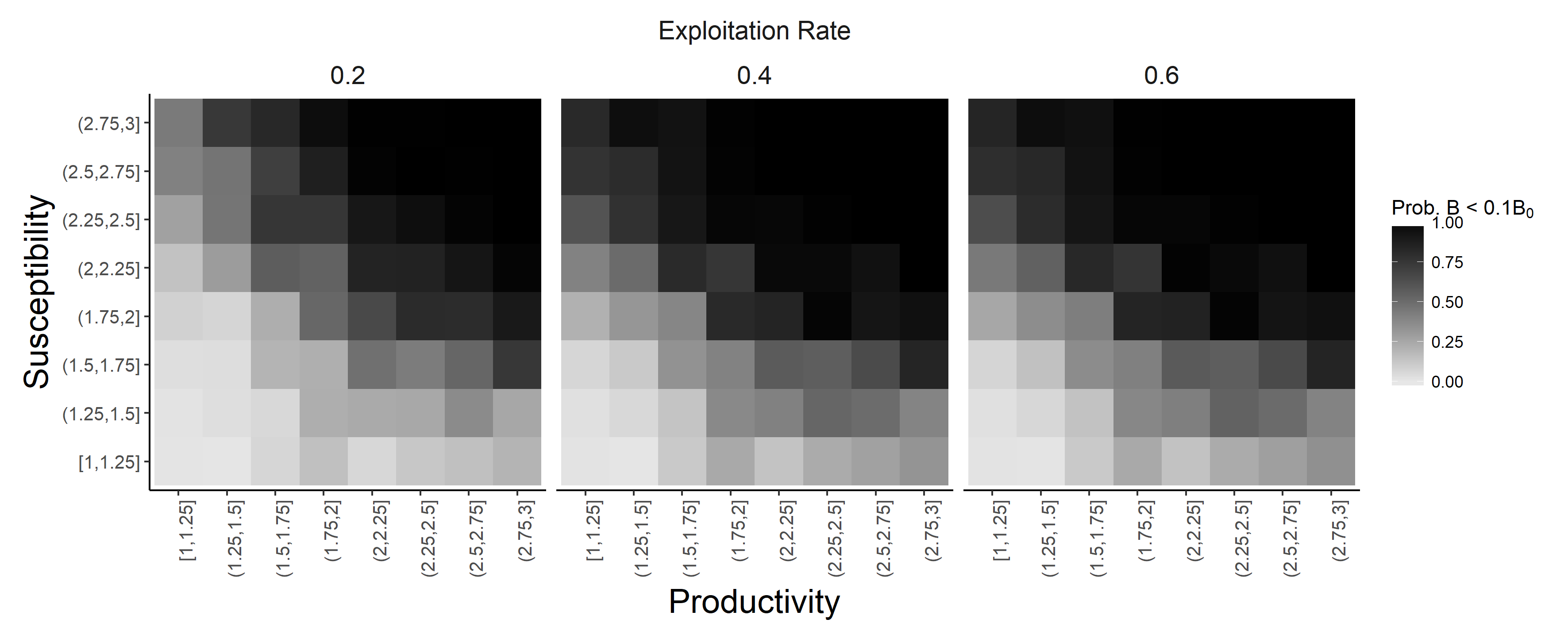

Supplement: S12 Fig — Risk in each plot has been standardized to a minimum and maximum value of 0 and 1. (PNG) [file pone.0198298.s012.png]

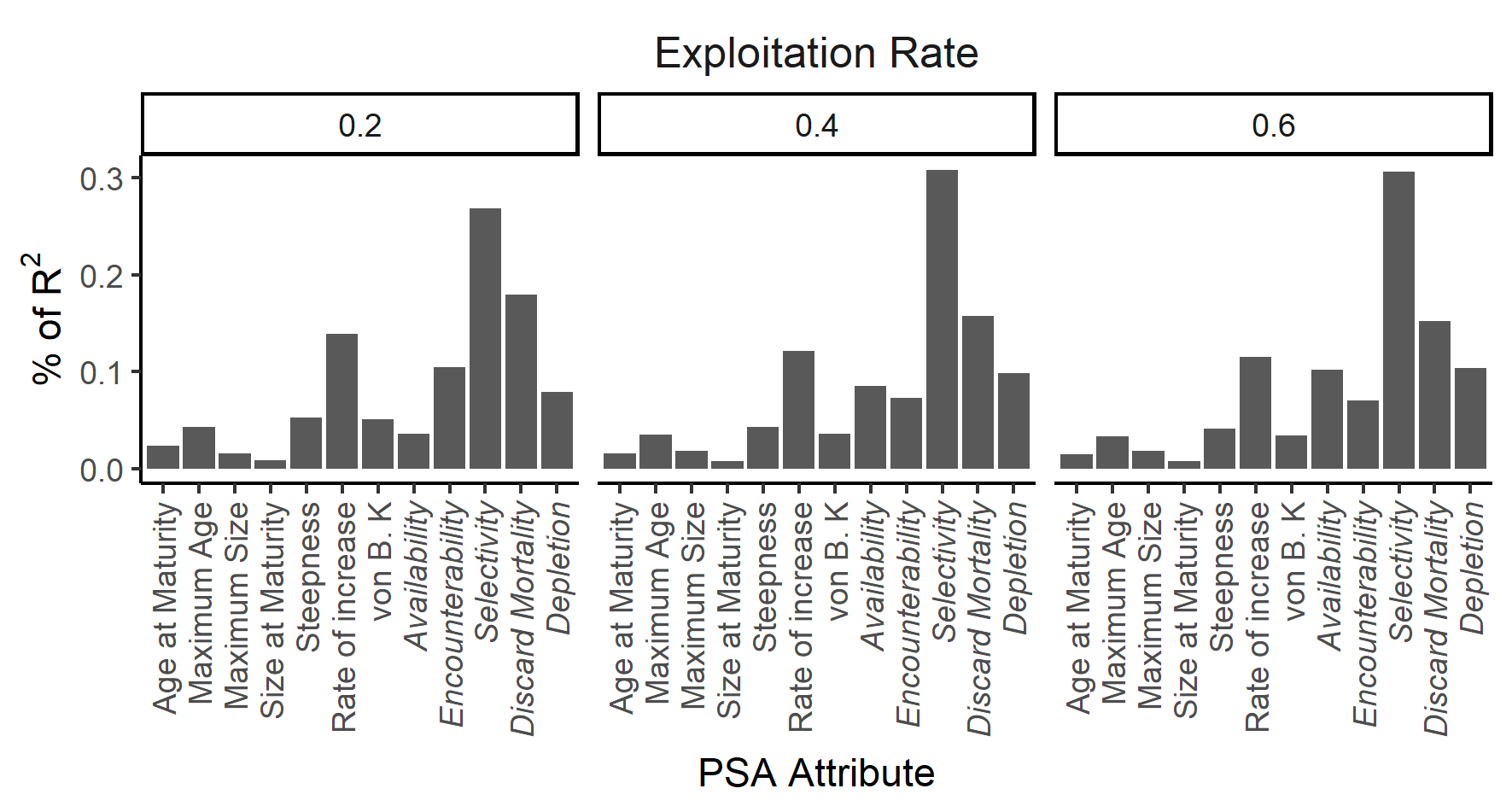

Supplement: S13 Fig — (PNG) [file pone.0198298.s013.png]

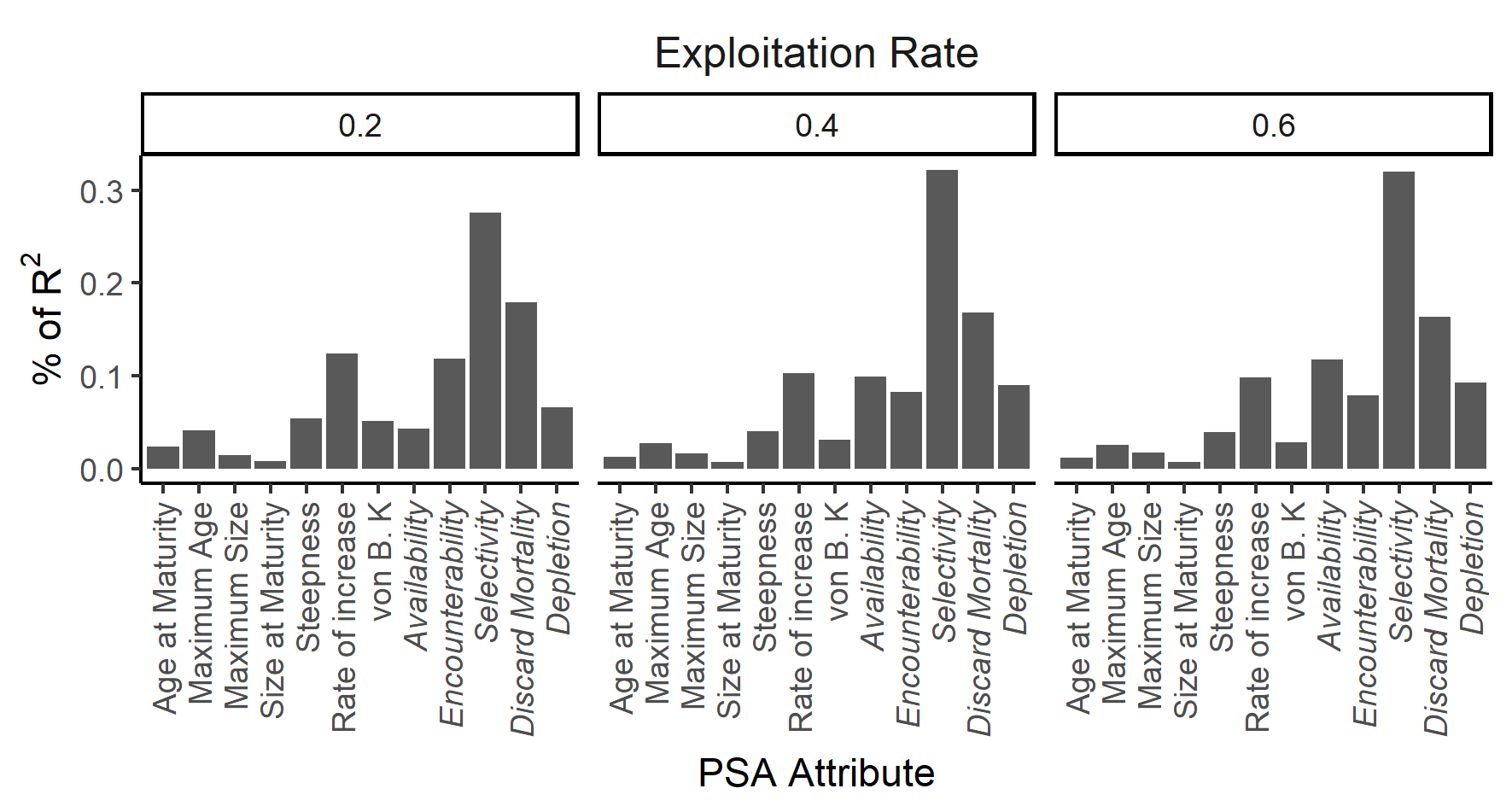

Supplement: S14 Fig — (PNG) [file pone.0198298.s014.png]

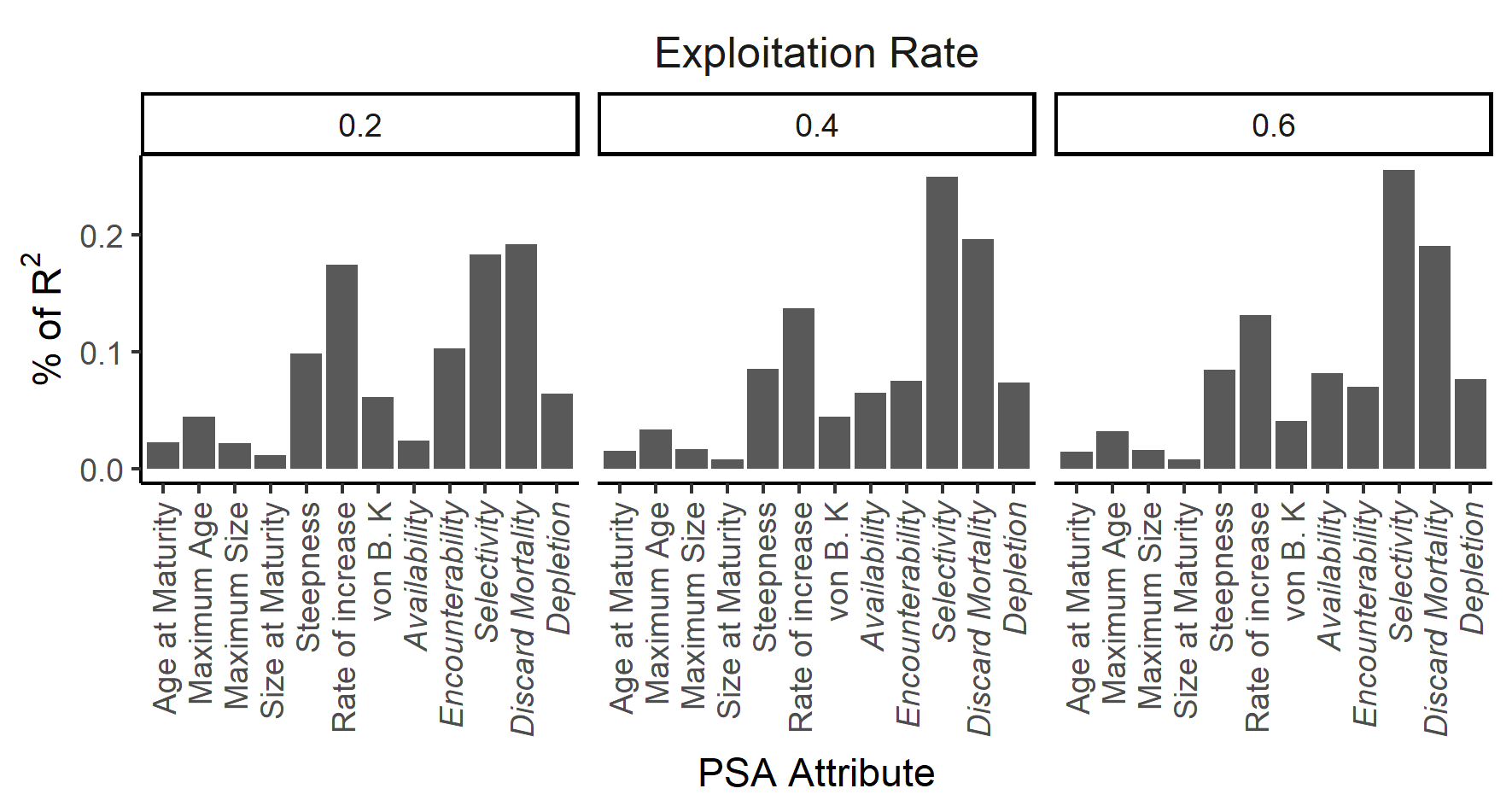

Supplement: S15 Fig — (PNG) [file pone.0198298.s015.png]

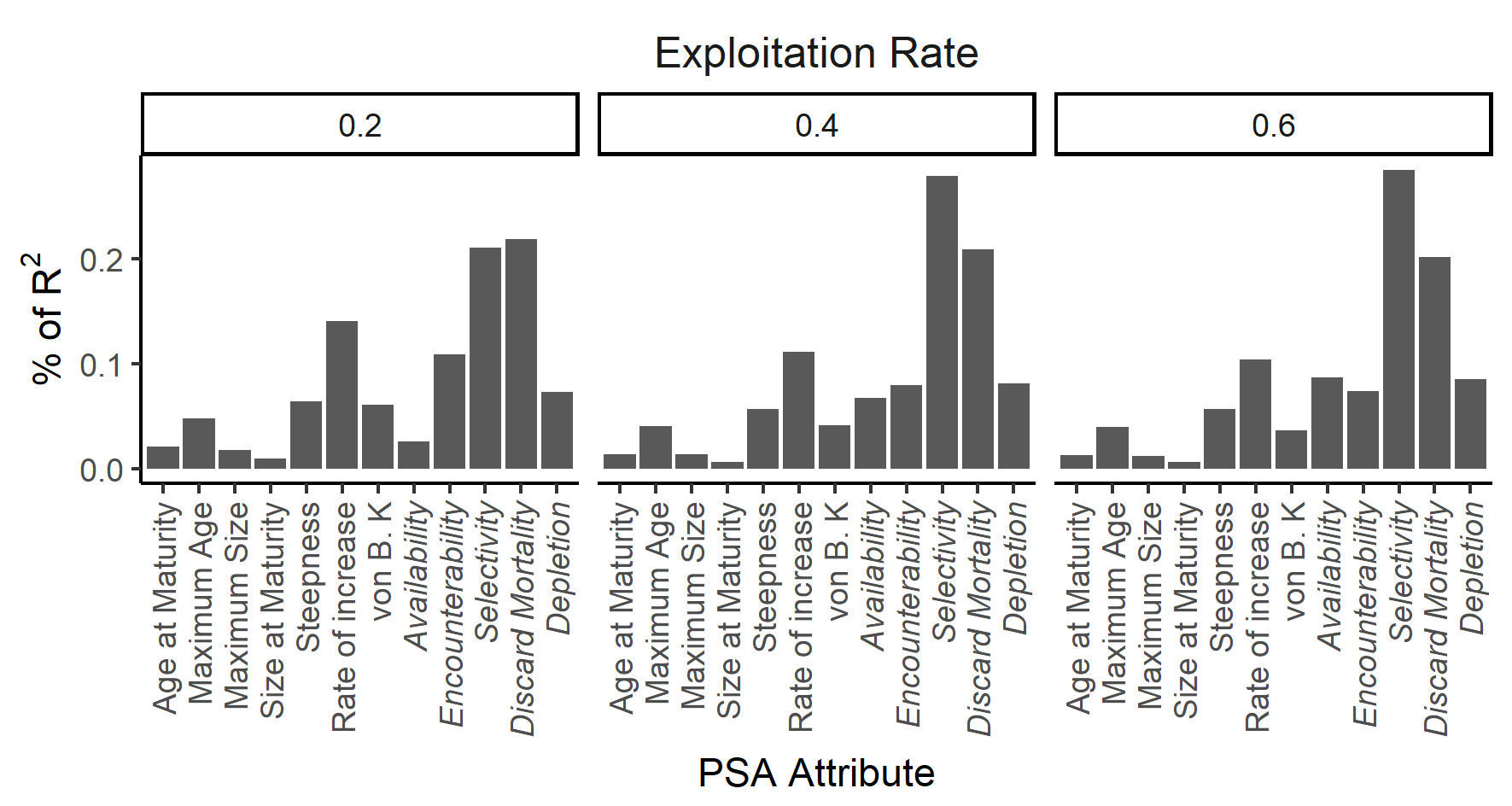

Supplement: S16 Fig — (PNG) [file pone.0198298.s016.png]

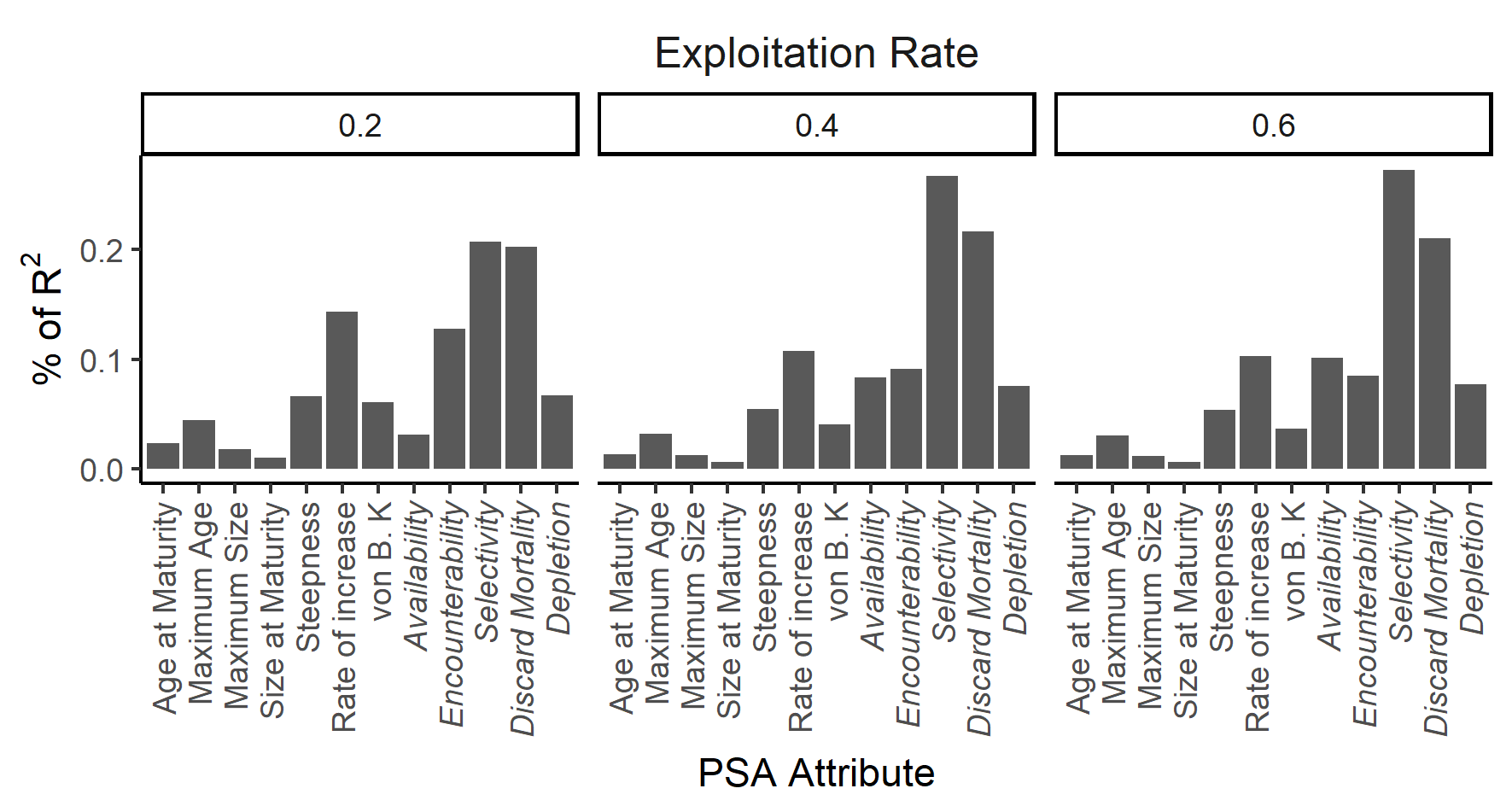

Supplement: S17 Fig — (PNG) [file pone.0198298.s017.png]

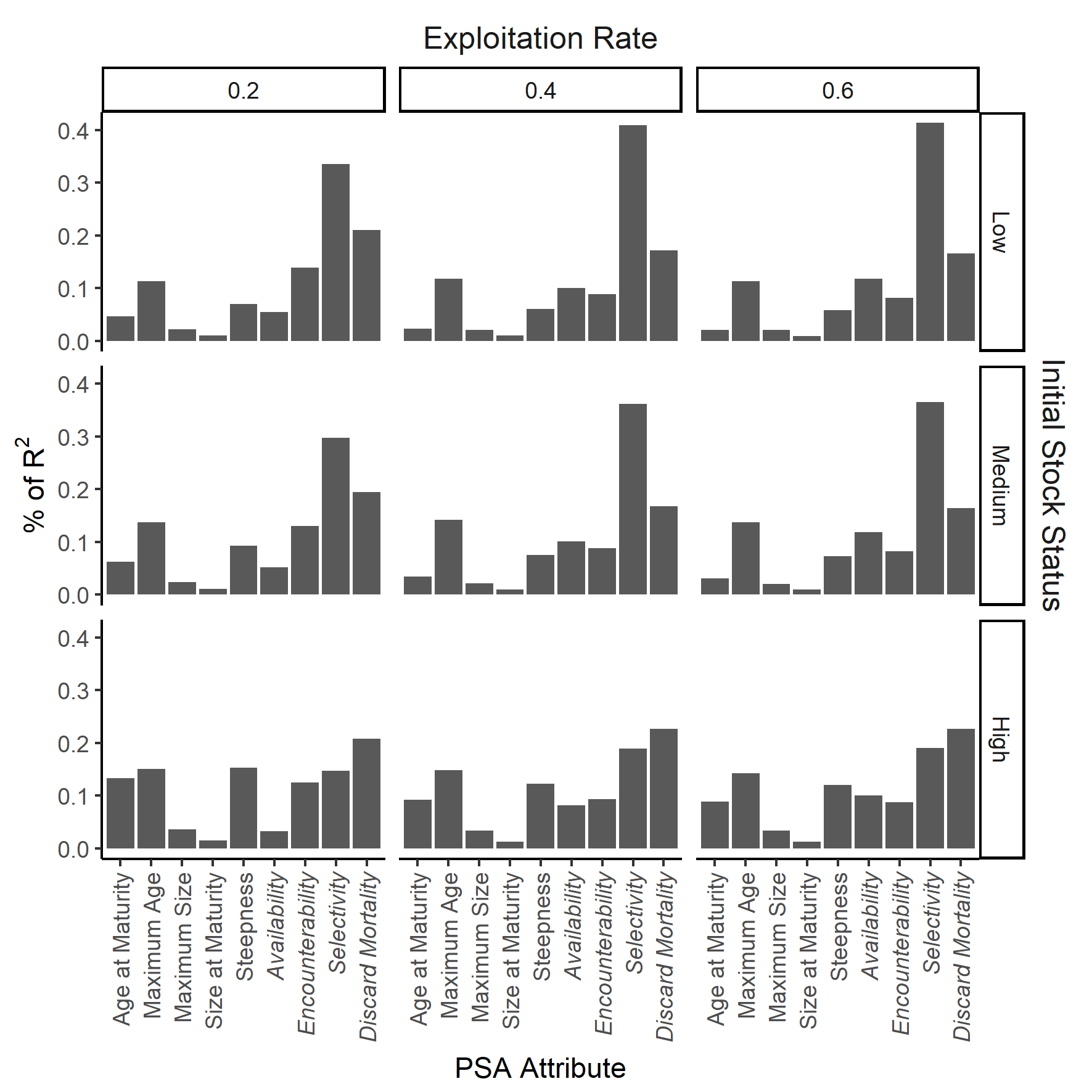

Supplement: S18 Fig — (PNG) [file pone.0198298.s018.png]

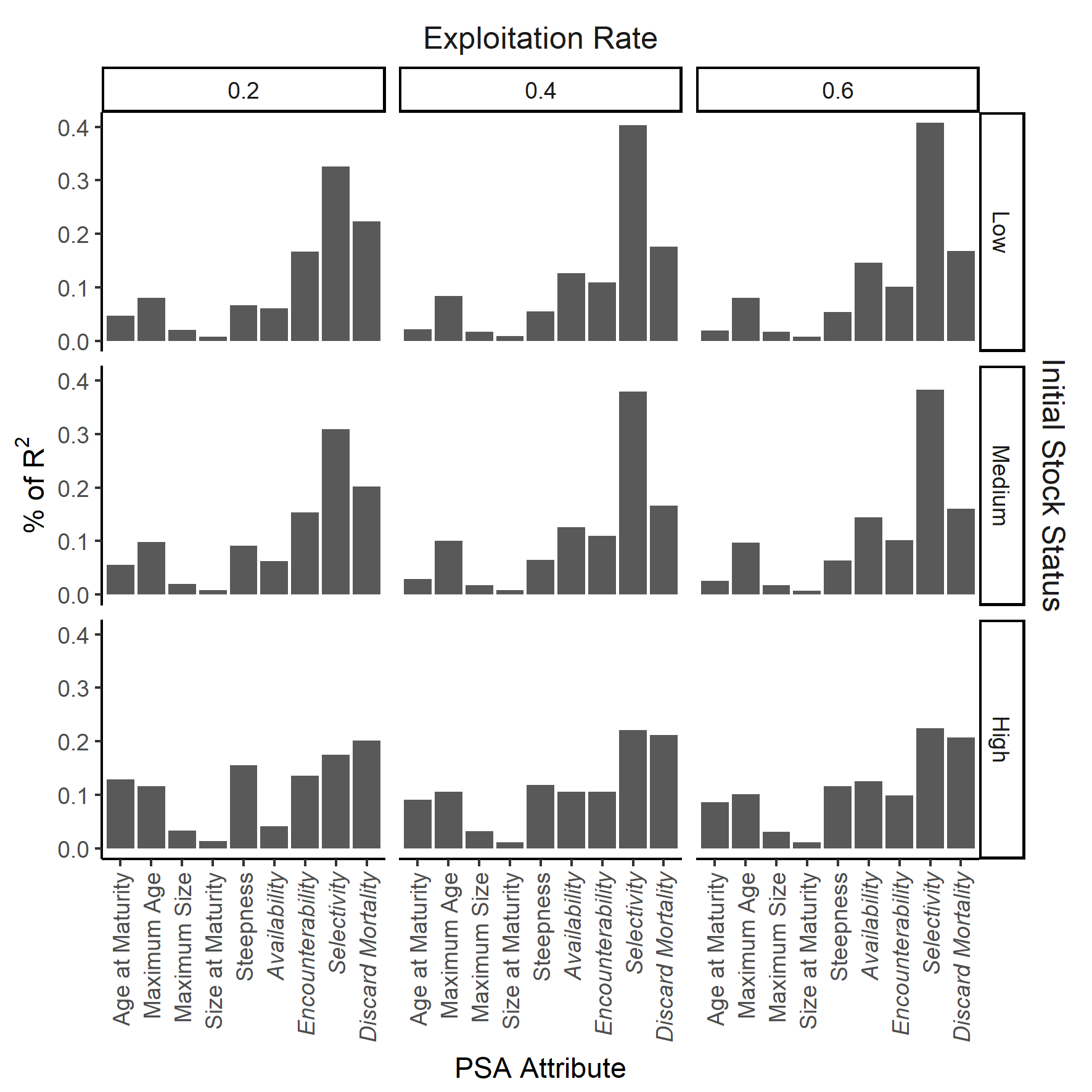

Supplement: S19 Fig — (PNG) [file pone.0198298.s019.png]

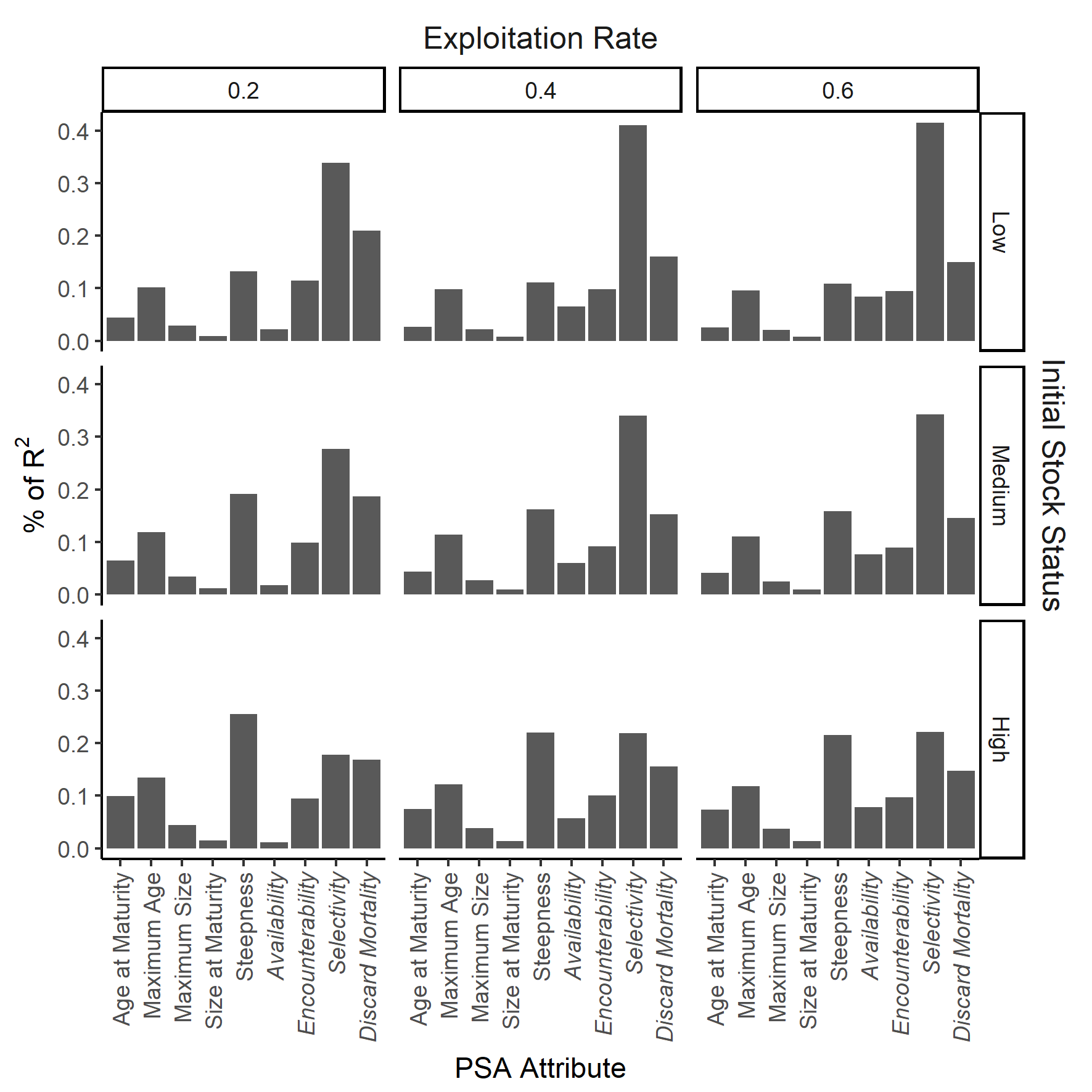

Supplement: S20 Fig — (PNG) [file pone.0198298.s020.png]

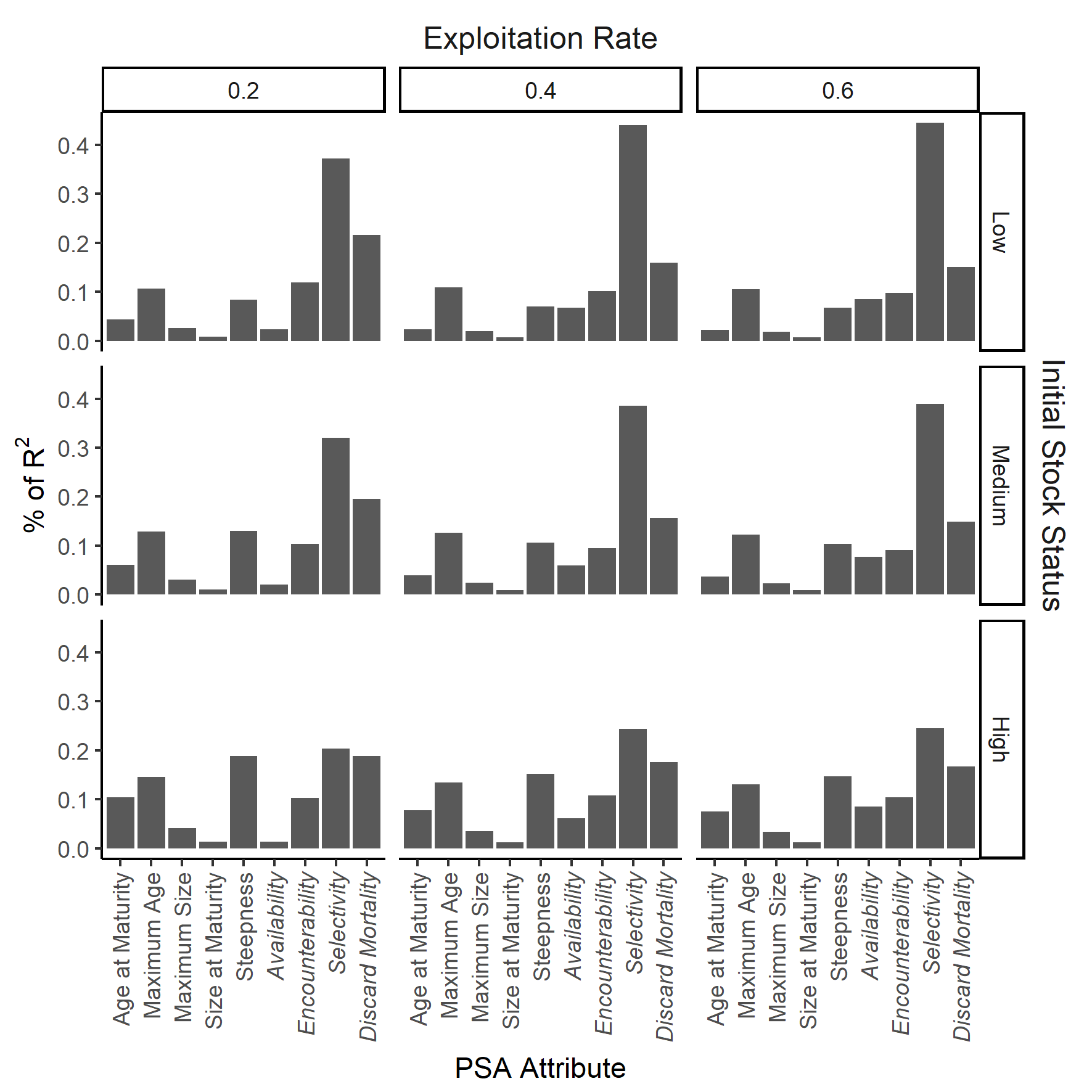

Supplement: S21 Fig — (PNG) [file pone.0198298.s021.png]

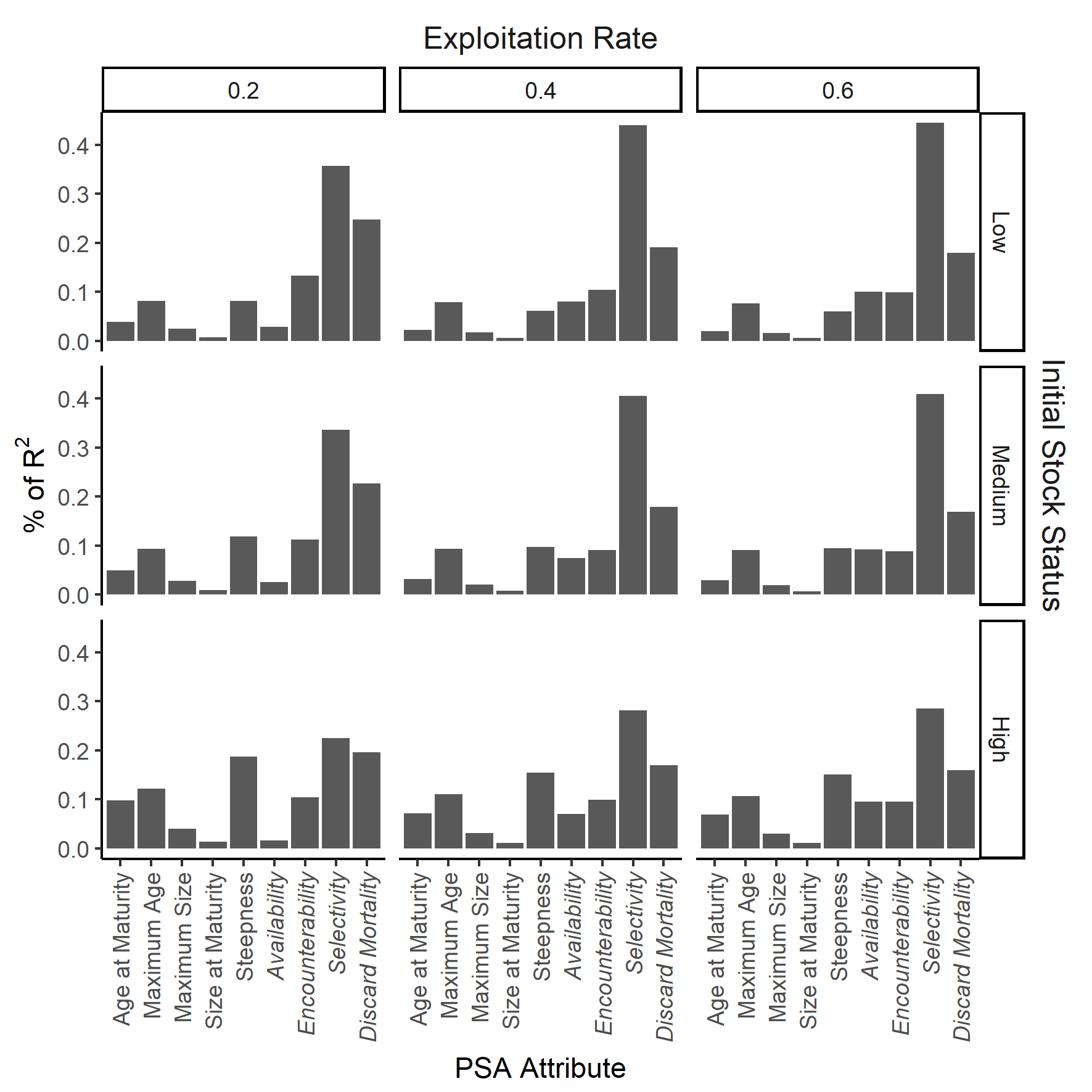

Supplement: S22 Fig — (PNG) [file pone.0198298.s022.png]

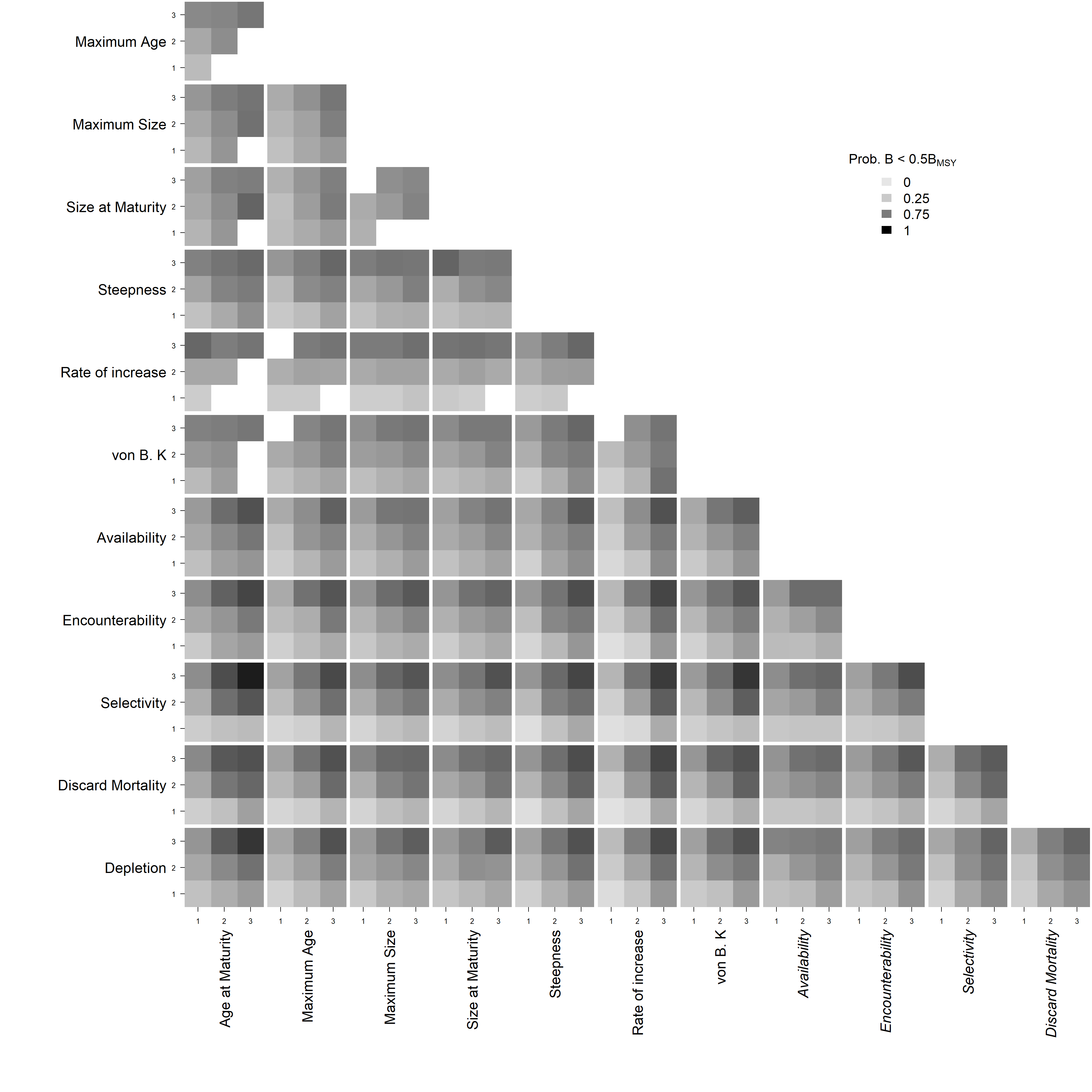

Supplement: S23 Fig — (PNG) [file pone.0198298.s023.png]

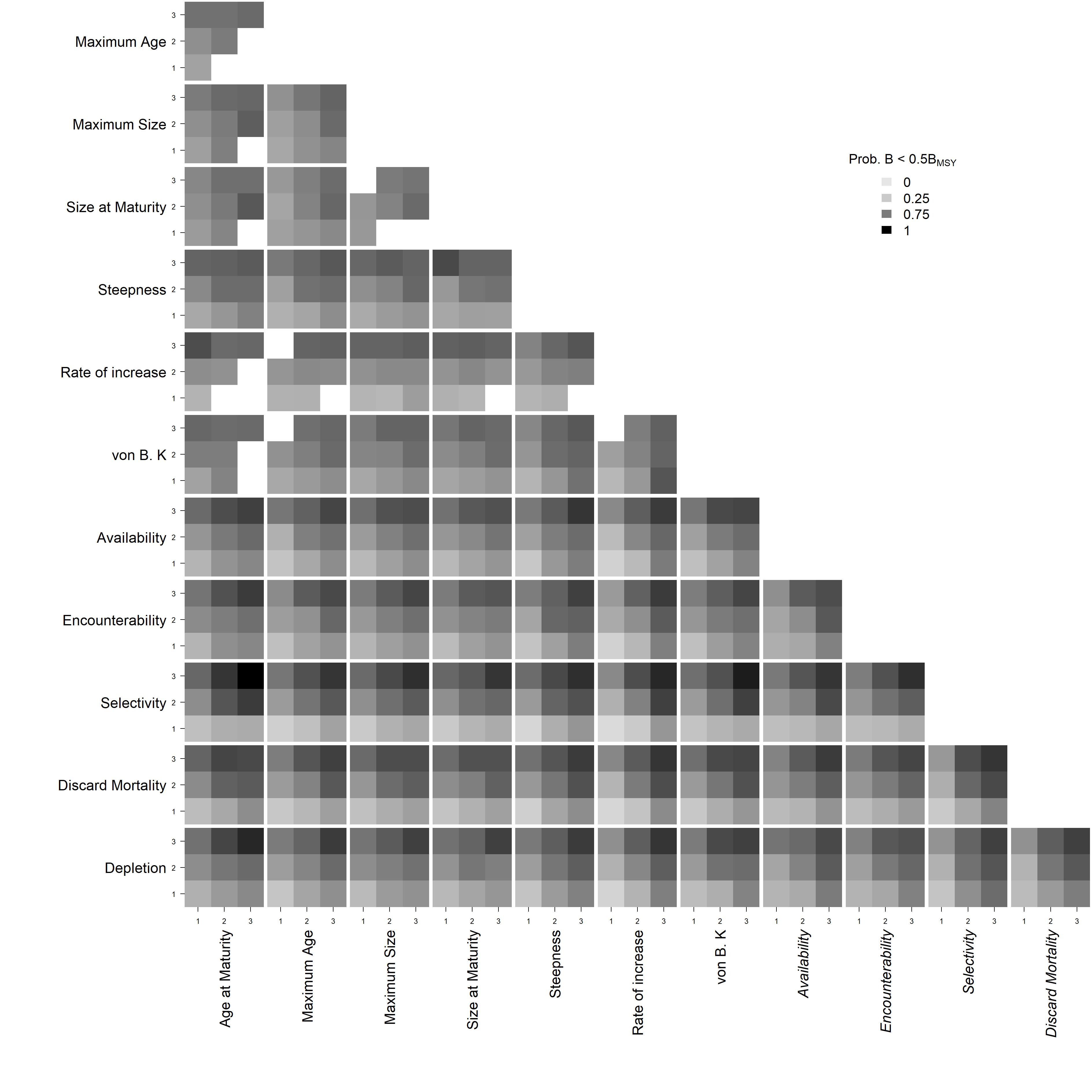

Supplement: S24 Fig — (PNG) [file pone.0198298.s024.png]

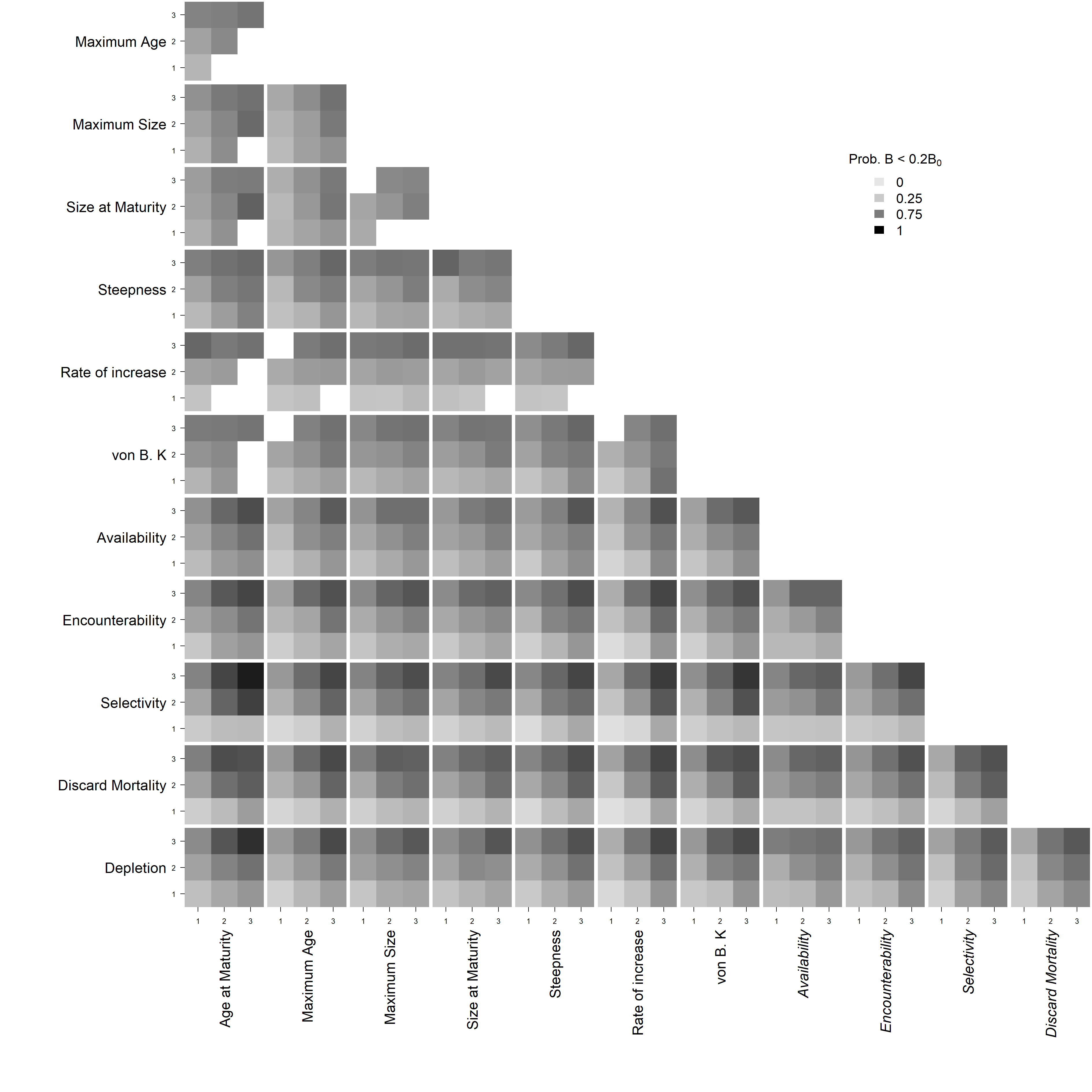

Supplement: S25 Fig — (PNG) [file pone.0198298.s025.png]

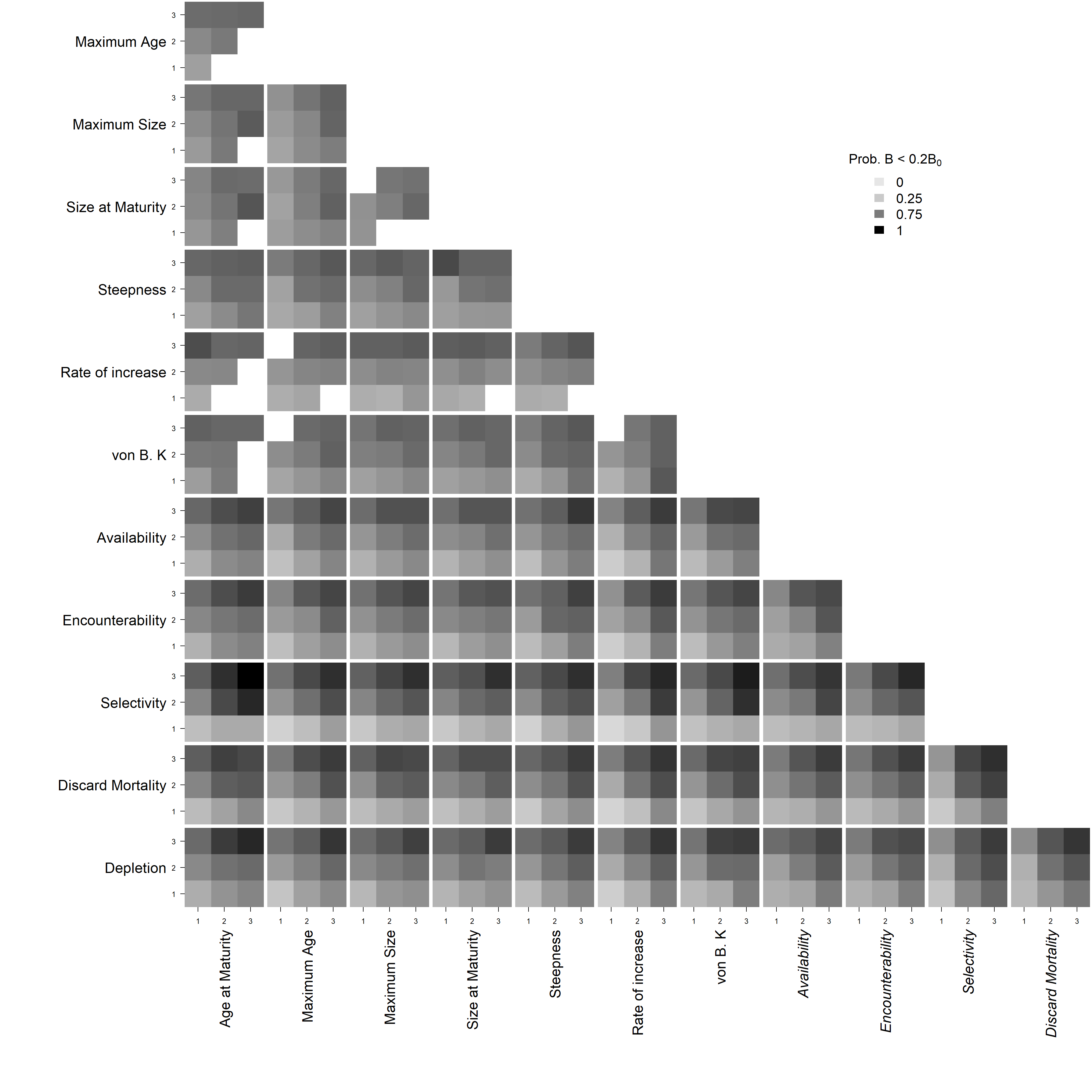

Supplement: S26 Fig — (PNG) [file pone.0198298.s026.png]

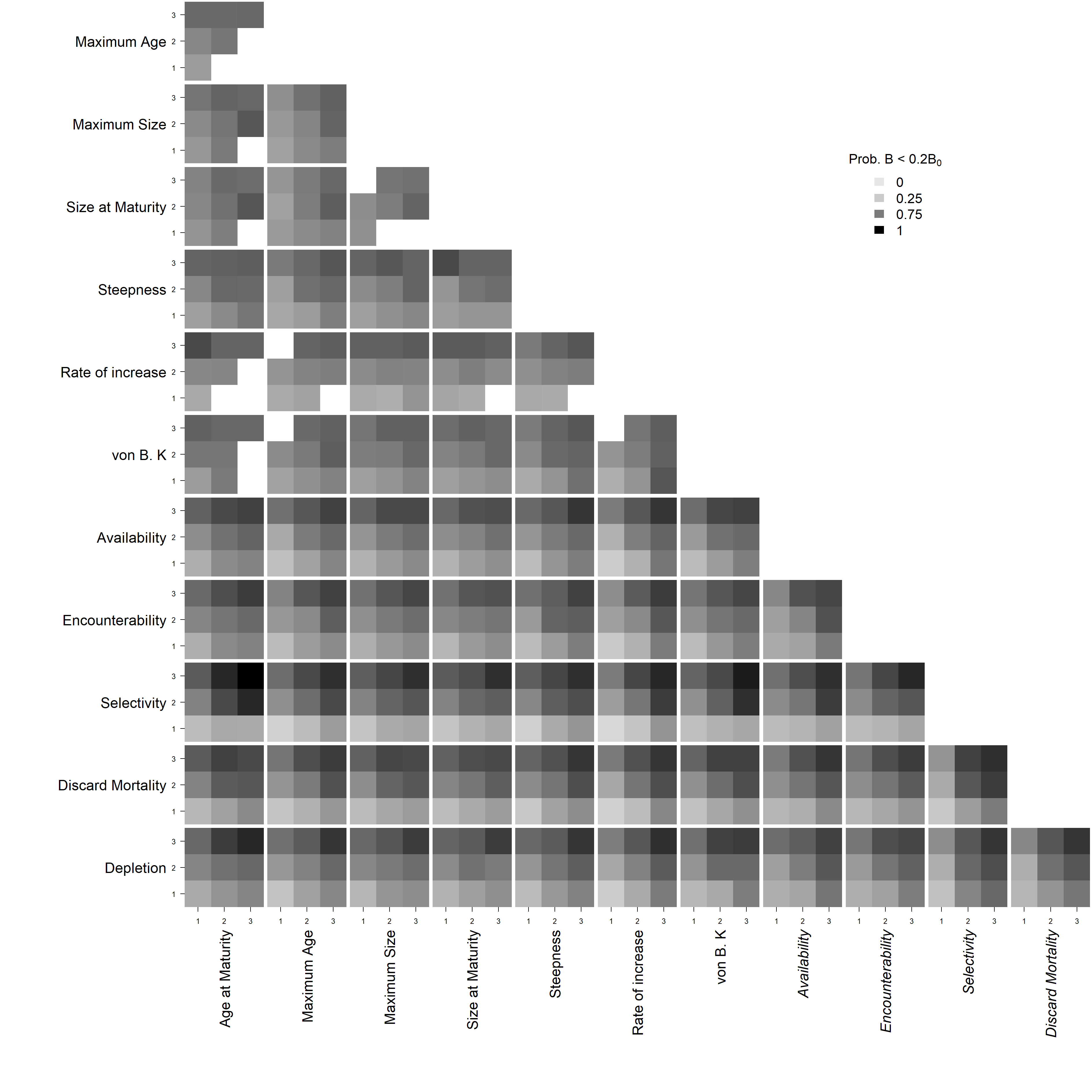

Supplement: S27 Fig — (PNG) [file pone.0198298.s027.png]

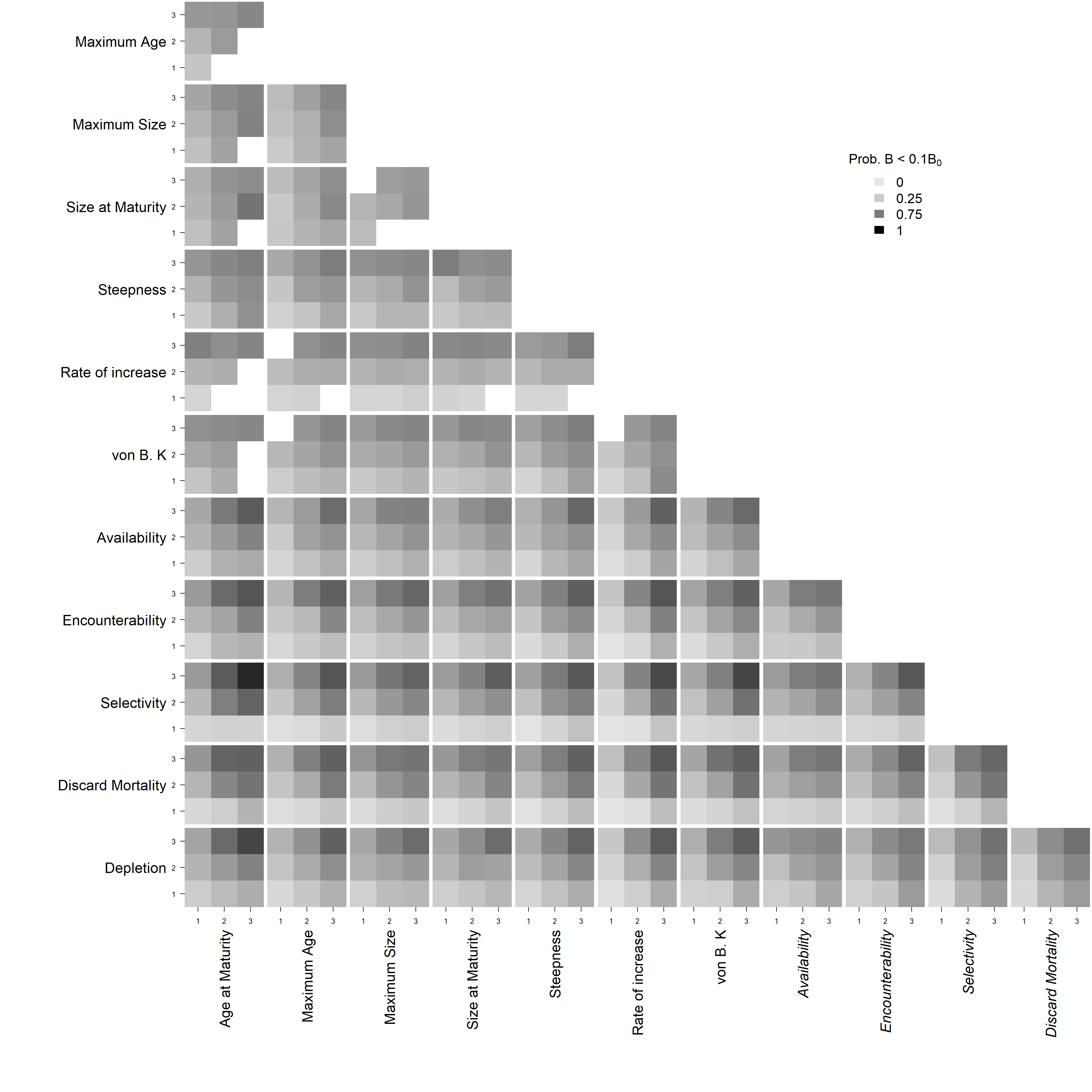

Supplement: S28 Fig — (PNG) [file pone.0198298.s028.png]

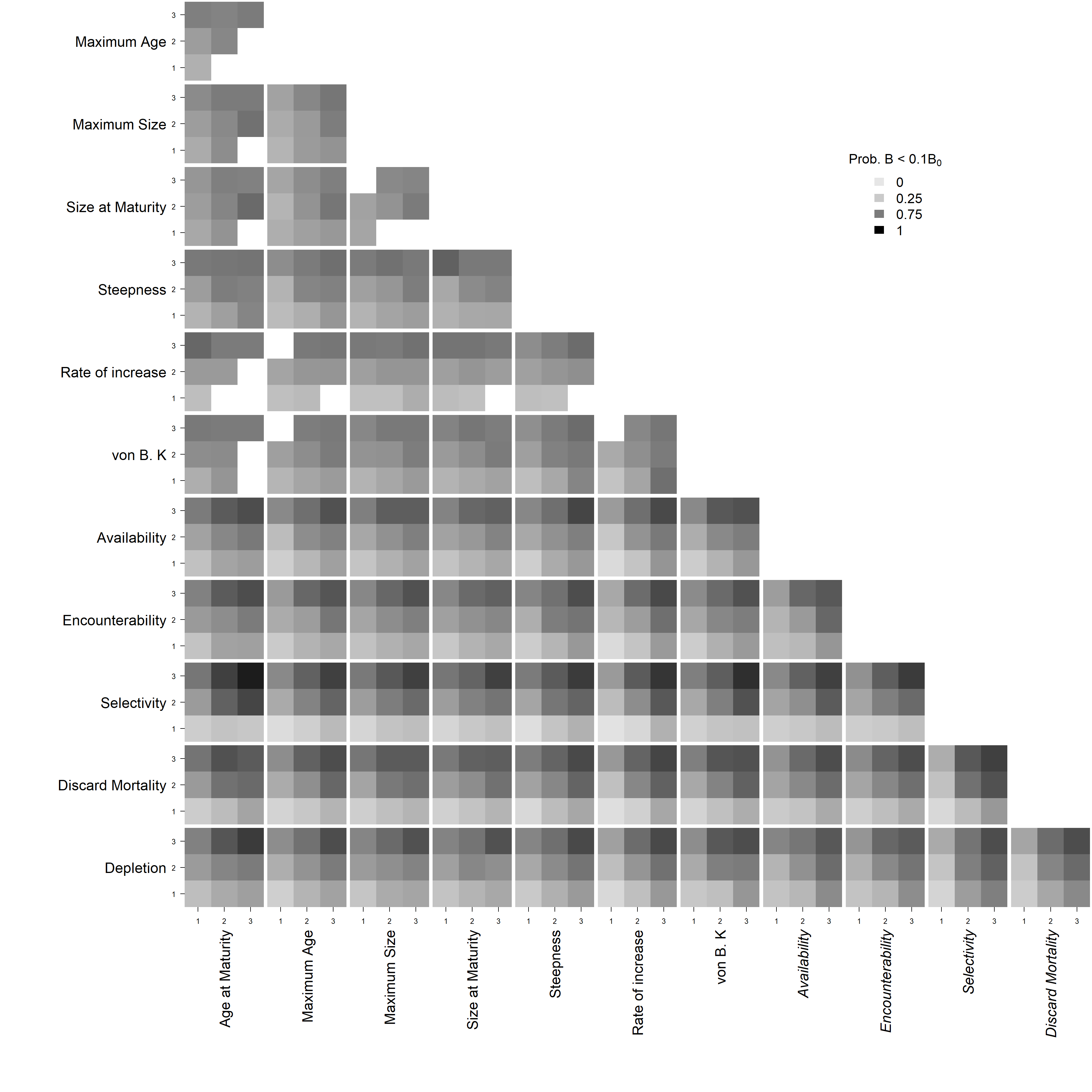

Supplement: S29 Fig — (PNG) [file pone.0198298.s029.png]

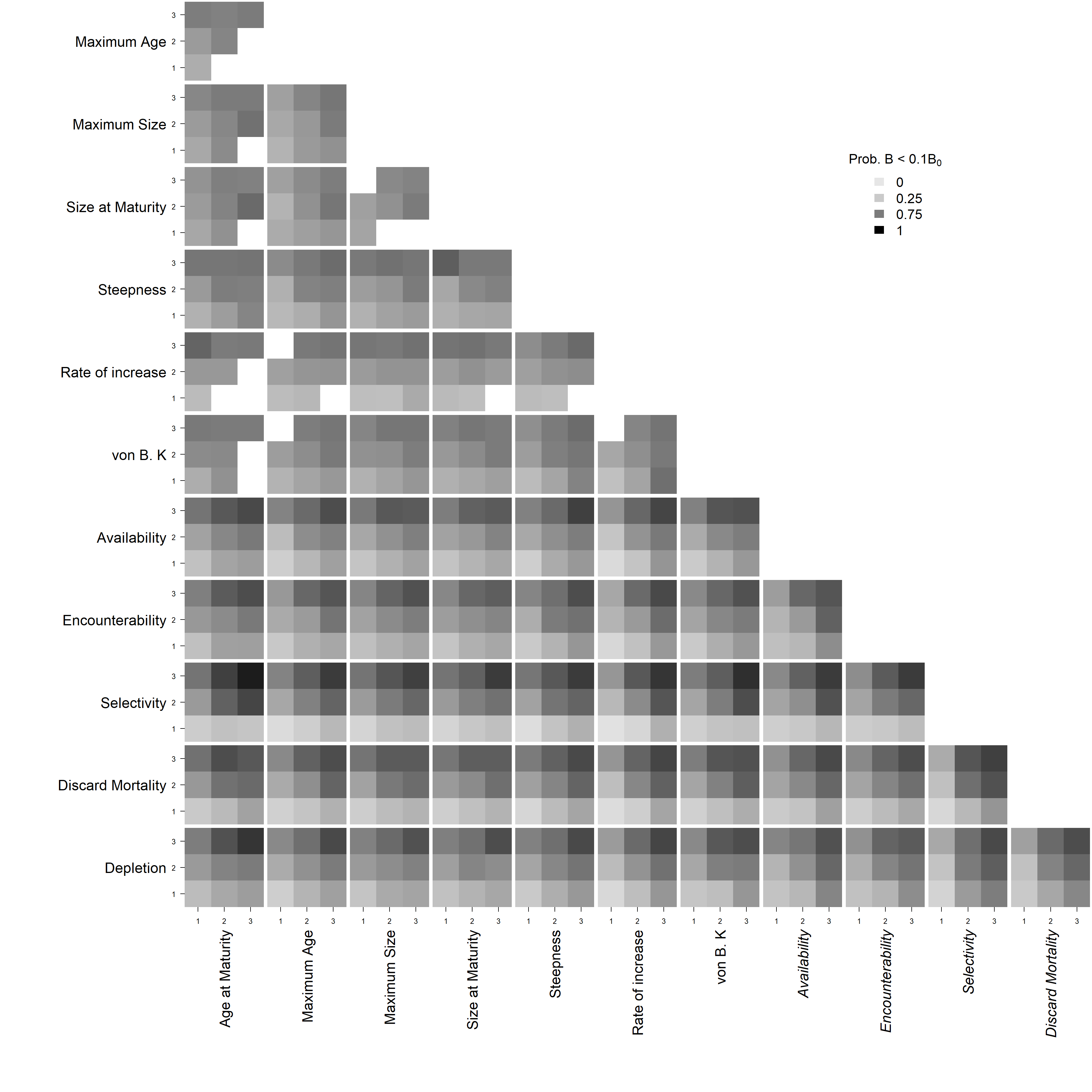

Supplement: S30 Fig — (PNG) [file pone.0198298.s030.png]

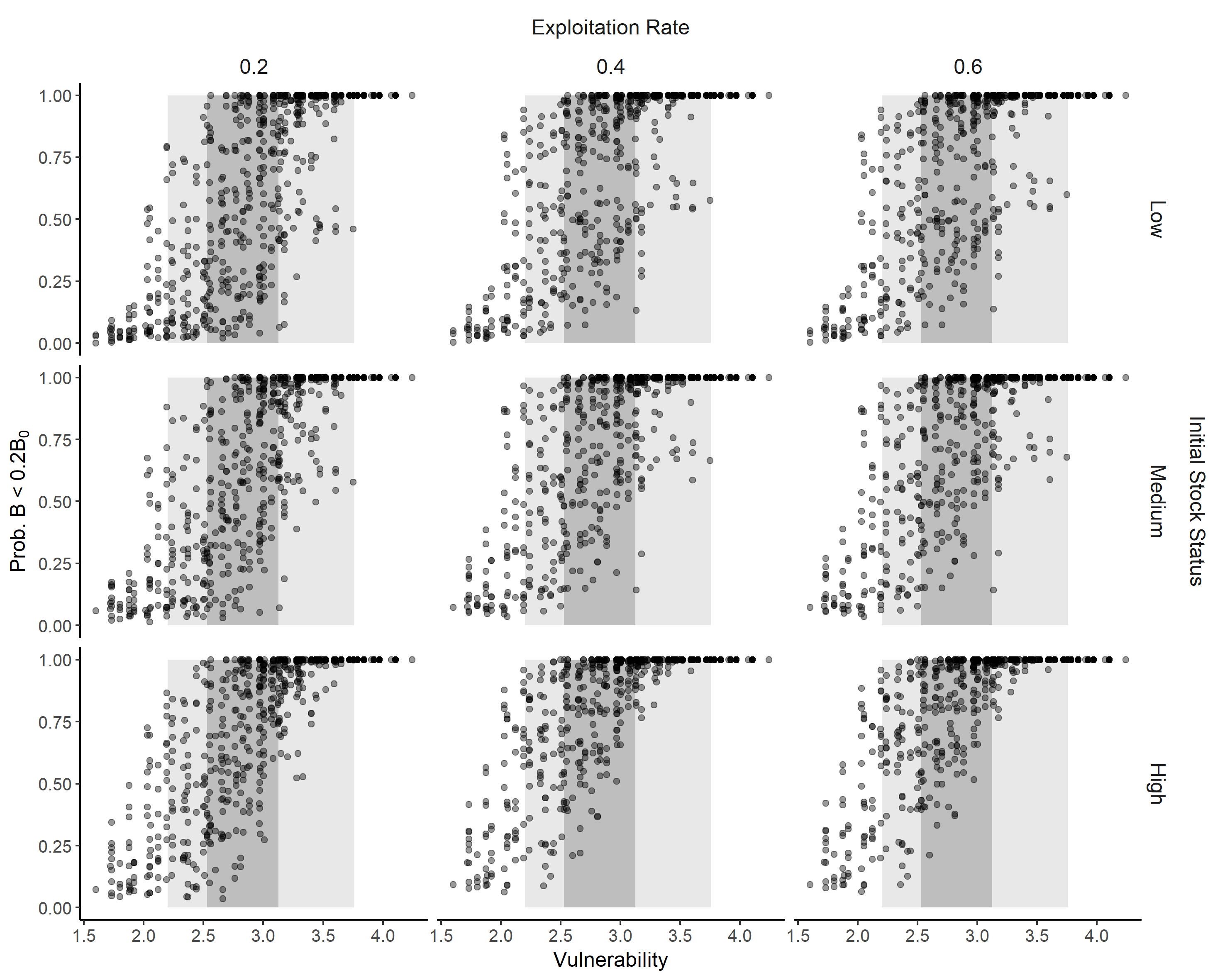

Supplement: S31 Fig — The gray shaded regions represent the 5th and 95th (light gray) and 25th and 75th (dark gray) percentiles of applications of the sPSA [17] and show that the scores for most applications fall within the mid-range values of the vulnerability score. (PNG) [file pone.0198298.s031.png]

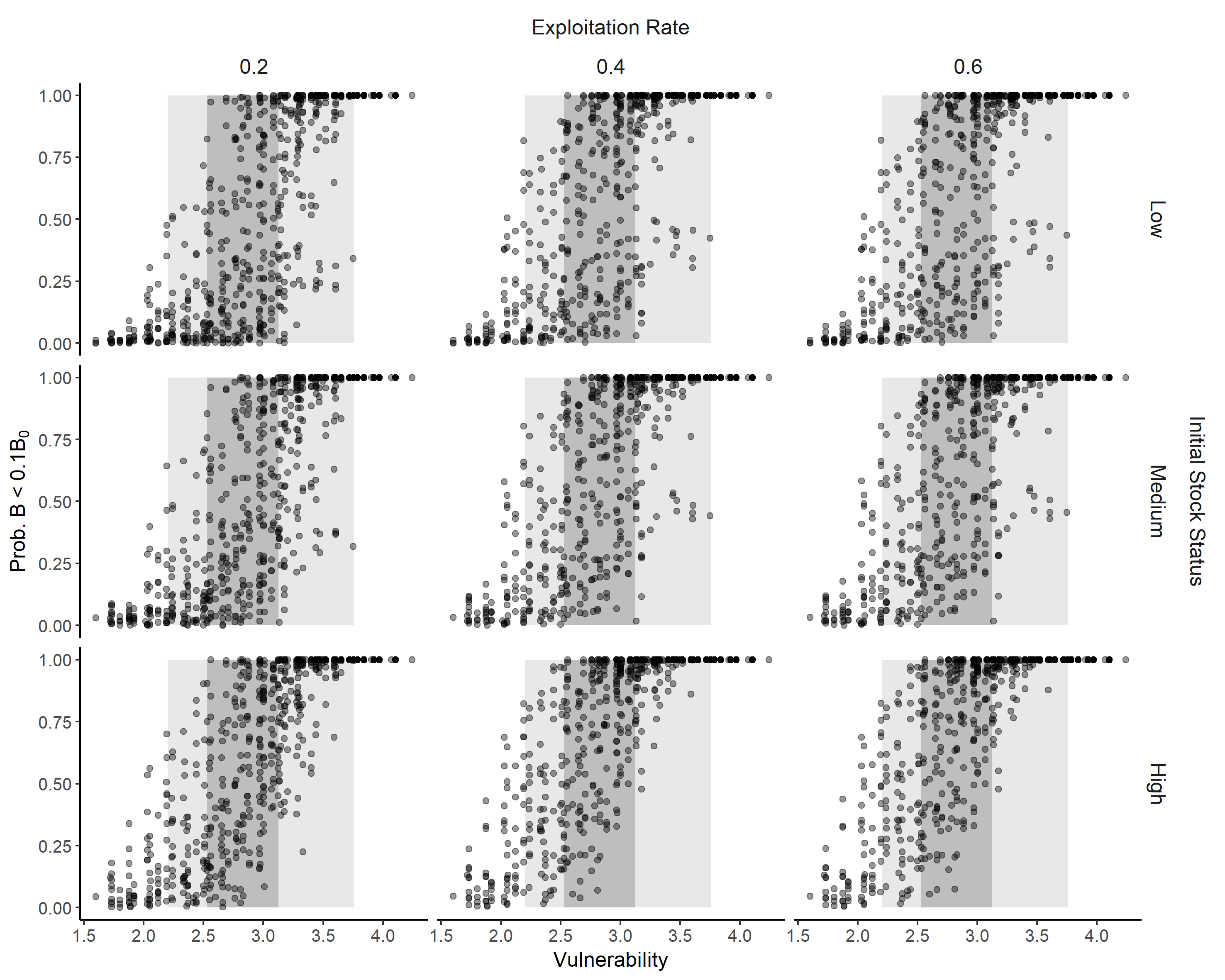

Supplement: S32 Fig — The gray shaded regions represent the 5th and 95th (light gray) and 25th and 75th (dark gray) percentiles of applications of the sPSA [17]and show that the scores for most applications fall within the mid-range values of the vulnerability score. (PNG) [file pone.0198298.s032.png]

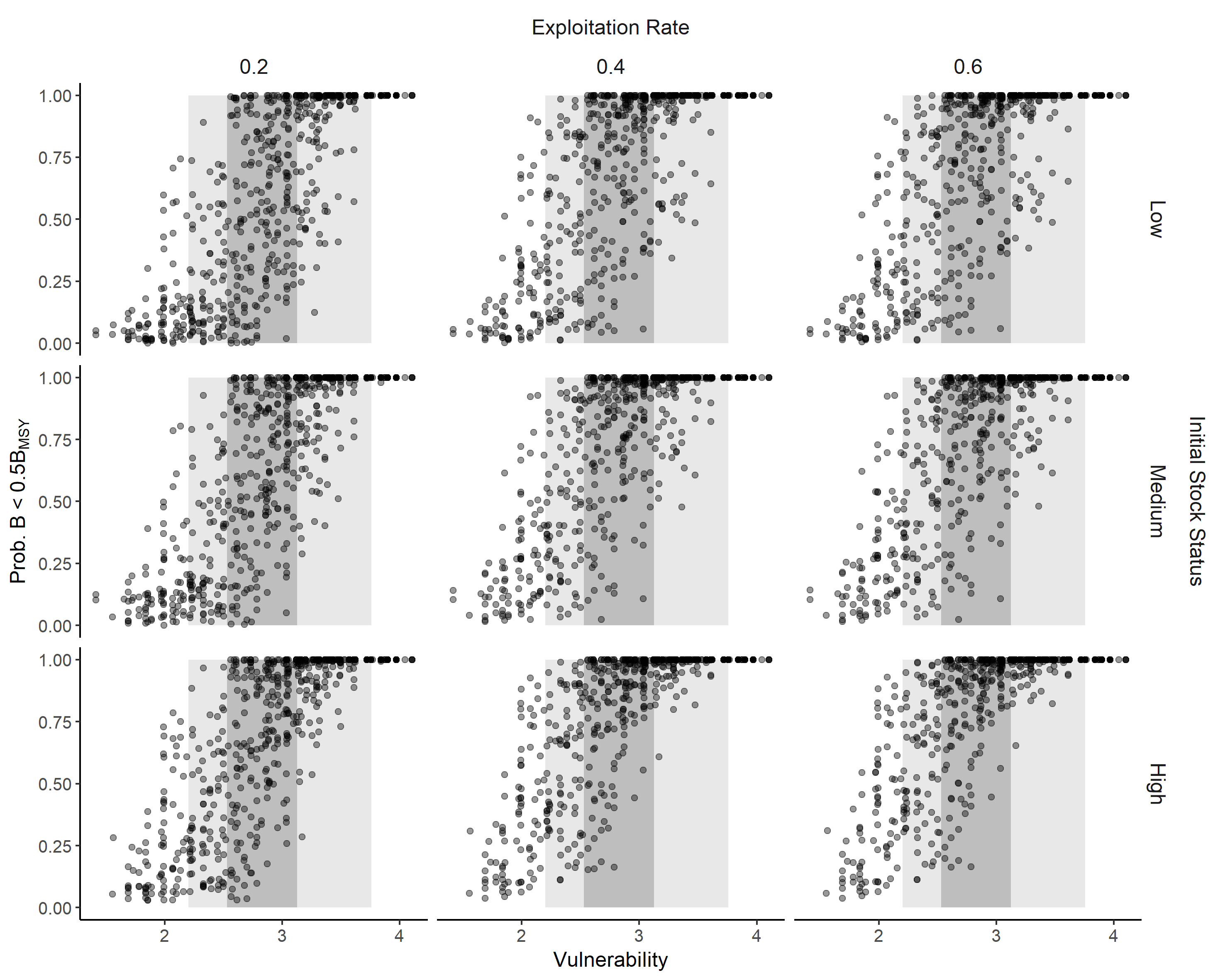

Supplement: S33 Fig — The gray shaded regions represent the 5th and 95th (light gray) and 25th and 75th (dark gray) percentiles of applications of the sPSA [17] and show that the scores for most applications fall within the mid-range values of the vulnerability score. (PNG) [file pone.0198298.s033.png]

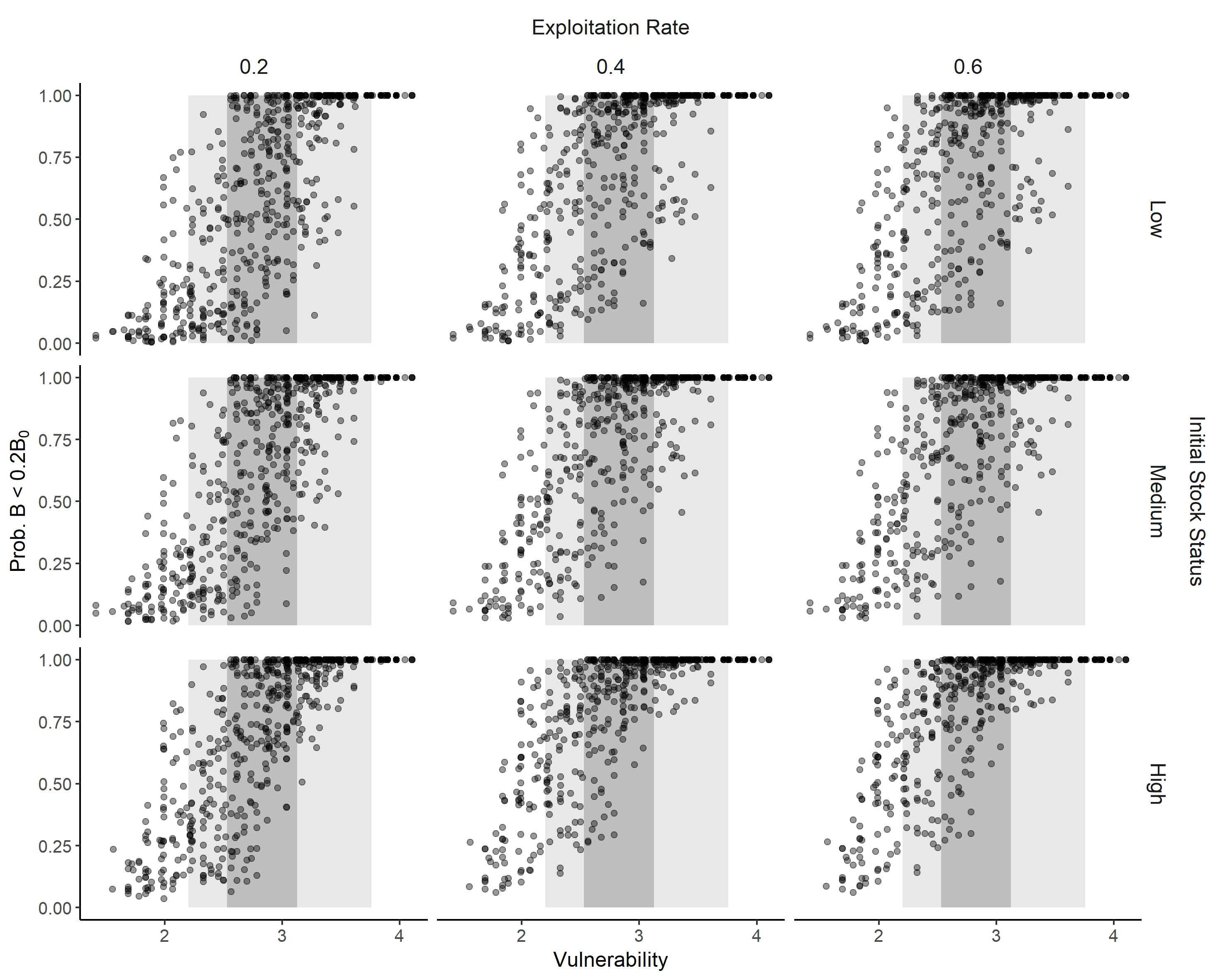

Supplement: S34 Fig — The gray shaded regions represent the 5th and 95th (light gray) and 25th and 75th (dark gray) percentiles of applications of the sPSA [17] and show that the scores for most applications fall within the mid-range values of the vulnerability score. (PNG) [file pone.0198298.s034.png]

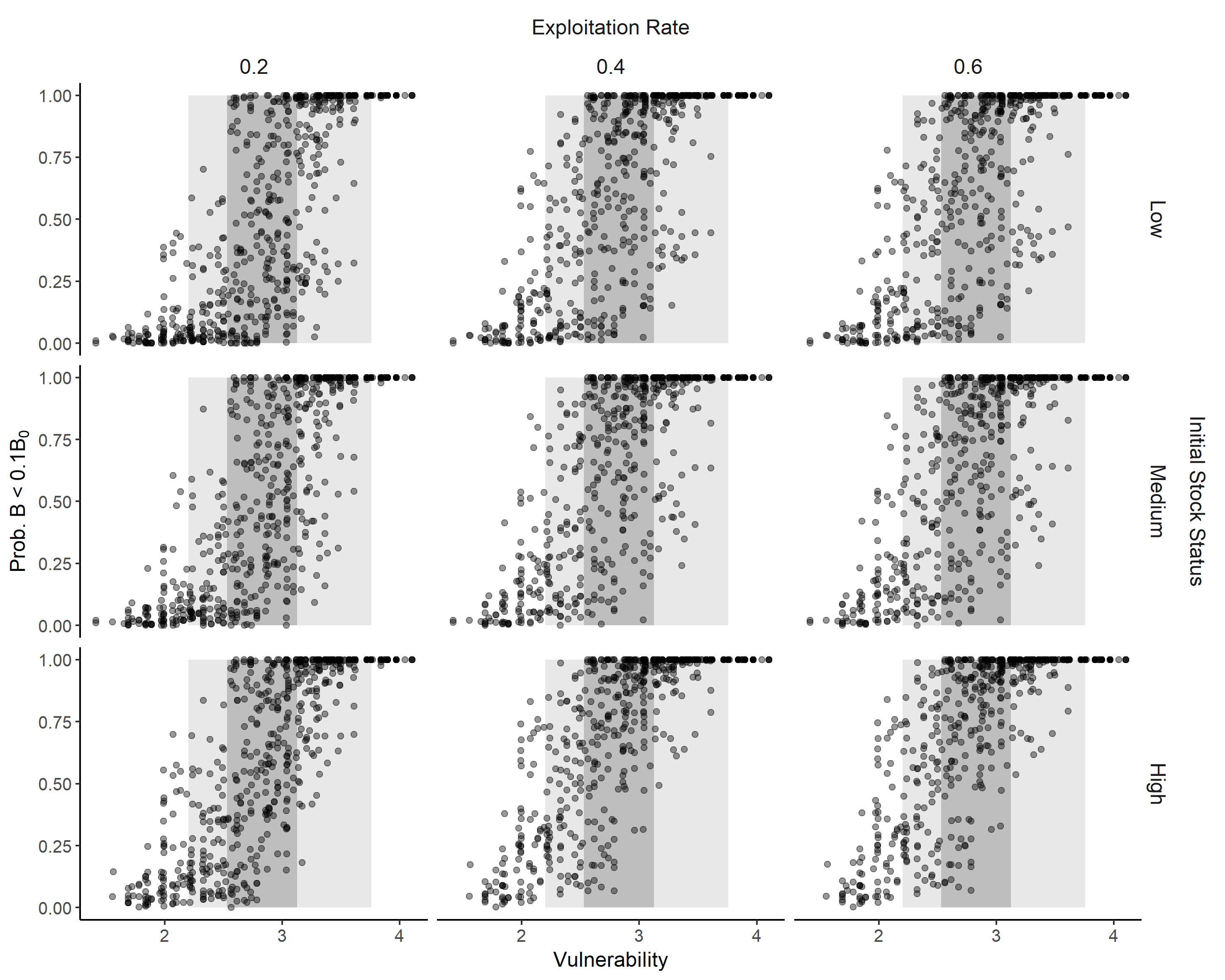

Supplement: S35 Fig — The gray shaded regions represent the 5th and 95th (light gray) and 25th and 75th (dark gray) percentiles of applications of the sPSA [17] and show that the scores for most applications fall within the mid-range values of the vulnerability score. (PNG) [file pone.0198298.s035.png]

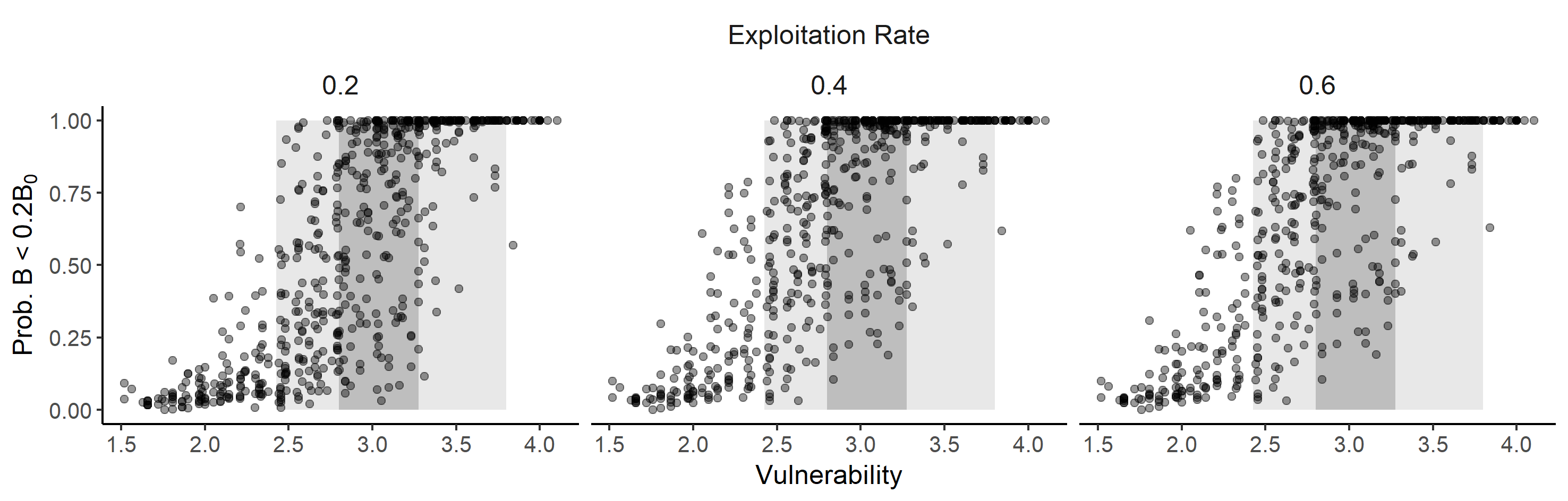

Supplement: S36 Fig — The gray shaded regions represent the 5th and 95th (light gray) and 25th and 75th (dark gray) percentiles of applications of the ePSA [20] and show that the scores for most applications fall within the mid-range values of the vulnerability score. (PNG) [file pone.0198298.s036.png]

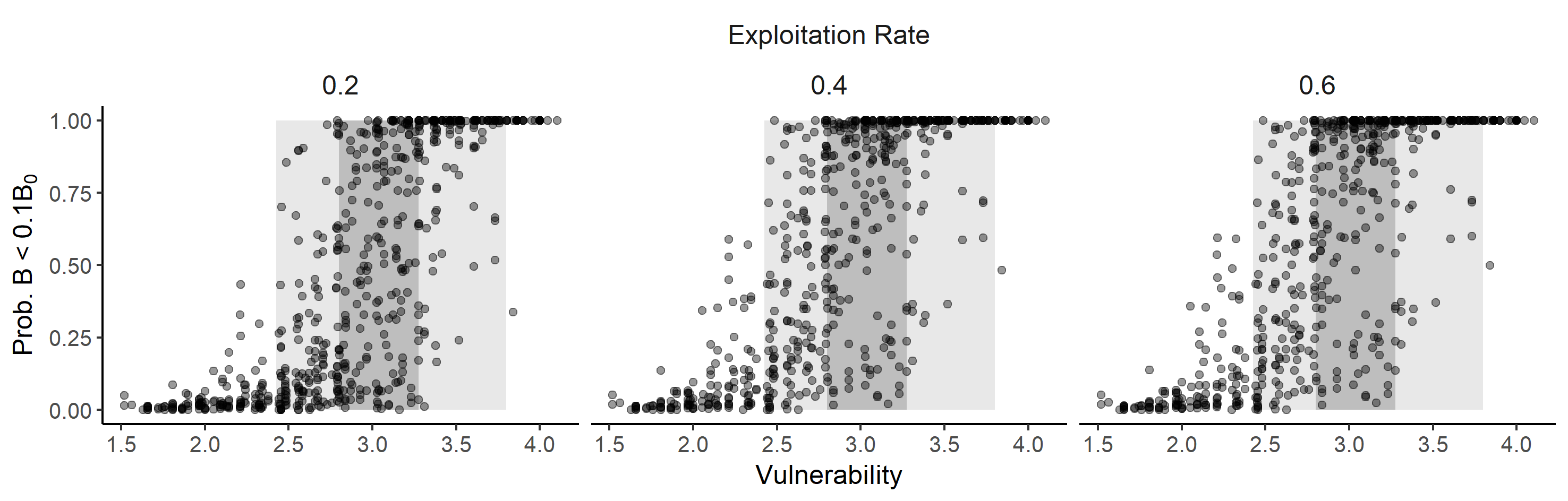

Supplement: S37 Fig — The gray shaded regions represent the 5th and 95th (light gray) and 25th and 75th (dark gray) percentiles of applications of the ePSA [20] and show that the scores for most applications fall within the mid-range values of the vulnerability score. (PNG) [file pone.0198298.s037.png]

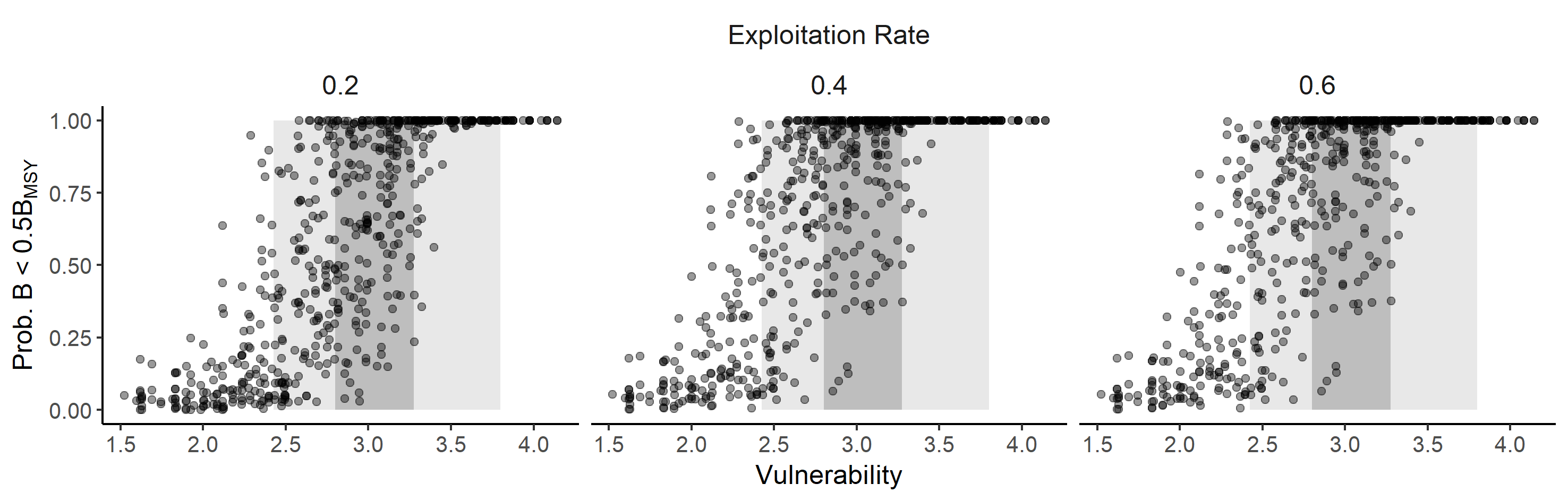

Supplement: S38 Fig — The gray shaded regions represent the 5th and 95th (light gray) and 25th and 75th (dark gray) percentiles of applications of the ePSA [20] and show that the scores for most applications fall within the mid-range values of the vulnerability score. (PNG) [file pone.0198298.s038.png]

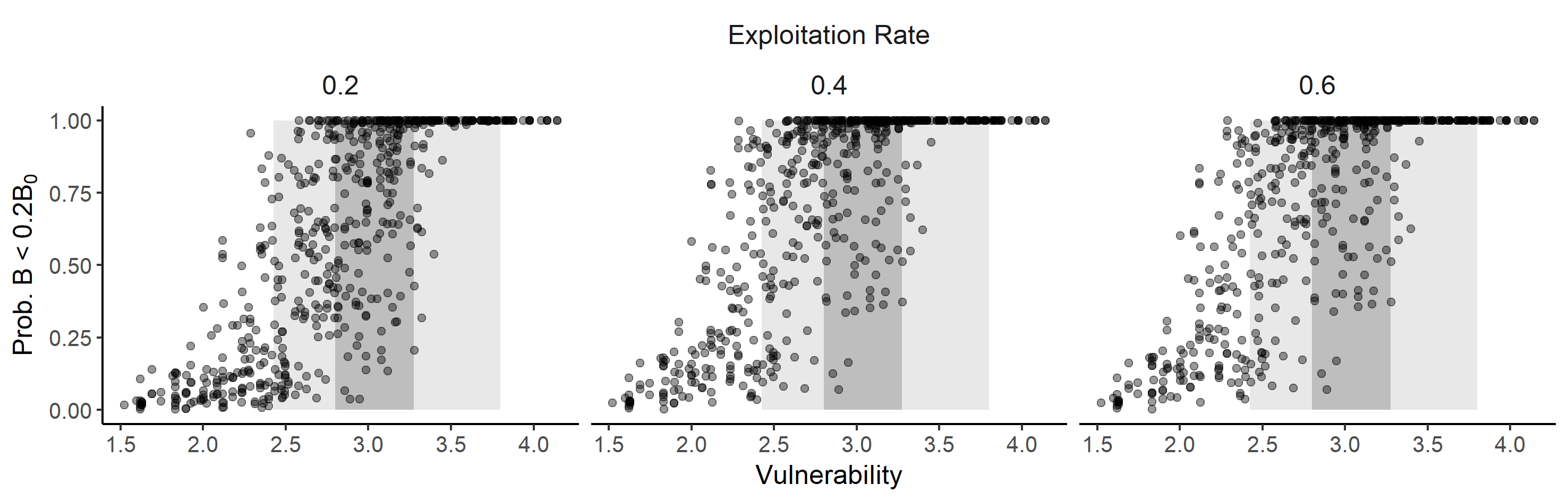

Supplement: S39 Fig — The gray shaded regions represent the 5th and 95th (light gray) and 25th and 75th (dark gray) percentiles of applications of the ePSA [20] and show that the scores for most applications fall within the mid-range values of the vulnerability score. (PNG) [file pone.0198298.s039.png]

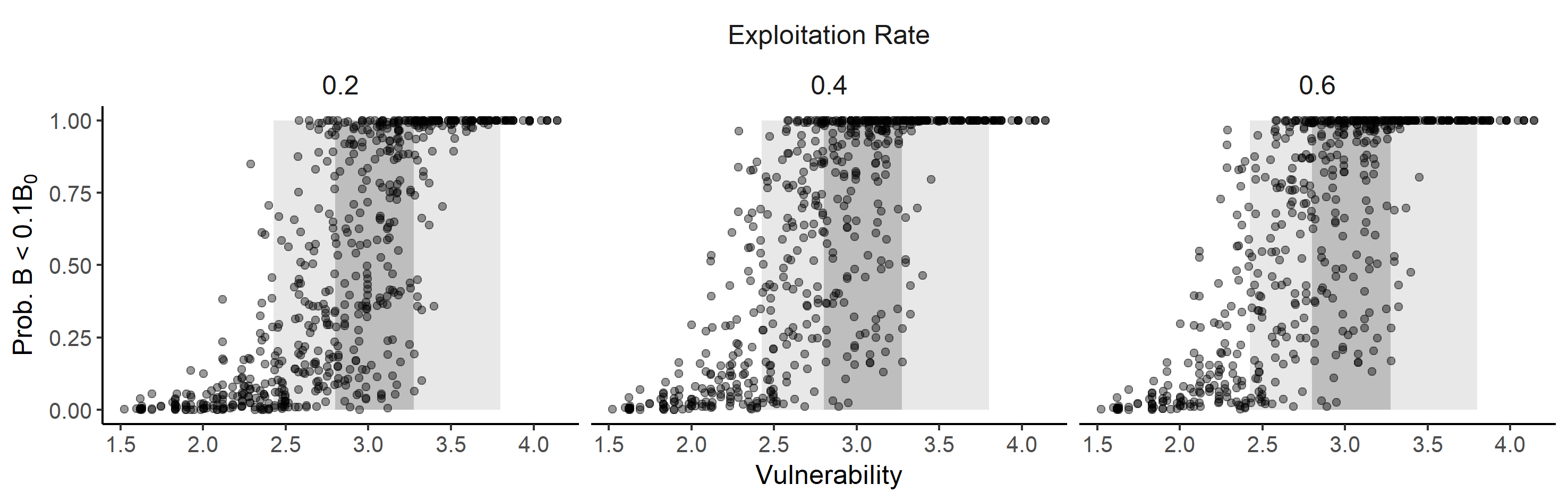

Supplement: S40 Fig — The gray shaded regions represent the 5th and 95th (light gray) and 25th and 75th (dark gray) percentiles of applications of the ePSA [20] and show that the scores for most applications fall within the mid-range values of the vulnerability score. (PNG) [file pone.0198298.s040.png]
